# Association analysis uncovers the genetic basis of general combining ability of 11 yield-related traits in parents of hybrid rice

**DOI:** 10.1093/aobpla/ply077

**Published:** 2018-12-18

**Authors:** Imdad Ullah Zaid, Weijie Tang, Jianbo He, Sana Ullah Khan, Delin Hong

**Affiliations:** 1 State Key Laboratory of Crop Genetics and Germplasm Enhancement, Nanjing Agricultural University, Nanjing, China; 2 Key Laboratory of Agricultural Water Resources, Centre for Agricultural Resources Research, Institute of Genetics and Developmental Biology, Chinese Academy of Sciences, No. 286 Huaizhong Rd, Shijiazhuang, China; 3 Soybean Research Institute, Nanjing Agricultural University, Nanjing, Jiangsu, China; 4 Queensland Alliance for Agriculture and Food Innovation, The University of Queensland, Australia

**Keywords:** Association analysis; GCA; hybrid rice, SNP discovery, SNPLDB

## Abstract

Association analysis between constructed single nucleotide polymorphism linkage disequilibrium blocks (SNPLDBs) and general combining ability (GCA) effects is a novel approach to uncover the genetic basis of GCA within the sequence genomes of parents of hybrid rice. Here, we calculated the GCA effect values of 33 parents of hybrid rice and sequenced them to identify genome-wide single nucleotide polymorphisms (SNPs). In total, 64.6 % of the uniquely mapped paired-end short reads revealed a final total of 291 959 SNPs between the 33 parental genomes and the Nipponbare reference genome. The identified SNPs were non-randomly distributed among all chromosomes of rice, whereas one-fourth of the SNPs were situated in the exonic regions with 16 % being non-synonymous. Further, the identified SNPs were merged and optimized for construction of 2612 SNPLDB markers, using linkage disequilibrium information. The single-factor analysis of variance-based association method between the constructed SNPLDB markers and GCA effects values detected 99 significant SNPLDBs for GCA of 11 yield-related traits. The associated SNPLDB markers explained 26.4 % of phenotypic variations with traits, on average. We mined 50 favourable GCA alleles at the associated SNPLDBs regions, distributed across the 33 parental genomes. The parental genomes possessed a small number of favourable GCA alleles for studied traits, with the exception of days to heading and plant height. Our results suggest that the identified GCA alleles could be used to improve the GCA performance of parents of hybrid rice through optimal crossing design. Moreover, favourable GCA alleles should be incorporated in the parental genomes through marker-assisted selection experiments, and the parental lines carrying more alleles could be utilized in breeding as superior parents for developing rice hybrids of desirable characteristics.

## Teaser

We calculated the GCA values of 33 parents of hybrid rice and sequenced them to identify SNPs. In total, 64.6 % of the uniquely mapped paired-end reads revealed a final total of 291 959 SNPs between the 33 parents and the Nipponbare reference. The identified SNPs were non-randomly distributed, whereas one-fourth of the SNPs were situated in the exonic regions with 16 % being non-synonymous. The identified SNPs were optimized for construction of 2612 SNPLDB markers, using linkage disequilibrium. The association between SNPLDB markers and GCA values detected 99 significant SNPLDBs of GCA having 26.4 % of phenotypic variations.

## Introduction

Hybrid rice exhibits both great heterosis (hybrid vigour) and yield potential compared with either inbred parent variety. Given a diverse genetic background, hybrid rice displays several good agronomic and yield characteristics that enabled it to exhibit an increased 20 % grain yield compared with other conventional *indica* and *japonica* cultivars (Qian *et al.* 2016). The gain in the yield from breeding hybrid rice first requires selection of optimal mating parents that when crossed to produce a rice hybrid with desired traits including high yield performance. This exploitation is the most critical and time-consuming phase. Over the decades, plant breeders have developed general combining ability (GCA) a measure used for selection of elite inbred lines that make the highest contributions to hybrid performance. GCA has been used to identify favourable parents in rice (Tiwari *et al.* 2011), cotton (Ahuja and Dhayal 2007), soybean (Cho and Scott 2000), maize (Kage *et al.* 2013) and wheat (Li *et al.* 1997). Relative contribution of GCA in parental can be used for selection of excellent parents in early generations (Walejkl and Rusell 1977). GCA shows a simple attitude to predict additive effects contributing to heterosis (Melchinger *et al.* 1987). GCA analysis seeks to facilitate breeding through effective and efficient selection of inbred lines for a cross based on additive and additive × additive gene effects. Moreover, GCA analysis maximize additive gene effect that increases the selection efficiency of breeders in selecting elite inbred parents with better performance (Chigeza *et al.* 2014).

In hybrid rice breeding practices, a large number of crosses are created and evaluated in a series of field trials yearly, and only a few with promising characteristics are selected and manipulated. Thus the evaluation of parental lines for GCA effects with conventional plant breeding methods is labourious, tedious and time-consuming (Liu *et al.* 2002). Therefore, it is necessary to explore modern and efficient breeding methods to improve the selection efficiency and prediction of first filial generation (F_1_) performances, i.e. by identification of GCA effects.

Earlier, several studies were designed to develop genomic resources for dissection of GCA with molecular markers, especially in rice. In this situation, the genetic foundation of GCA was uncovered by employing different categories of molecular markers. For example, Qu *et al.* (2012) identified a large number of additive effects GCA quantitative trait loci (QTLs) for 10 agronomic traits using rice recombinant inbred lines (RILs) with three testers and a backcross RIL. Similarly, two candidate genes (*OsPRR37* and *Ghd7*) of GCA for days to headings, plant height and spikelet per panicles were mapped with simple sequence repeats markers (Liu *et al.* 2015). Moreover, association analysis between molecular markers and combining ability also revealed several genomic loci of GCA of parental traits (Liang *et al.* 2010; Xie *et al.* 2016).

To improve the GCA of parental traits by employing molecular marker-assisted selection, it is essential to initially recognize the genomic locus that is strongly associated with the GCA of parental traits. In the present study, to gain multiple allelic series at an associated locus and reduce the number of false-positive SNPs, association analysis between constructed SNPLDBs and GCA effect values were suggested. Indeed, with the individual-SNP marker-based association analysis, the utility of multi-allelic SNPLDB markers exhibited more significance that enhanced the accuracy and robustness of any association analysis (Grapes *et al.* 2004; Hayes *et al.* 2007). Association analysis performed with SNPLDB markers are more beneficial, and could substantially increase the efficiency of detecting the most favourable allele (Lu *et al.* 2010). In addition, SNPLDB markers exhibit the tendency to provide new biological insights in the determination of genomic regions that could not be detected with the individual-SNP markers. Recently, the SNPLDB marker constructed based on linkage disequilibrium (LD) information was recommended for testing in association analysis and demonstrated potential applications that are valued in plant breeding experiments (Zhang *et al.* 2015; Meng *et al.* 2016).

In this study, we treated the constructed SNPLDBs as potential markers and tested them via association analysis with the calculated GCA effects values of 33 parents of hybrid rice for 11 yield-related traits. The major objectives of the present study were to (i) assess the GCA effects values of 33 parents of hybrid rice for 11 yield-related traits, (ii) discover genome-wide SNPs in parental genomes and (iii) detect significant SNPLDB markers of GCA of parental traits by association analysis.

## Methods

### Plant material and hybrid development

The experimental materials used in our study comprised eight cytoplasmic male sterile (CMS) and six restorer lines of *indica* rice, and 13 CMS and 6 restorer lines of *japonica* rice **[see ****Supporting Information—Table S1****]**. The parental lines in two sets (*indica* CMS × *indica* restorers and *japonica* CMS × *japonica* restorers) were crossed to yield the F_1_ population following the North Carolina mating designs II (NCII mating design). Seeds of each parental material were sown on the seedling beds at Jiangpu Experimental Station, Nanjing Agricultural University, Nanjing (32.06 latitude and 118.78 longitudes), China. Parental seeds were sown on 150 cm wide seedling beds with raised nursery beds of 8–10 cm height. After 30 days, rice seedlings were transplanted to puddled and water-covered paddy fields by hand, where each plot of parental material contained five rows of eight plants per row. Within the plot, plants were transplanted at 17- and 20-cm row-to-row and plant-to-plant distances. At the beginning of the flowering stage, all the CMS of *indica* and *japonica* rice were tested for pollen sterility by treating with 2 % iodine–potassium iodide stain. To pollinate the CMS parents by the corresponding restorer parents, the tip of each CMS spikelet of a selected panicle was initially cut at a slight angle using a sharp scissor that did not damage the stigma inside the spikelet. As anthesis commenced, the next morning blooming panicles of corresponding restorers were cut at the neck of the panicle and manually transferred to the CMS panicles. The panicles of restorers were rubbed with the prepared panicles of CMS such that the pollen of restorer reached the CMS spikelets properly. The pollinated panicles were bagged and tagged with blue plastic tablets accordingly. At the end of the rice crop season, the CMS panicles were harvested and the F_1_ seeds were threshed by hand.

### Phenotypic evaluation of developed hybrids and GCA effect analysis

All the developed 48 *indica* and 78 *japonica* F_1_ seeds along with their inbred parents (restorers and maintainers) were grown in a randomized complete block design. The progenies (F_1_) and parents were evaluated in three replications for 11 yield-related traits at Jiangpu Experimental Station, Nanjing Agricultural University, Nanjing, China.

Approximately 100 soaked seeds were sown on nursery beds for seedling development. The 30-day-old seedlings were transplanted to a paddy field, where each plot in a replication contained five rows of eight plants per row. The plot constituted row-to-row and plant-to-plant spacing of 17 × 20 cm. Five representative plants in a plot of each replication was randomly selected to investigate phenotypic data for 11 yield-related traits, i.e. days to heading (DH), plant height (PH), number of panicles per plant (NPPP), number of spikelets per panicles (NSPP), number of filled grains per panicles (NFGPP), panicle length (PL), seed width (SW), seed thickness (ST), seed length (SL), thousand grain weights (TGW) and grain yield per plot (GYPP).

The obtained phenotypic data of developed hybrids were subjected to analysis of variance (ANOVA) using Excel software (2007) following the statistical model (Singh and Chaudhary 1985). To determine the best parental combination among *indica* and *japonica* hybrids for grain yield per plot, mid parent-heterosis was also calculated. GCA effect value of each trait of each parental line within indica subspecies or within japonica subspecies was calculated using the formula: *g*_*i*_ = *y*_*i*_*-ŷ*, where *g*_*i*_ stands for GCA effect of parental line, *y*_*i*_ and *ŷ* each stand for the mean of crosses with same parent *P*_*i*_ and the mean of all crosses within indica groups, respectively (Zhao *et al.* 2016). Least significant difference test at *α* = 0.01 was applied to assess the significance of differences of GCA effects among CMS lines and restorer lines within subspecies.

### DNA isolation, library construction and sequencing

To construct the high-quality genomic library, young leaves of parental lines were collected and total genomic DNA was isolated with the DNA secure plant kit (Tiangen Biotech, Beijing, China) following the recommended protocol (Lu *et al.* 2016). The isolated DNA yield was assessed for purity and concentration via three different methods including agarose gel electrophoresis, spectrophotometer test (NanoDrop 2000) and Qubit 2.0 Fluorometer.

Approximately 50 µL from DNA of each sample was used for DNA library construction following the standard protocol of genotyping by sequencing (GBS) (Poland *et al.* 2012). The process of library construction was initiated via DNA digestion with two restriction enzymes (*Pst*I and *Msp*I) in an equal volume (5 μL). After DNA digestion, sample ligation was performed, and a set of 33 previously developed bar-coded adapters of 6 bp long was fixed to the 3′ and 5′ ends of DNA. Following the protocol, polymerase chain reaction was then performed to generate the GBS libraries. The fragment size length of the library was quantified up to 200 bp using the BioAnalyzer 2100 instrument (Trick *et al.* 2012). The constructed library was then sequenced on illumine Hiseq 2500 following the manufacturer’s specifications.

### Read mapping, SNP calling and annotation

Raw sequence data obtained from the sequencing machine were de-multiplexed according to the attach barcodes and were submitted to National Center for Biotechnology Information (NCBI) under accession number SRR7250921. Reads with a quality score of <Q30 were removed from the final data. The 200-bp long paired-end reads of 33 samples were then aligned against the Nipponbare reference genome. The mapping was performed with bowtie algorithm following M4, a very sensitive local parameter for short read mapping (Langmead 2010). The default parameter of Tassel-GBS software was further used for genome-wide SNP discovery (Glaubitz *et al.* 2014).

SNPs within the genes and other genomic regions were annotated by using SNPEff (4.2) software (Cingolani *et al.* 2012). SNPs defined as genic were further classified based on their position in the exon, intron, 5′ untranslated region (5′ UTR), 3′ untranslated region (3′ UTR), coding and splice-site regions. Moreover, SNPs of the coding region were divided into synonymous and non-synonymous (missense and nonsense). The total number of transition (C/T and G/A) and transversion (C/G, T/A, A/C and G/T) substitutions in each sequenced parent was also revealed in detail.

### Construction of SNPLDBs

Briefly, fastPHASE software was used to accurately predict the missing genotypes in the sequence data (Stephens and Scheet 2005). Then, Haploview software was used to define the LD blocks (Barrett *et al.* 2005). The confidence intervals block partitioning approach employed using default settings with the exception that the maximum distance and minimum major allele frequency (MAF) were set to 200 kb and 0.01, respectively (Gabriel *et al.* 2002). To construct the SNPLDB, the SNPs within a block were grouped into a single marker with a haplotype as its alleles. Each constructed SNPLDB was treated as a marker and tested in association analysis **[see ****Supporting Information—Table S2****]**. The constructed SNPLDB markers were characterized in detail by calculating their MAF, genetic diversity and polymorphism information content, using Power Marker software (v3.25) (Liu and Muse 2005).

### Association analysis

The single-factor ANOVA method of association analysis was used to test the association between constructed SNPLBD markers and GCA effect values of 11 yield-related traits. The linear model is calculated as follows:

$$y_{ij}=\mu +a_{i}+\varepsilon_{ij}$$

where *yij* is the *j*th observation of the *i*th allele at the SNPLDB under testing, *μ* is population mean, *a*_*i*_ is the effect of *i*-th allele and *εij* is random error.

All the computations of association analysis were performed using SAS PROC GLM (Release 9.1.3; SAS Institute, Cary, NC). The significant SNPLDBs on the chromosomal region were selected based on the least *P*-value (*α* = 0.01 probability level). The coefficient of determination (*R*^2^) was estimated to determine the percentage of phenotypic variation explained by each associated SNPLDB marker. Further, the genes that lie within the intervals of associated SNPLDBs were searched and annotated using the Rice Genome Annotation Project (http://rice.plantbiology.msu.edu/) database for a detailed investigation of their biological and molecular functions.

## Results

### Phenotypic evaluation of developed hybrids

The mean phenotypic performances of 11 yield-related traits in the 48 *indica* and 78 *japonica* hybrids are presented in **Supporting Information—Table S3**. Analysis of variance revealed that mean square values of all the traits among hybrids were highly significant. The *indica* hybrids revealed positive mid-parent heterotic values for GYPP. The mid-parent heterotic values ranged from -50.7 to 120.6 %, and the highest mid-parent heterosis was revealed by crossing Zhenshan 97 A×Zhenhui 084. Similarly, among the 78 *japonica* hybrids, both positive and negative heterotic values for GYPP were exhibited. The mid-parent heterotic values ranged from −40.5 to 146.2 %, and higher heterotic value was acquired in the crossing Qingkong A×Yunhui 4 hao.

### GCA effect values of 11 yield-related traits in parents

In our study, GCA effect values of 11 yield-related traits in parents varied significantly. Among the eight *indica* CMS lines, CMS 257A exhibited higher GCA effect values for PH, NSPP, NFGPP, PL and GYPP. The CMS 256A was the best general combiner for TGW. Increased GCA effect values for SW and ST were exhibited by Zhenshan 97A. Two CMS lines, YuetaiA and II-32A, exhibited significant and positive GCA effect values for SL, DH and NPPP, respectively (Table 1).

**Table 1.** General combining ability effect values of *indica* CMS and restorer lines for 11 yield-related traits

Parental lines	DH (days)	PH (cm)	NPPP	NSPP	NFGPP	PL (cm)	SW (mm)	ST (mm)	SL (mm)	TGW (gram)	GYPP (grams)
256A	−14.4g	6.2b	4.2bc	−85.0d	−57.4b	0.4ab	0.5b	0.04a	−0.07c	10.0a	62.1d
Zhenpin A	−7.4f	−16.9e	−7.5f	0.9b	−7.0b	1.0ab	−0.1b	0.03a	−0.04c	1.0b	212.3b
257A	−10.6h	27.7a	3.0c	107.4a	104.2a	5.4a	−0.3d	−0.11b	0.48b	0.4b	284.6a
II-32A	24.9a	−17.1e	5.2a	−56.1b	−40.7b	−3.0b	−0.1b	−0.12b	−1.55d	−7.1a	−425.8h
Zhenshan 97 A	11.1b	−7.9d	2.6c	−6.7b	6.2b	3.7a	0.6a	0.23a	0.00c	−0.5c	81.3c
Yuetai A	−1.3d	0.8c	1.3d	24.0b	−18.0b	−3.7b	−0.4e	−0.01a	0.89a	−2.5d	−135.8g
You 1A	−4.9e	9.3b	−0.8e	21.9b	33.2b	−2.3b	−0.2c	−0.02a	0.13c	−2.5d	35.2e
Zhong 9A	2.7c	−2.2c	−8.0f	−6.6b	−20.5b	−1.6b	−0.2c	−0.03a	0.17c	1.3b	−113.9f
Mingui 63	2.9ab	5.4b	−2.4d	−57.9c	−40.4b	−3.1b	0.21a	−0.06b	0.40a	0.3b	−11.2c
Zhenhui 084	−5.0c	−19.5d	−0.7c	−11.1b	−18.6b	7.9a	−0.28d	−0.12c	0.05b	−1.8c	35.3b
Yanhui 559	4.5a	−6.1c	1.3b	21.6a	−1.7b	−4.1b	−0.02c	−0.03bc	−0.19c	−0.4b	−22.1d
Huizi 04	−5.6c	12.4a	−2.5d	35.0a	28.6a	−1.0b	0.11b	0.16a	−0.46d	2.5a	64.1a
Hui 9368	4.9a	10.9a	7.3a	−23.2b	−8.2b	1.8b	−0.04c	−0.13c	−0.24c	−0.5b	−126.8e
Kanghui98	−4.0c	6.6b	−3.0d	35.5a	40.4a	−1.5b	0.02c	0.18a	0.44a	0.0b	60.6a

The *indica* CMS and restorer lines that contain different letters are significantly different at *P* < 0.01.

Days to heading (DH), plant height (PH), number of panicles per plant (NPPP), number of spikelets per panicles (NSPP), number of filled grains per panicles (NFGPP), panicle length (PL), seed width (SW), seed thickness (ST),seed length (SL), thousand grain weights (TGW) and grain yield per plot (GYPP).

Among the six *indica* restorer lines, restorer Hui 9368 exhibited increased GCA effect values for DH, PH and NPPP, whereas the restorer Kanghui98 exhibited increased GCA effect values for NSPP, NFGPP, ST, SL and GYPP. Three restorers, Minghui 63, Zhenhui 084 and Huizi 04, exhibited greater GCA effects for SW, PL and TGW (Table 1).

Of the 13 *japonica* CMS lines, CMS Wuyujing 3A was a good general combiner for SW, ST, TGW and GYPP. CMS 731A exhibited higher GCA effect values for SL (Table 2). CMS Liuqianxin A and 6427A exhibited positive GCA effects for NFGPP and NSPP. For NPPP and PL, CMS Qingkong A exhibited higher GCA.

**Table 2.** General combining ability effect values of *japonica* CMS and restorer lines for 11 yield-related traits

Parental lines	DH (days)	PH (cm)	NPPP	NSPP	NFGPP	PL (cm)	SW (mm)	ST (mm)	SL (mm)	TGW (grams)	GYPP (grams)
863A	6.7a	16.8c	−1.63f	14.2d	31.8d	4.9c	−0.06c	−0.21e	−0.11d	−0.74b	−17.2g
9201A	−5.1c	−8.4g	1.20e	63.7b	63.4b	6.2b	0.00c	0.00b	0.35c	−0.66b	93.4e
Xu 2A	1.4ab	−17.4h	−3.05f	−87.1	−51.3i	−11.5j	0.14c	0.12b	−0.51f	0.17b	−231.7i
Nanjing 46A	8.6a	2.2e	6.28b	−1.9e	14.8e	2.9d	−0.17c	−0.06c	−0.33e	2.01b	−109.6h
731A	−2.4b	−21.9i	−1.59f	−78.5h	−36.9h	−2.0f	−0.12c	0.13b	0.98a	1.76b	61.4f
Liuqianxin A	1.1bc	−5.5f	0.36ef	−41.8f	129.1a	3.2d	0.18b	−0.07c	0.21c	−3.33c	11.5g
6427A	3.7a	10.5d	−8.47g	128.4a	−37.7h	2.1e	0.29a	0.06b	−0.58f	−1.58b	191.1b
Zhendao 88A	6.4a	−4.8f	3.03d	−8.5e	−37.7h	−3.5g	−0.04c	0.03b	−0.62f	0.92b	1.3g
Qingkong A	−19.6d	40.0b	7.41a	61.7b	2.3f	6.8a	−0.21d	−0.14d	−1.35f	−0.16b	144.6c
Yueguang A	−3.6b	59.3a	4.57c	40.4c	−11.7g	6.5a	−0.44e	−0.27f	0.50b	−3.24c	101.9d
Wuqiang A	11.4a	−16.7h	−2.00f	45.7c	−5.0g	−7.5i	−0.17c	0.02b	0.50b	0.76b	−146.3h
Wuyujing 3A	5.6a	−18.1h	−3.42f	12.7d	50.2c	−5.6h	0.32a	0.31a	0.28c	3.26a	229.0a
Liuyan 189A	−14.3d	−36.0j	−2.68f	−148.8i	−110.5j	−2.5f	0.26a	0.09b	0.68a	0.84b	−329.4j
C418	−3.9b	2.5b	1.7a	47.5a	57.6a	0.98a	0.14a	0.00b	0.40a	0.19b	168.5a
Ninghui8hao	−7.6b	−8.8d	−2.6b	−50.4d	−38.6b	−0.36b	0.04b	0.03b	−0.21c	0.07b	40.1c
Yunhui 4 hao	5.4a	−10.1d	−3.3b	21.9b	5.1c	0.49a	0.01b	−0.05b	−0.31c	−2.47d	−179.0e
Zhehui 315	2.1a	−3.6c	1.3a	51.9a	38.3d	0.11b	−0.21d	−0.06b	0.01b	1.22a	−62.4d
Yanhui R50	4.0a	10.0a	1.7a	−67.4e	−67.5e	−0.86c	−0.10c	0.10a	0.41a	1.92a	100.3b
Xiushui 04R	2.1ab	10.0a	1.1a	−3.5c	5.1c	−0.36b	0.12a	−0.02b	−0.30c	−0.93c	−67.6d

The *japonica* CMS and restorer lines followed by different letters are significantly different at *P* < 0.01.

Days to heading (DH), plant height (PH), number of panicles per plant (NPPP), number of spikelets per panicles (NSPP), number of filled grains per panicles (NFGPP), panicle length (PL), seed width (SW), seed thickness (ST),seed length (SL), thousand grain weights (TGW) and grain yield per plot (GYPP).

Our result confirmed that among six *japonica* restorer lines, the restorer C418 exhibited desirable GCA effect values for NPPP, NFGPP, SW, PL and GYPP (Table 2). Restorer Yanhui R50 exhibited increased GCA effects for PH, NPPP, ST, SL and TGW. The restorer Yunhui 4 hao and Zhehui 315 recorded positive GCA effects for DH, NSPP and GYPP.

### Mapping of short reads

After de-multiplexing of attached barcodes and the removal of unwanted sequences, the quality sequenced files were then assessed using FastQC software. The mapping results revealed greater than 19 million (19 219 593) of total short reads. Of the mapped reads, 64.6 % (12 431 703) aligned exactly once, and the remaining 31 % (5 961 011) aligned more than once onto to the 12 chromosomes of the Nipponbare genome. Overall, a 95.7 % alignment rate was recorded. In addition, 4.3 % (826 879) of the short reads remained unmapped onto any part of the Nipponbare reference genome.

### Detection and distribution of SNPs in parental genomes

SNPs in the 33 parental genomes were identified by individual comparison with the Nipponbare reference genome. Within the uniquely mapped sequenced reads of parental genomes, we identified a total of 292 074 SNPs at 30 081 genomic positions, including 190 705 and 101 369 in *indica and japonica* parents, respectively. Despite having the same coverage of the genomes, more SNPs were identified in *indica* rice compared with *japonica* rice. The identified SNPs varied among parental genomes. For example, Wuyujing 3A has a minimum of 1986 SNPs, and Zhenpin A has a maximum of 17 563 SNPs **[see ****Supporting Information—Table S4**]. The discovered SNPs were distributed non-randomly over the 12 chromosomes of rice, where chromosome 1 of all the sequenced genomes contained the most SNPs (38 567), whereas chromosome 9 contained the least SNPs (15 728).

### Annotation of identified SNPs

The Nipponbare rice was used as a reference genome to reveal the distribution of identified SNPs within the various genomic regions. Among the identified SNPs, the majority 167 814 (56.9 %) of SNPs were detected in intergenic regions. Altogether, 82 183 (28.6 %) and 46 076 (14.9 %) SNPs were situated in exon and intron regions, respectively. In total, 31 674 (10.7 %) SNPs in the coding region were classified as synonymous, whereas 45 193 (16 %) SNPs were non-synonymous. The 3′ UTR and 5′ UTR regions possessed 19 687 (6.6 %) and 12 491 (5.45 %) SNPs, respectively, whereas the remaining 2903 (0.9 %) were found in the spliced region of all parental genomes **[see ****Supporting Information—Table S5****]**.

### Analysis of transitions and transversions SNPs

We classified the identified SNPs into transitions and transversion substitutions using the SNPEff software. A higher frequency of transition substitutions was examined compared with transversion substitutions. The number of C/T transition was increased compared with the G/A transitions **[see ****Supporting Information—Table S6****]**. Among the identified SNPs of indica rice parents, A/C transversions evolved more frequently compared with C/G, T/A and G/T, whereas C/G transversions were relatively increased compared with A/C and G/T among the SNPs of japonica rice parents.

### Association analysis between SNPLDB markers and GCA effect values

Association analysis between 2612 constructed SNPLDB markers and GCA effect values of 33 parental lines confirmed a total of 99 significant SNPLDBs associated with GCA for 11 yield-related traits (Table 3), explaining 26.4 % of phenotypic variation on averages. The significant SNPLDBs were distributed all over the 12 chromosomes of rice. The number of associated SNPLBDs for each trait varied and exhibited both negative and positive effects. Furthermore, the biological and molecular functions of detected genes within the intervals of associated SNPLDBs were revealed in detail.

**Table 3.** List of significant single nucleotide polymorphism linkage disequilibrium block markers associated with general combining ability effect values of 11 yield-related traits

Trait	SNPLBDs	Chromosome	*P*-value	*R* ^*2*^ (%)
DH	1_BLOCK_28934801_29133392	1	0.009	33.2
	2_BLOCK_23246549_23402926	2	0.006	22.2
	4_BLOCK_11861449_12047086	4	0.005	30.0
	4_BLOCK_14047340_14210685	4	0.009	23.9
	5_BLOCK_6791185_6982283	5	0.003	27.8
	5_BLOCK_27886769_28020620	5	0.004	33.9
	7_BLOCK_27728152_27917280	7	0.008	32.6
	11_BLOCK_19263139_19263152	11	0.006	26.2
	11_BLOCK_23861754_23986595	11	0.003	28.2
PH	2_BLOCK_35817740_35924060	2	0.008	23.6
	3_BLOCK_9933834_10133555	3	0.004	23.6
	S4_16441661	4	0.002	30.5
	6_BLOCK_738449_922752	6	0.009	20.4
	6_BLOCK_970592_1159740	6	0.008	30.1
	6_BLOCK_2655251_2849385	6	0.001	30.2
	S7_10979899	7	0.003	27.7
	7_BLOCK_24627656_24806954	7	0.003	25.9
	7_BLOCK_26549570_26746263	7	0.003	32.0
	8_BLOCK_6251405_6277158	8	0.007	22.6
	9_BLOCK_12636807_12697367	9	0.009	21.1
	S10_2115860	10	0.004	28.2
	10_BLOCK_5442582_5562381	10	0.002	29.3
	10_BLOCK_10531770_10728242	10	0.001	30.0
NPPP	2_BLOCK_22143883_22260164	2	0.008	21.9
	S6_7843151	6	0.005	22.5
	9_BLOCK_17535393_17676486	9	0.004	24.9
	9_BLOCK_19575308_19760644	9	0.004	25.7
	11_BLOCK_17820001_17945021	11	0.004	24.7
	11_BLOCK_18431697_18435610	11	0.003	27.2
	11_BLOCK_18901416_19072571	11	0.002	28.8
	11_BLOCK_19309311_19309329	11	0.009	24.4
	11_BLOCK_23861754_23986595	11	0.004	25.6
NSPP	7_BLOCK_9972678_9972706	7	0.008	21.7
	S7_26929982	7	0.009	23.6
NFGPP	2_BLOCK_24571861_24661819	2	0.005	22.4
	2_BLOCK_25142102_25304942	2	0.006	23.3
	2_BLOCK_25359391_25409193	2	0.007	22.3
	2_BLOCK_26014682_26204848	2	0.005	22.4
	4_BLOCK_19576610_19769633	4	0.007	21.2
	7_BLOCK_10991803_11175112	7	0.001	32.7
	9_BLOCK_11128244_11230987	9	0.005	22.4
	11_BLOCK_18121821_18288410	11	0.006	25.3
	11_BLOCK_22899060_23033776	11	0.005	23.3
	12_BLOCK_18717643_18913133	12	0.005	33.3
	12_BLOCK_19106376_19183058	12	0.005	22.4
PL	2_BLOCK_35591725_35790248	2	0.007	26.8
	2_BLOCK_35817740_35924060	2	0.010	22.4
	4_BLOCK_31842220_32019553	4	0.001	29.7
	4_BLOCK_32144497_32252127	4	0.003	28.2
	4_BLOCK_33128044_33326841	4	0.008	25.5
	S7_10979899	7	0.007	24.2
	8_BLOCK_16164808_16356444	8	0.005	23.2
	9_BLOCK_14599124_14797034	9	0.004	24.2
	10_BLOCK_10329302_10485970	10	0.008	20.7
	12_BLOCK_7503930_7561908	12	0.005	35.2
	12_BLOCK_14219148_14220620	12	0.007	23.7
	12_BLOCK_14665324_14675306	12	0.009	20.0
SW	7_BLOCK_10991803_11175112	7	0.008	22.4
	9_BLOCK_19806236_19979493	9	0.005	32.7
	10_BLOCK_22070293_22270204	10	0.004	24.8
ST	1_BLOCK_10275866_10473381	1	0.009	20.3
	2_BLOCK_24571861_24661819	2	0.009	21.3
	4_BLOCK_31842220_32019553	4	0.007	21.9
	S6_27632618	6	0.004	24.8
	7_BLOCK_5179524_5348724	7	0.009	24.6
	7_BLOCK_24385360_24576268	7	0.008	25.2
	S11_19707591	11	0.007	24.4
	11_BLOCK_20480700_20564349	11	0.003	25.5
SL	2_BLOCK_12695457_12894785	2	0.007	34.0
	3_BLOCK_10477564_10651116	3	0.009	29.6
	4_BLOCK_29858777_29965891	4	0.009	37.6
	5_BLOCK_6791185_6982283	5	0.005	25.5
	11_BLOCK_23861754_23986595	11	0.004	27.4
	S12_7434458	12	0.009	19.9
	12_BLOCK_12967864_13140942	12	0.005	24.4
	12_BLOCK_13663061_13861944	12	0.004	27.4
	S12_13914348	12	0.005	23.8
TGW	1_BLOCK_28709309_28893652	1	0.008	26.9
	S1_28920012	1	0.008	23.2
	1_BLOCK_31318752_31502481	1	0.007	25.4
	S3_6948281	3	0.010	22.9
	3_BLOCK_9599101_9781950	3	0.009	24.5
	3_BLOCK_10991463_11168887	3	0.004	26.3
	3_BLOCK_23155569_23343573	3	0.009	23.2
	3_BLOCK_32969173_33157926	3	0.010	26.8
	5_BLOCK_6791185_6982283	5	0.006	24.7
	5_BLOCK_29004623_29203515	5	0.007	21.1
	9_BLOCK_15762553_15959818	9	0.006	27.1
	9_BLOCK_20925868_21081274	9	0.004	24.1
	11_BLOCK_20176616_20356516	11	0.009	24.5
	11_BLOCK_23861754_23986595	11	0.005	26.2
	11_BLOCK_26916905_27078112	11	0.007	28.9
GYPP	3_BLOCK_6520556_6531124	3	0.008	52.3
	3_BLOCK_14595705_14789758	3	0.006	40.3
	5_BLOCK_6791185_6982283	5	0.007	23.7
	10_BLOCK_426788_526921	10	0.009	24.5
	11_BLOCK_23861754_23986595	11	0.005	26.1
	12_BLOCK_13997092_14180506	12	0.010	46.9
	12_BLOCK_14219148_14220620	12	0.003	29.7

Days to heading (DH), plant height (PH), number of panicles per plant (NPPP), number of spikelets per panicles (NSPP), number of filled grains per panicles (NFGPP), panicle length (PL), seed width (SW), seed thickness (ST), seed length (SL), thousand grain weights (TGW) and grain yield per plot (GYPP).

### SNPLDBs associated with GCA of days to heading

Nine SNPLDBs located on six different chromosomes (Chr1, Chr2, Chr4, Chr5, Chr7 and Chr11) displayed significant associations with the GCA effect value of DH. Of these associated SNPLDBs, the maximum number was found on chromosomes 4, 5 and 11. The associated SNPLDBs of GCA of DH explained phenotypic variance in the range of 22.2–33.9 %. Overall, five exhibited negative effects, whereas four exhibited positive effects on GCA of DH.

We detected a total of 64 genes within the intervals of nine associated SNPLDB regions of GCA of DH. Gene Ontology analysis revealed that the main biological function of these genes was involved in reproduction and embryo development, transport activity, cellular component organization, bio-synthesis processes and protein metabolic processes. Similarly, at the level of molecular function, the genes were involved in oxygen binding, hydrolase, catalytic, transferase and kinase activities.

### SNPLDBs associated with GCA of plant height

A total of 14 significant SNPLDBs, mainly distributed on eight different chromosomes (Chr2, Chr3, Chr4, Chr6, Chr7, Chr8, Chr9 and Chr10) were associated with the GCA effect value of PH. Maximum associated SNPLDBs were located on chromosomes 6, 7 and 10. The phenotypic variation explained by each SNPLDB ranged from 20.4 to 32 %. Overall, seven exhibited negative effects, whereas seven exhibited positive effects with the GCA of PH.

We detected a total of 72 genes within the intervals of associated SNPLDB of GCA of PH. The biological functions of detected genes included flower development, cell differentiation, carbohydrate metabolic, signal transduction and metabolic processes. Similarly, the molecular functions of genes included transporter, protein binding, structural molecule, nucleotide binding and catalytic activities.

### SNPLDBs associated with GCA of number of panicles per plant

Nine SNPLDBs on four different chromosomes (Chr2, Chr6, Chr9 and Chr11) exhibited relationships with the GCA effect value of NPPP. The phenotypic variation caused by the associated SNPLDBs ranged from 21.9 to 28.8 %. Of all significant SNPLDBs, five exhibited negative effects, whereas four exhibited positive effects with the GCA of the trait.

We detected a total of 41 genes inside the associated SNPLDBs of GCA of NPPP. The main biological functions of genes included in post-embryonic development, biosynthetic, metabolic, response to stress and biosynthetic processes. In addition, the molecular function of detected genes included RNA and oxygen binding, sequence-specific DNA binding transcription factor activity, response to extracellular stimulus and nuclease activity.

### SNPLDBs associated with GCA of number of spikelets per panicles

Two SNPLDBs situated on chromosome 7 exhibited significant associations with the GCA effect value of NSPP. The phenotypic variances of associated SNPLDBs were 21.7 and 23.6 %, separately.

We detected one gene within the interval of associated SNPLDBs of GCA of NSPP. The protein product (Mak16 protein domain containing protein) of the gene was expressed and exhibited a specific biological and molecular function.

### SNPLDBs associated with GCA of number of filled grains per panicles

A total of 11 significant SNPLDBs for the GCA effect value of NFGPP were identified. The SNPLDB markers were distributed on six different chromosomes (Chr2, Chr4, Chr7, Chr9, Chr11 and Chr12). The phenotypic variance explained by the associated SNPLDBs ranged from 21.1 to 33.3 %. Of these SNPLDBs, nine exhibited negative effects, whereas two exhibited positive effects with the GCA of NFGPP.

We identified 47 genes inside the SNPLDB regions of GCA of NFGPP. Gene Ontology revealed that the main biological functions of detected genes included cell growth, metabolic process, response to stress, cellular component organization and protein modification processes. Similarly, the molecular functions of these genes included DNA binding, sequence-specific DNA binding transcription factor activity, transporter activity, kinase activity and nucleic acid binding.

### SNPLDBs associated with GCA of panicle length

A total of 12 SNPLDBs, mainly distributed on seven different chromosomes (Chr2, Chr4, Chr7, Chr8, Chr9, Chr10 and Chr12) were associated with the GCA effect value of PL. Most of the SNPs were located on chromosomes 4 and 12. Phenotypic variation ranged from 20 to 35.2 %. In total, six SNPLDBs exhibited negative effects, whereas the remaining six exhibited positive effects.

We detected a total of 64 genes inside the intervals of associated SNPLDBs of GCA of PL. Gene Ontology analysis revealed that the main biological functions included pollen–pistil interaction, multicellular organismal development, signal transduction, cellular component organization and catabolic process. Similarly, the molecular functions of these genes included hydrolase, transferase and receptor activities.

### SNPLDBs associated with GCA of seed width

Three SNPLDBs distributed over chromosomes 7, 9 and 10 exhibited significant associations with the GCA effect value of SW. The phenotypic variations explained by SNPLDBs were 22.3, 24.7 and 32.7 %, respectively.

We confirmed the presence of 22 genes within associated SNPLDB regions. Gene Ontology analysis revealed that the main biological functions of these genes included metabolism, post-embryonic development, multi-cellular organismal development, response to endogenous stimulus and protein modification processes. Similarly, regarding molecular function, the genes exhibited catalytic, DNA binding, transporter, lipid binding and receptor activities.

### SNPLDBs associated with GCA of seed thickness

We identified eight significant SNPLDBs for GCA effect value of ST distributed over six different chromosomes (Chr1, Chr2, Chr4, Chr6, Chr7 and Chr11). The phenotypic variations explained by each SNPLDB ranged from 20.2 to 25.5 %. Among the associated SNPLDBs, two exhibited negative effects, whereas six exhibited positive effects with the GCA of ST.

We identified 43 genes within the intervals of associated SNPLDBs of GCA of ST. The biological functions of these genes included cell differentiation, cell growth, carbohydrate metabolic, multicellular organismal development and cellular processes. Similarly, regarding molecular function, the genes were involved in mitochondrial, catalytic, lipid binding, catalytic and hydrolase activities.

### SNPLDBs associated with GCA of seed length

Nine SNPLDBs distributed across six different chromosomes (Chr2, Chr3, Chr4, Chr5, Chr11 and Chr12) revealed significant associations with the parental GCA effect value of GL. The phenotypic variations caused by each SNPLDB ranged from 19.8 to 37.6 %. Of these, two exhibited negative effects, whereas seven exhibited positive effects with the GCA of SL.

We detected 87 genes within associated SNPLDBs of GCA for SL. Gene Ontology analysis revealed that their main biological functions included cellular, biosynthetic, post-embryonic development, response to abiotic stimulus and metabolic processes. Similarly, regarding molecular function, the genes exhibited catalytic, transporter, RNA binding and hydrolase activities.

### SNPLDBs associated with GCA of thousand grain weights

A total of 15 significant SNPLDBs, distributed on chromosomes 1, 3, 5, 9 and 11 were associated with the GCA effect value of TGW, and their phenotypic variations ranged from 21 to 28.9 %. Among the associated SNPLDBs, eight exhibited negative effects, whereas seven exhibited positive effects with GCA of TGW.

We detected 111 genes inside the associated SNPLDBs of GCA of TGW. The biological functions of these genes included cell growth, pollination, translation, cellular homeostasis and post-embryonic development. Similarly, the molecular function of these genes included kinase, hydrolase, nucleic acid binding and nucleotide binding activities.

### SNPLDBs associated with GCA of grain yield per plot

Seven SNPLDBs exhibited significant associations with the parental GCA effect value of GYPP. The phenotypic variations caused by associated SNPLDBs ranged from 23.7 to 52.3 %. Four exhibited negative effects, whereas three had positive effects with GCA of GYPP.

We detected 52 genes within seven associated SNPLDBs of GCA of GYPP. Their biological functions included protein metabolic, biosynthesis, cell death and post-embryonic development. Similarly, their molecular function included oxygen and RNA binding and catalytic activity.

### Selection of optimal parental lines based on the presence of favourable GCA alleles

In our study, alleles with positive GCA values for parental traits were considered as favourable SNPLDB alleles. The total number of favourable SNPLDB alleles for GCA of DH, PH, NPPP, NSPP, NFGPP, PL, SW, ST, SL TGW and GYPP were 5, 7, 4, 1, 2, 6, 2, 6, 7, 7 and 3, respectively **[see ****Supporting Information—Table S7****]**. Among the eight *indica* CMS lines, CMS II-32A possessed a maximum number of 7, 5, 4 and 2 positive GCA alleles for PH, SL, TGW and GYPP, respectively. Similarly, of the six *indica* restorer lines, restorer Minghui 63 harboured 3 and 7 positive GCA alleles for DH and PH. The restorer Huizi 04 contained 4 GCA alleles for SL. The restorer Hui 9368 harboured 2 and 3 favourable GCA alleles for NPPP and SL.

Subsequently, among the 13 *japonica* CMS lines, CMS Qingkong A and Yueguang A exhibited a maximum number favourable GCA alleles for DH and NPPP. CMS 6427A and Liuqianxin A contained 1 and 2 favourable alleles of NSPP and NFGPP, respectively. In the sequenced genome of Yueguang A, we identified 4 and 3 positive GCA alleles of PL and ST, respectively. CMS Zhendao 88A had five favourable GCA alleles of SL. Among the six *japonica* restorer lines, Yunhui 4 hao harboured alleles in the genome related to the GCA enhancement of NPPP, PL and GYPP. The restorer Xiushui 04R and Zhehui 315 contained a maximum of three GCA alleles for ST and SL.

## Discussion

In 1942, Sprague and Tatum defined GCA as the mean performance of an inbred parent involved in a series of hybrid combinations (Sprague and Tatum 1942). GCA information is one of the novel criteria used to designate inbred parents based on the performances of their offspring, usually the F_1_. In our study, the developed 48 and 78 crosses of *indica* and *japonica* rice were used to estimate the GCA effects of 11 yield-related traits. The developed crosses were performed by following the North Carolina mating design II (Zhang *et al.* 2010). The North Carolina mating design II is a factorial-based mating design that estimates parental variance separately. This design is widely utilized for GCA effect values of mating parents (Qu *et al.* 2012; Nduwumuremyi *et al.* 2013; Wang *et al.* 2017). The best advantage of the NC II mating design compared with other mating designs include its independent estimation of GCA effects, which allows the determination of maternal effect and heritability based on male variance. Hence, the parental lines with elite GCA effects in our study may be good general combiners to improve grain yield traits (Bagheri and Jelodar 2010). Although conventional breeding methods has certain limitations and drawbacks and thus little achievements have been so in estimating parental GCA by means of field trials (Smith *et al.* 2008). The on-going progress in genomics has provided numerous types of molecular markers for targeting genes, QTLs or genome regions of parental GCA. The molecular dissection of GCA can not only elucidate the GCA pattern but can also provide targets to improve its effects. Previous breeding practices have transferred and accumulated the identified favourable GCA alleles across generations. For instance, the combining ability of the rice restorer (Minhui63) was improved by incorporation alleles with favourable combining abilities (Liu *et al.* 2004).

The efforts to construct SNPLDB markers from large genome sequence data have provided more information for various crops such as in Chinese soybean (Zhang *et al.* 2015), rice (Lestari *et al.* 2011), maize (Van Inghelandt *et al.* 2012) and wheat (Haile *et al.* 2013). The proposed association analysis in the current study using SNPLDB markers revealed more consequences that could substantially increase the efficiency of detecting superior genomic regions (Lu *et al.* 2010; Yan *et al.* 2011). In this study, the genomic positions of associated SNPLDBs were compared with the reported genomic positions of GCA. The associated SNPLDBs of GCA of PH and ST on chromosomes 6, 7 and 11 were close to the chromosomal regions of previously identified alleles of PH and ST with combining abilities (Zaid *et al.* 2017). Additionally, most of the associated SNPLDBs were located in adjacent intervals of the same chromosome that used a diverse set of ILs (Xiang *et al.* 2016). Moreover, SNPLDBs of GCA of DH, PH, PL, NFGPP and NSPP were also mapped on same chromosome as previously reported (Qu *et al.* 2012). These results convincingly confirmed that our associated SNPLDBs could represent the variants responsible for parental GCA. In addition, a greater number of associated SNPLDBs were detected on chromosomes 7, 11 and 12. It is possible that this finding potentially occurred due to the presence of potential GCA coverage in the potential SNPLDB regions.

**Figure 1.**
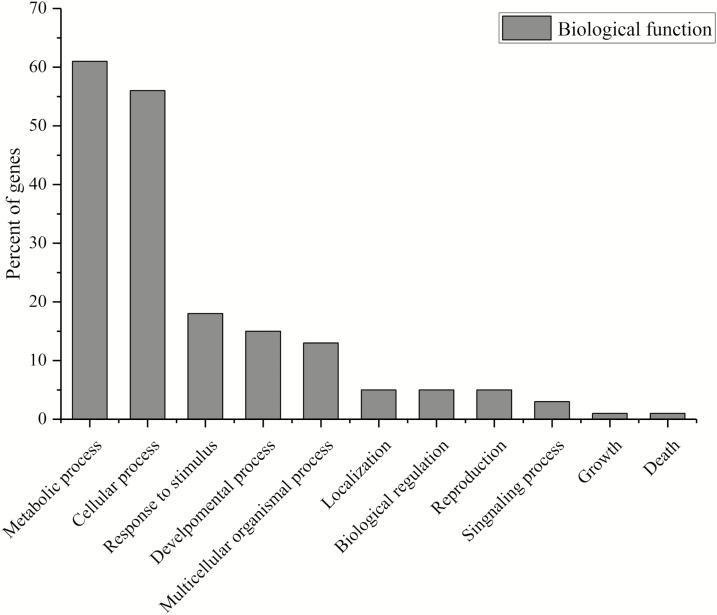
Major biological functions of detected genes within the interval regions of associated single nucleotide polymorphism linkage disequilibrium blocks.

**Figure 2.**
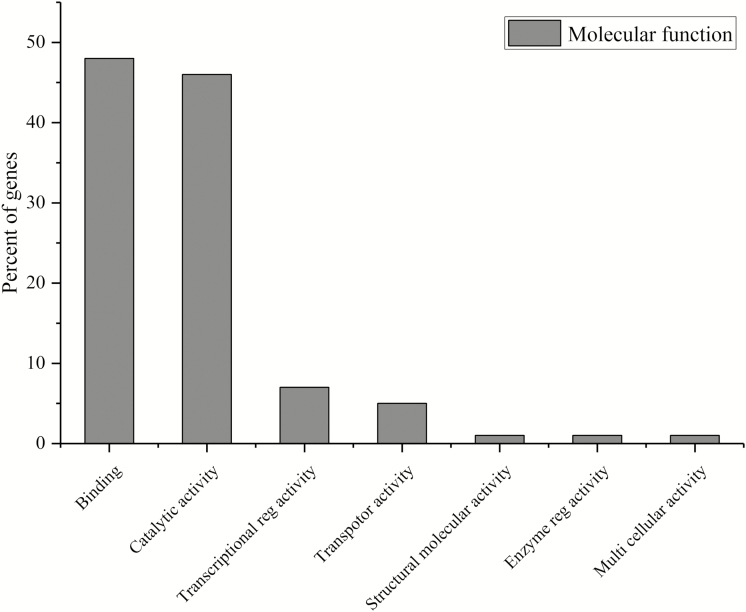
Major molecular functions of detected genes within the interval regions of associated SNPLDBs.

The biological functions of the 603 genes (**Supporting Information—Table S8** and Fig. 1) situated within the interval of 99 associated SNPLDBs revealed the greatest correspondence to metabolism (92, 61 %), followed by cellular process (51, 56 %) and response to stimulus (36, 18 %). Lower correspondence was noted for genes involved encoding reproduction, cell death, localization and signalling (Fig. 1). Genes functioning in binding (63, 48 %), catalytic (54, 46 %) and transaction regulator activities (22, 7 %) were detected via molecular-based annotation (Fig. 2). Within the genomic regions of SNPLDBs of GCA of NFGPP, we identified 10 reported genes. Of them, *LOC_Os02g43180.1* belongs to the aldehyde dehydrogenase protein family. In rice, this protein family is involved in stress tolerance control, embryo development, seed viability and maturation processes (Kotchoni *et al.* 2010). Similarly, *LOC_Os04g55920.1* within the intervals of the associated SNPLDBs for GCA of PL belongs to the TIFY gene family that encodes a plant growth hormone (jasmonic acid), that regulates spikelet growth in rice (Ye *et al.* 2009; Cai *et al.* 2014). In this study, we did not validate the expression of detected genes. However, high-throughput technologies, such as qRT–PCR, microarrays, transcriptome profiling and protein analyses can be used to monitor the expression of these genes simultaneously. This information might provide details for comprehensive snapshots of the dynamic patterns of gene expression that can validate the relationship between detected genes and GCA effects.

In conclusion, the identified GCA alleles will increase the selection efficiency of rice breeders. The favourable GCA allele in an inbred line could accumulate with cycles of selection and phenotype enhancement. Subsequent inter-mating of parental lines containing favourable GCA alleles through recurrent selection techniques could develop new hybrid cultivars with elite performances.

## Sources of Funding

Funding support was provided by a grant from the National Science Foundation of China (31571743 and 31671658), Chinese national ‘863’ program (2010AA101301), and a grant from the doctoral fund of the Educational Ministry of China (20130097110001).

## Contributions by the Authors

D.H. and I.U.Z. conceived the idea and designed the experiment; I.U.Z., W.T., and S.U.K. contributed to the data collection and H.J. constructed SNPLDBs; I.U.Z. analysed the data and wrote the paper.

## Conflict of Interest

None declared.

## Supporting Information

The following supporting information is available in the online version of this article—

**Table S1.** Plant materials used in present experiment.

**Table S2.** Total number of constructed SNPLDBs.

**Table S3.** The developed 48 indica and 78 japonica crosses and their mean phenotypic performances for 11 yield-related traits

**Table S4.** Number of identified SNPs on individual chromosomes of the indica and japonica cytoplasmic male sterile and restorer lines

**Table S5.** Annotation of identified SNPs between indica and japonica cytoplasmic male sterile and restorer lines

**Table S6.** Classifications of SNPs detected in indica and japonica cytoplasmic male sterile and restorer lines

**Table S7.** Parental genomes with favorable GCA alleles of 11 yield-related traits

**Table S8.** Annotations of detected genes within the intervals of associated SNPLDBs for GCA of 11 yield related traits

Supplimentary Material
