# Supplementary material for: Association analysis uncovers the genetic basis of general combining ability of 11 yield-related traits in parents of hybrid rice
Source: AoB Plants. 2018 Dec 18;11(1):ply077. doi: 10.1093/aobpla/ply077 (PMC6343818; doi:10.1093/aobpla/ply077)

Supporting Information

Supporting Information Table S1- Plant materials used in present experiment

Name	Material type	Developed year	Subspecies
256A (WA type)	CMS	2010	indica
Zhenpin A (WA type)	CMS	2010	indica
257A (WA type)	CMS	2010	indica
II-32A (ID type)	CMS	2013	indica
Zhenshan 97 A (WA type)	CMS	2014	indica
Yuetai A (ID type)	CMS	2010	indica
You 1A(WA type)	CMS	2011	indica
Zhong 9A (WA type)	CMS	2011	indica
863A (BT type)	CMS	2014	japonica
9201A (BT type)	CMS	2014	japonica
Xu 2A (BT type	CMS	2014	japonica
Nanjing 46A (BT type)	CMS	2014	japonica
731A (BT type)	CMS	2011	japonica
Liuqianxin A (BT type)	CMS	-	japonica
6427A (BT type)	CMS	2010	japonica
Zhendao 88A (BT type)	CMS	2010	japonica
Qingkong A (BT type)	CMS	-	japonica
Yueguang A (BT type)	CMS	-	japonica
Wuqiang A (BT type)	CMS	2014	japonica
Wuyujing 3A (BT type)	CMS	2014	japonica
Liuyan 189A (BT type)	CMS	2014	japonica
Minghui 63	Restorer	2014	indica
Zhenhui 084	Restorer	2014	indica
Yanhui 559	Restorer	2014	indica
Huizi 04	Restorer	2011	indica
Hui 9368	Restorer	2012	indica
Kanghui98	Restorer	2014	indica
C418	Restorer	2014	japonica
Ninghui8hao	Restorer	2014	japonica
Yunhui 4 hao	Restorer	2012	japonica
Zhehui 315	Restorer	2012	japonica
Yanhui R50	Restorer	2012	japonica
Xiushui 04R	Restorer	2011	japonica

Supporting Information Table S2- Total number of constructed SNPLDBs

SNPLDBs	Chr	Allele No	MAF	GD	PIC
1_BLOCK_11884_170248	1	2	0.636	0.463	0.356
S1_334059	1	2	0.667	0.444	0.346
S1_415515	1	2	0.788	0.334	0.278
S1_439337	1	2	0.515	0.500	0.375
S1_439347	1	2	0.697	0.422	0.333
S1_482470	1	2	0.667	0.444	0.346
1_BLOCK_517133_717021	1	4	0.364	0.725	0.676
S1_724562	1	2	0.545	0.496	0.373
1_BLOCK_730836_930115	1	3	0.424	0.643	0.567
1_BLOCK_937039_1124378	1	3	0.485	0.610	0.531
1_BLOCK_1124380_1124381	1	2	0.848	0.257	0.224
S1_1187249	1	2	0.848	0.257	0.224
1_BLOCK_1223377_1363897	1	2	0.758	0.367	0.300
1_BLOCK_1407386_1488564	1	3	0.606	0.544	0.476
1_BLOCK_1492946_1492962	1	2	0.879	0.213	0.190
S1_1500114	1	2	0.515	0.500	0.375
1_BLOCK_1500117_1685379	1	4	0.364	0.720	0.668
1_BLOCK_1701427_1743658	1	2	0.758	0.367	0.300
S1_1849925	1	2	0.636	0.463	0.356
1_BLOCK_1868706_2061536	1	2	0.727	0.397	0.318
1_BLOCK_2068922_2080373	1	2	0.758	0.367	0.300
1_BLOCK_2097624_2283652	1	3	0.394	0.654	0.579
S1_2314234	1	2	0.879	0.213	0.190
S1_2329443	1	2	0.879	0.213	0.190
1_BLOCK_2332370_2531133	1	3	0.455	0.643	0.570
1_BLOCK_2585753_2592129	1	2	0.788	0.334	0.278
1_BLOCK_2592147_2791556	1	3	0.394	0.645	0.569
1_BLOCK_2806713_2852527	1	3	0.394	0.645	0.569
1_BLOCK_2873844_2873879	1	2	0.758	0.367	0.300
1_BLOCK_3005007_3204487	1	2	0.758	0.367	0.300
1_BLOCK_3222912_3415316	1	2	0.697	0.422	0.333
S1_3465932	1	2	0.758	0.367	0.300
S1_3517927	1	2	0.606	0.478	0.363
1_BLOCK_3539052_3655559	1	3	0.424	0.654	0.580
1_BLOCK_3683325_3683332	1	2	0.727	0.397	0.318
1_BLOCK_3698909_3885334	1	3	0.485	0.628	0.556
1_BLOCK_3915958_3915962	1	2	0.576	0.489	0.369

1_BLOCK_3960009_3966347	1	2	0.848	0.257	0.224
1_BLOCK_4102706_4301442	1	2	0.576	0.489	0.369
1_BLOCK_4318172_4318195	1	2	0.576	0.489	0.369
1_BLOCK_4369244_4566243	1	4	0.394	0.720	0.671
1_BLOCK_4587871_4587893	1	2	0.576	0.489	0.369
1_BLOCK_4623234_4818324	1	5	0.303	0.781	0.746
1_BLOCK_4832810_5030523	1	5	0.303	0.781	0.746
1_BLOCK_5046902_5054013	1	2	0.545	0.496	0.373
1_BLOCK_5101277_5275176	1	3	0.424	0.643	0.567
S1_5288678	1	2	0.879	0.213	0.190
S1_5323185	1	2	0.545	0.496	0.373
S1_5365905	1	2	0.576	0.489	0.369
S1_5418151	1	2	0.545	0.496	0.373
1_BLOCK_5458921_5458922	1	2	0.697	0.422	0.333
S1_5461699	1	2	0.758	0.367	0.300
S1_5623790	1	2	0.879	0.213	0.190
S1_5692872	1	2	0.879	0.213	0.190
1_BLOCK_5727265_5727499	1	2	0.667	0.444	0.346
1_BLOCK_5765689_5848535	1	2	0.848	0.257	0.224
1_BLOCK_5944760_6141218	1	3	0.424	0.650	0.576
1_BLOCK_6169868_6222812	1	3	0.515	0.601	0.524
1_BLOCK_6226939_6226968	1	3	0.424	0.650	0.576
1_BLOCK_6393159_6397365	1	2	0.818	0.298	0.253
S1_6397385	1	2	0.879	0.213	0.190
S1_6455482	1	2	0.576	0.489	0.369
1_BLOCK_6462885_6462893	1	2	0.727	0.397	0.318
1_BLOCK_6507307_6507725	1	3	0.455	0.599	0.513
1_BLOCK_6530181_6602271	1	3	0.455	0.599	0.513
S1_6602276	1	2	0.848	0.257	0.224
S1_6623242	1	2	0.879	0.213	0.190
S1_6639394	1	2	0.636	0.463	0.356
S1_6677460	1	2	0.667	0.444	0.346
1_BLOCK_6718664_6913960	1	3	0.485	0.621	0.546
1_BLOCK_6980439_7059479	1	3	0.455	0.637	0.563
S1_7059503	1	2	0.788	0.334	0.278
1_BLOCK_7109068_7293720	1	2	0.515	0.500	0.375
1_BLOCK_7389895_7392478	1	2	0.545	0.496	0.373
1_BLOCK_7393244_7592966	1	2	0.545	0.496	0.373
1_BLOCK_7620047_7685404	1	2	0.545	0.496	0.373
1_BLOCK_7693039_7892056	1	2	0.545	0.496	0.373

1_BLOCK_8851492_8919081	1	2	0.758	0.367	0.300
1_BLOCK_9016350_9020093	1	2	0.727	0.397	0.318
1_BLOCK_9065879_9234139	1	2	0.727	0.397	0.318
1_BLOCK_9340069_9465706	1	2	0.545	0.496	0.373
S1_9617355	1	2	0.545	0.496	0.373
S1_9760374	1	2	0.545	0.496	0.373
S1_9795468	1	2	0.545	0.496	0.373
S1_9859815	1	2	0.545	0.496	0.373
S1_10020093	1	2	0.545	0.496	0.373
1_BLOCK_10048971_10245782	1	3	0.455	0.645	0.572
1_BLOCK_10275866_10473381	1	2	0.545	0.496	0.373
1_BLOCK_10560668_10560684	1	2	0.515	0.500	0.375
S1_10585048	1	2	0.515	0.500	0.375
1_BLOCK_10714307_10904866	1	2	0.545	0.496	0.373
S1_11007976	1	2	0.606	0.478	0.363
S1_11007996	1	2	0.848	0.257	0.224
S1_11008057	1	2	0.606	0.478	0.363
S1_11060760	1	2	0.515	0.500	0.375
S1_11112596	1	2	0.636	0.463	0.356
S1_11356426	1	2	0.848	0.257	0.224
1_BLOCK_11361522_11389150	1	2	0.545	0.496	0.373
1_BLOCK_11409986_11596350	1	2	0.576	0.489	0.369
1_BLOCK_11611673_11697611	1	2	0.576	0.489	0.369
S1_11754203	1	2	0.515	0.500	0.375
S1_11758438	1	2	0.848	0.257	0.224
1_BLOCK_11777088_11858241	1	3	0.485	0.610	0.531
1_BLOCK_12057071_12246081	1	2	0.606	0.478	0.363
S1_12297943	1	2	0.576	0.489	0.369
1_BLOCK_12551516_12745811	1	3	0.485	0.610	0.531
1_BLOCK_12809656_12809690	1	3	0.515	0.588	0.506
S1_12851061	1	2	0.636	0.463	0.356
S1_13266130	1	2	0.848	0.257	0.224
1_BLOCK_13304669_13476237	1	3	0.576	0.571	0.503
1_BLOCK_13539723_13581682	1	3	0.606	0.551	0.488
S1_13713689	1	2	0.879	0.213	0.190
S1_13713704	1	2	0.879	0.213	0.190
S1_13713875	1	2	0.727	0.397	0.318
S1_13713915	1	2	0.909	0.165	0.152
1_BLOCK_13775533_13935420	1	3	0.606	0.551	0.488
1_BLOCK_13957863_14059334	1	3	0.485	0.610	0.531

S1_14356724	1	2	0.515	0.500	0.375
S1_14356750	1	2	0.758	0.367	0.300
S1_14444925	1	2	0.848	0.257	0.224
S1_14460390	1	2	0.515	0.500	0.375
1_BLOCK_14486917_14681592	1	3	0.485	0.621	0.546
1_BLOCK_14747808_14941013	1	4	0.485	0.669	0.618
S1_15133563	1	2	0.758	0.367	0.300
S1_15133565	1	2	0.727	0.397	0.318
1_BLOCK_15187016_15360362	1	4	0.485	0.669	0.618
1_BLOCK_15394539_15440503	1	3	0.485	0.632	0.560
1_BLOCK_15445804_15645099	1	3	0.485	0.610	0.531
1_BLOCK_15673230_15693168	1	2	0.515	0.500	0.375
1_BLOCK_15779628_15959492	1	2	0.515	0.500	0.375
1_BLOCK_16002731_16002965	1	2	0.515	0.500	0.375
S1_16209090	1	2	0.576	0.489	0.369
1_BLOCK_16279911_16458690	1	3	0.485	0.628	0.556
1_BLOCK_16506607_16701982	1	3	0.485	0.632	0.560
S1_16724481	1	2	0.515	0.500	0.375
1_BLOCK_17223957_17230841	1	2	0.515	0.500	0.375
S1_17321830	1	2	0.879	0.213	0.190
1_BLOCK_17482849_17599404	1	2	0.515	0.500	0.375
S1_17798757	1	2	0.879	0.213	0.190
S1_17798758	1	2	0.879	0.213	0.190
1_BLOCK_17836417_18013630	1	4	0.485	0.669	0.618
1_BLOCK_18061249_18259560	1	3	0.485	0.621	0.546
S1_18262731	1	2	0.515	0.500	0.375
S1_18373271	1	2	0.515	0.500	0.375
1_BLOCK_18530428_18721554	1	4	0.364	0.716	0.664
S1_18773911	1	2	0.515	0.500	0.375
S1_18849389	1	2	0.788	0.334	0.278
1_BLOCK_18890784_19090133	1	2	0.515	0.500	0.375
1_BLOCK_19190617_19300355	1	3	0.485	0.628	0.556
1_BLOCK_19378026_19456879	1	2	0.727	0.397	0.318
1_BLOCK_19918470_19960075	1	2	0.788	0.334	0.278
1_BLOCK_20260433_20452579	1	2	0.667	0.444	0.346
1_BLOCK_20527171_20628409	1	2	0.576	0.489	0.369
1_BLOCK_20705678_20904697	1	2	0.576	0.489	0.369
S1_20908374	1	2	0.576	0.489	0.369
S1_20936106	1	2	0.576	0.489	0.369
1_BLOCK_21241063_21385534	1	2	0.515	0.500	0.375

1_BLOCK_21626507_21815113	1	2	0.515	0.500	0.375
1_BLOCK_21898808_21902462	1	2	0.515	0.500	0.375
1_BLOCK_22091630_22138899	1	2	0.515	0.500	0.375
S1_22339396	1	2	0.515	0.500	0.375
S1_22381235	1	2	0.515	0.500	0.375
1_BLOCK_22464975_22639812	1	3	0.485	0.595	0.511
1_BLOCK_22742193_22804420	1	3	0.606	0.544	0.476
S1_22827225	1	2	0.879	0.213	0.190
S1_22881113	1	2	0.879	0.213	0.190
S1_22918491	1	2	0.848	0.257	0.224
1_BLOCK_22923465_23119455	1	3	0.485	0.632	0.560
S1_23168470	1	2	0.879	0.213	0.190
S1_23168483	1	2	0.879	0.213	0.190
1_BLOCK_23171855_23324803	1	5	0.364	0.749	0.710
S1_23348297	1	2	0.788	0.334	0.278
S1_23348329	1	2	0.576	0.489	0.369
S1_23352254	1	2	0.879	0.213	0.190
S1_23353567	1	2	0.909	0.165	0.152
S1_23402698	1	2	0.576	0.489	0.369
1_BLOCK_23426965_23624047	1	4	0.394	0.696	0.641
1_BLOCK_23630930_23633011	1	2	0.606	0.478	0.363
1_BLOCK_23638052_23762074	1	2	0.515	0.500	0.375
1_BLOCK_23921455_23978260	1	3	0.424	0.632	0.554
S1_23979794	1	2	0.606	0.478	0.363
S1_24019091	1	2	0.515	0.500	0.375
S1_24040639	1	2	0.515	0.500	0.375
S1_24064290	1	2	0.515	0.500	0.375
1_BLOCK_24116624_24310319	1	3	0.485	0.628	0.556
1_BLOCK_24440298_24640209	1	2	0.515	0.500	0.375
1_BLOCK_24660525_24849870	1	4	0.364	0.709	0.655
1_BLOCK_24859255_24870572	1	2	0.848	0.257	0.224
1_BLOCK_24870728_25062172	1	3	0.515	0.588	0.506
S1_25077288	1	2	0.515	0.500	0.375
S1_25117984	1	2	0.515	0.500	0.375
1_BLOCK_25250198_25440229	1	3	0.485	0.610	0.531
1_BLOCK_25498956_25508701	1	2	0.515	0.500	0.375
1_BLOCK_25647316_25844706	1	2	0.515	0.500	0.375
1_BLOCK_26042397_26203092	1	2	0.515	0.500	0.375
1_BLOCK_26315815_26438710	1	2	0.515	0.500	0.375
S1_26816312	1	2	0.515	0.500	0.375

1_BLOCK_26822107_27018559	1	2	0.515	0.500	0.375
S1_27051448	1	2	0.576	0.489	0.369
1_BLOCK_27154088_27327868	1	2	0.515	0.500	0.375
1_BLOCK_27506185_27596184	1	2	0.515	0.500	0.375
1_BLOCK_27676677_27876158	1	2	0.515	0.500	0.375
S1_27885486	1	2	0.515	0.500	0.375
1_BLOCK_27907047_28101698	1	3	0.485	0.632	0.560
S1_28116035	1	2	0.727	0.397	0.318
1_BLOCK_28199981_28326846	1	3	0.515	0.601	0.524
1_BLOCK_28355317_28355337	1	2	0.848	0.257	0.224
1_BLOCK_28370747_28569405	1	3	0.485	0.610	0.531
1_BLOCK_28586737_28661358	1	3	0.515	0.601	0.524
1_BLOCK_28709309_28893652	1	2	0.515	0.500	0.375
1_BLOCK_28911006_28919972	1	2	0.606	0.478	0.363
S1_28920012	1	2	0.909	0.165	0.152
1_BLOCK_28934801_29133392	1	2	0.515	0.500	0.375
S1_29137864	1	2	0.515	0.500	0.375
1_BLOCK_29183998_29184004	1	2	0.515	0.500	0.375
S1_29214305	1	2	0.879	0.213	0.190
1_BLOCK_29214308_29214316	1	2	0.576	0.489	0.369
1_BLOCK_29221916_29223416	1	2	0.606	0.478	0.363
1_BLOCK_29271589_29466995	1	2	0.515	0.500	0.375
1_BLOCK_29553367_29624911	1	2	0.515	0.500	0.375
1_BLOCK_29740559_29934926	1	2	0.515	0.500	0.375
1_BLOCK_29944055_29957287	1	2	0.576	0.489	0.369
1_BLOCK_29981228_30179179	1	2	0.576	0.489	0.369
S1_30192832	1	2	0.576	0.489	0.369
1_BLOCK_30231789_30426072	1	2	0.576	0.489	0.369
1_BLOCK_30496453_30590607	1	2	0.576	0.489	0.369
1_BLOCK_30658261_30857925	1	3	0.667	0.496	0.441
S1_30875291	1	2	0.788	0.334	0.278
1_BLOCK_30971251_30974499	1	2	0.576	0.489	0.369
1_BLOCK_30996152_31182054	1	2	0.576	0.489	0.369
S1_31228543	1	2	0.576	0.489	0.369
S1_31253699	1	2	0.576	0.489	0.369
1_BLOCK_31318752_31502481	1	2	0.576	0.489	0.369
S1_31509969	1	2	0.818	0.298	0.253
1_BLOCK_31569243_31621139	1	3	0.576	0.579	0.515
1_BLOCK_31629434_31826554	1	3	0.576	0.562	0.489
S1_31858425	1	2	0.576	0.489	0.369

1_BLOCK_31927823_31944129	1	2	0.576	0.489	0.369
1_BLOCK_31944881_32144745	1	3	0.576	0.562	0.489
S1_32152014	1	2	0.606	0.478	0.363
1_BLOCK_32165536_32357282	1	2	0.606	0.478	0.363
1_BLOCK_32423542_32527981	1	2	0.576	0.489	0.369
S1_32741967	1	2	0.515	0.500	0.375
S1_32769005	1	2	0.879	0.213	0.190
1_BLOCK_32774880_32967259	1	3	0.485	0.595	0.511
1_BLOCK_33081781_33089122	1	2	0.667	0.444	0.346
1_BLOCK_33186006_33384714	1	2	0.727	0.397	0.318
1_BLOCK_33415088_33605143	1	2	0.697	0.422	0.333
S1_33618725	1	2	0.818	0.298	0.253
S1_33618743	1	2	0.697	0.422	0.333
1_BLOCK_33650456_33834110	1	3	0.485	0.628	0.556
S1_33901317	1	2	0.818	0.298	0.253
1_BLOCK_33953368_34151522	1	2	0.697	0.422	0.333
1_BLOCK_34165826_34203951	1	2	0.818	0.298	0.253
S1_34217508	1	2	0.879	0.213	0.190
1_BLOCK_34429295_34456359	1	2	0.727	0.397	0.318
1_BLOCK_34498082_34696663	1	2	0.727	0.397	0.318
S1_34790922	1	2	0.758	0.367	0.300
S1_34814193	1	2	0.727	0.397	0.318
1_BLOCK_34815317_34815318	1	2	0.758	0.367	0.300
1_BLOCK_34834707_35033557	1	2	0.697	0.422	0.333
1_BLOCK_35054667_35054686	1	2	0.697	0.422	0.333
1_BLOCK_35128179_35326649	1	3	0.424	0.654	0.580
1_BLOCK_35377193_35553655	1	2	0.515	0.500	0.375
1_BLOCK_35586111_35781178	1	3	0.485	0.628	0.556
1_BLOCK_35802113_36001449	1	3	0.485	0.628	0.556
S1_36117292	1	2	0.515	0.500	0.375
1_BLOCK_36231865_36231866	1	2	0.515	0.500	0.375
1_BLOCK_36267187_36460968	1	3	0.485	0.628	0.556
1_BLOCK_36474374_36648519	1	3	0.485	0.621	0.546
1_BLOCK_36696208_36791113	1	3	0.485	0.621	0.546
1_BLOCK_36999289_37177949	1	3	0.455	0.643	0.570
1_BLOCK_37273045_37465473	1	2	0.545	0.496	0.373
1_BLOCK_37505826_37533184	1	3	0.545	0.588	0.515
S1_37614990	1	2	0.758	0.367	0.300
1_BLOCK_37797131_37969453	1	3	0.394	0.654	0.579
1_BLOCK_38024481_38223821	1	3	0.394	0.661	0.587

S1_38248562	1	2	0.697	0.422	0.333
1_BLOCK_38279748_38474662	1	3	0.394	0.661	0.587
1_BLOCK_38613250_38656180	1	3	0.394	0.661	0.587
1_BLOCK_38659960_38859936	1	3	0.394	0.661	0.587
1_BLOCK_38859970_38979683	1	2	0.606	0.478	0.363
1_BLOCK_39019028_39218492	1	3	0.455	0.628	0.551
1_BLOCK_39254110_39300436	1	3	0.455	0.628	0.551
1_BLOCK_39449907_39588714	1	3	0.455	0.637	0.563
1_BLOCK_39671900_39828117	1	3	0.455	0.645	0.572
1_BLOCK_39880764_39892588	1	2	0.545	0.496	0.373
1_BLOCK_39902102_40095921	1	2	0.545	0.496	0.373
S1_40102224	1	2	0.545	0.496	0.373
1_BLOCK_40235589_40425980	1	2	0.545	0.496	0.373
S1_40451840	1	2	0.515	0.500	0.375
S1_40478377	1	2	0.909	0.165	0.152
1_BLOCK_40478392_40675807	1	2	0.545	0.496	0.373
1_BLOCK_40696639_40865364	1	2	0.545	0.496	0.373
S1_40923871	1	2	0.788	0.334	0.278
1_BLOCK_40925252_40927344	1	3	0.455	0.637	0.563
1_BLOCK_40928859_41128111	1	3	0.455	0.615	0.535
1_BLOCK_41139415_41184135	1	3	0.455	0.615	0.535
1_BLOCK_41198840_41391130	1	3	0.455	0.615	0.535
S1_41400271	1	2	0.606	0.478	0.363
S1_41553039	1	2	0.606	0.478	0.363
S1_41554577	1	2	0.879	0.213	0.190
1_BLOCK_41563134_41762675	1	4	0.485	0.669	0.618
1_BLOCK_41769016_41839769	1	3	0.485	0.632	0.560
1_BLOCK_41847111_42047109	1	3	0.636	0.522	0.460
1_BLOCK_42047126_42078725	1	3	0.727	0.433	0.393
S1_42081316	1	2	0.848	0.257	0.224
S1_42169777	1	2	0.848	0.257	0.224
1_BLOCK_42325771_42421043	1	2	0.848	0.257	0.224
S1_42460638	1	2	0.818	0.298	0.253
1_BLOCK_42583431_42706910	1	3	0.667	0.500	0.448
S1_42706988	1	2	0.848	0.257	0.224
S1_42745649	1	2	0.848	0.257	0.224
1_BLOCK_42918424_42951223	1	2	0.667	0.444	0.346
1_BLOCK_42959940_43158929	1	2	0.667	0.444	0.346
S1_43165996	1	2	0.879	0.213	0.190
1_BLOCK_43194174_43198111	1	3	0.667	0.500	0.448

S2_14405	2	2	0.697	0.422	0.333
2_BLOCK_22082_221466	2	3	0.576	0.562	0.489
S2_390984	2	2	0.667	0.444	0.346
2_BLOCK_410595_410611	2	2	0.818	0.298	0.253
S2_493711	2	2	0.818	0.298	0.253
S2_493718	2	2	0.909	0.165	0.152
2_BLOCK_670592_670621	2	3	0.576	0.579	0.515
2_BLOCK_889830_1049672	2	2	0.576	0.489	0.369
S2_1360626	2	2	0.727	0.397	0.318
S2_1389486	2	2	0.788	0.334	0.278
2_BLOCK_1391011_1466026	2	2	0.788	0.334	0.278
S2_1486298	2	2	0.909	0.165	0.152
2_BLOCK_2017971_2189673	2	3	0.485	0.621	0.546
S2_2208752	2	2	0.606	0.478	0.363
S2_2426970	2	2	0.606	0.478	0.363
S2_2485001	2	2	0.879	0.213	0.190
S2_2520903	2	2	0.515	0.500	0.375
S2_2659847	2	2	0.606	0.478	0.363
2_BLOCK_2747294_2765422	2	2	0.606	0.478	0.363
S2_2929316	2	2	0.576	0.489	0.369
2_BLOCK_2946315_3108956	2	2	0.515	0.500	0.375
S2_3121156	2	2	0.909	0.165	0.152
S2_3121193	2	2	0.909	0.165	0.152
S2_3224303	2	2	0.788	0.334	0.278
S2_3234815	2	2	0.727	0.397	0.318
2_BLOCK_3369834_3369951	2	2	0.515	0.500	0.375
2_BLOCK_3835627_4032984	2	3	0.424	0.650	0.576
2_BLOCK_4208291_4298713	2	3	0.485	0.610	0.531
S2_4352750	2	2	0.909	0.165	0.152
2_BLOCK_4366758_4559875	2	2	0.515	0.500	0.375
S2_4599352	2	2	0.818	0.298	0.253
2_BLOCK_4625922_4809287	2	3	0.485	0.595	0.511
S2_4852755	2	2	0.818	0.298	0.253
S2_4862895	2	2	0.606	0.478	0.363
2_BLOCK_4937255_4937295	2	2	0.818	0.298	0.253
S2_4954760	2	2	0.576	0.489	0.369
S2_5015094	2	2	0.879	0.213	0.190
2_BLOCK_5045874_5056156	2	2	0.606	0.478	0.363
2_BLOCK_5121184_5306005	2	2	0.576	0.489	0.369
S2_5359418	2	2	0.909	0.165	0.152

2_BLOCK_5366183_5524126	2	2	0.576	0.489	0.369
2_BLOCK_5628437_5749848	2	3	0.545	0.599	0.532
2_BLOCK_5749876_5751131	2	2	0.545	0.496	0.373
S2_5853366	2	2	0.576	0.489	0.369
S2_5857845	2	2	0.576	0.489	0.369
2_BLOCK_5924518_5924521	2	2	0.515	0.500	0.375
S2_5952498	2	2	0.848	0.257	0.224
2_BLOCK_6017400_6217055	2	3	0.455	0.615	0.535
S2_6278645	2	2	0.788	0.334	0.278
2_BLOCK_6433428_6557138	2	3	0.424	0.650	0.576
S2_6557246	2	2	0.848	0.257	0.224
2_BLOCK_6610143_6803100	2	3	0.455	0.615	0.535
S2_6817189	2	2	0.545	0.496	0.373
S2_6826465	2	2	0.848	0.257	0.224
2_BLOCK_6851696_7028091	2	3	0.455	0.643	0.570
2_BLOCK_7064148_7181392	2	3	0.455	0.643	0.570
S2_7249395	2	2	0.788	0.334	0.278
S2_7309688	2	2	0.788	0.334	0.278
S2_7314402	2	2	0.879	0.213	0.190
S2_7373443	2	2	0.545	0.496	0.373
2_BLOCK_7426226_7442397	2	2	0.545	0.496	0.373
S2_7598355	2	2	0.848	0.257	0.224
2_BLOCK_7703686_7745739	2	2	0.545	0.496	0.373
2_BLOCK_7959491_8137774	2	3	0.455	0.645	0.572
2_BLOCK_8229829_8417435	2	3	0.455	0.645	0.572
S2_8491160	2	2	0.727	0.397	0.318
S2_8556426	2	2	0.727	0.397	0.318
S2_8598930	2	2	0.727	0.397	0.318
2_BLOCK_8802031_8875374	2	2	0.879	0.213	0.190
2_BLOCK_8897390_8974112	2	2	0.545	0.496	0.373
2_BLOCK_9258630_9458298	2	3	0.455	0.615	0.535
2_BLOCK_9494517_9554741	2	3	0.455	0.615	0.535
2_BLOCK_9562757_9709356	2	2	0.879	0.213	0.190
S2_9922802	2	2	0.697	0.422	0.333
S2_9922812	2	2	0.758	0.367	0.300
S2_9928053	2	2	0.636	0.463	0.356
2_BLOCK_9943925_10012791	2	2	0.879	0.213	0.190
2_BLOCK_10216097_10318950	2	4	0.455	0.687	0.636
2_BLOCK_10348664_10348667	2	2	0.848	0.257	0.224
2_BLOCK_10397552_10397718	2	2	0.879	0.213	0.190

2_BLOCK_10594879_10594892	2	2	0.667	0.444	0.346
S2_10679900	2	2	0.848	0.257	0.224
S2_10748448	2	2	0.848	0.257	0.224
S2_10922617	2	2	0.545	0.496	0.373
S2_10964183	2	2	0.909	0.165	0.152
2_BLOCK_11102338_11222025	2	2	0.545	0.496	0.373
2_BLOCK_11464922_11642467	2	2	0.545	0.496	0.373
2_BLOCK_11866541_11883552	2	2	0.545	0.496	0.373
S2_11940655	2	2	0.545	0.496	0.373
S2_12086252	2	2	0.545	0.496	0.373
S2_12108044	2	2	0.545	0.496	0.373
S2_12230460	2	2	0.545	0.496	0.373
S2_12427612	2	2	0.545	0.496	0.373
S2_12591523	2	2	0.545	0.496	0.373
2_BLOCK_12695457_12894785	2	2	0.545	0.496	0.373
2_BLOCK_12971138_12972566	2	2	0.545	0.496	0.373
2_BLOCK_13012136_13186014	2	2	0.545	0.496	0.373
2_BLOCK_13305094_13309729	2	2	0.545	0.496	0.373
S2_13873524	2	2	0.545	0.496	0.373
2_BLOCK_14016824_14175859	2	2	0.545	0.496	0.373
2_BLOCK_14218340_14405325	2	2	0.545	0.496	0.373
S2_14436986	2	2	0.576	0.489	0.369
S2_14697540	2	2	0.545	0.496	0.373
S2_14966954	2	2	0.545	0.496	0.373
2_BLOCK_15142124_15161195	2	2	0.545	0.496	0.373
2_BLOCK_15459623_15478598	2	2	0.545	0.496	0.373
2_BLOCK_15663546_15795855	2	2	0.515	0.500	0.375
2_BLOCK_15906351_16033399	2	2	0.515	0.500	0.375
2_BLOCK_16263687_16459984	2	2	0.515	0.500	0.375
S2_16623044	2	2	0.515	0.500	0.375
2_BLOCK_16635759_16835060	2	3	0.515	0.615	0.545
2_BLOCK_16922800_17058740	2	3	0.515	0.617	0.548
2_BLOCK_17150353_17348345	2	3	0.515	0.615	0.545
2_BLOCK_17514570_17514874	2	2	0.758	0.367	0.300
S2_17677364	2	2	0.515	0.500	0.375
2_BLOCK_17804410_17997486	2	2	0.545	0.496	0.373
2_BLOCK_18059754_18237934	2	2	0.515	0.500	0.375
2_BLOCK_18240912_18440248	2	2	0.545	0.496	0.373
2_BLOCK_18525432_18623504	2	2	0.545	0.496	0.373
2_BLOCK_18797073_18809831	2	3	0.485	0.632	0.560

S2_18936972	2	2	0.727	0.397	0.318
S2_19186803	2	2	0.515	0.500	0.375
2_BLOCK_19219304_19230943	2	2	0.515	0.500	0.375
2_BLOCK_19268975_19466710	2	2	0.515	0.500	0.375
2_BLOCK_19497391_19696585	2	3	0.515	0.615	0.545
2_BLOCK_19707004_19897692	2	2	0.515	0.500	0.375
2_BLOCK_19918065_19966979	2	2	0.515	0.500	0.375
2_BLOCK_19975807_20175291	2	3	0.515	0.588	0.506
S2_20179294	2	2	0.636	0.463	0.356
S2_20202588	2	2	0.697	0.422	0.333
S2_20253257	2	2	0.515	0.500	0.375
2_BLOCK_20334035_20530714	2	3	0.485	0.632	0.560
2_BLOCK_20664721_20664731	2	2	0.576	0.489	0.369
2_BLOCK_20690007_20877772	2	2	0.576	0.489	0.369
2_BLOCK_20892714_21085203	2	2	0.606	0.478	0.363
2_BLOCK_21146227_21242681	2	2	0.576	0.489	0.369
2_BLOCK_21328541_21525821	2	2	0.576	0.489	0.369
S2_21534857	2	2	0.576	0.489	0.369
2_BLOCK_21669537_21841015	2	3	0.576	0.562	0.489
2_BLOCK_21905429_22084698	2	3	0.424	0.650	0.576
2_BLOCK_22143883_22260164	2	3	0.545	0.577	0.499
S2_22347808	2	2	0.879	0.213	0.190
S2_22347815	2	2	0.545	0.496	0.373
2_BLOCK_22445145_22445151	2	2	0.515	0.500	0.375
2_BLOCK_22622345_22726627	2	2	0.515	0.500	0.375
2_BLOCK_22844685_23040788	2	2	0.515	0.500	0.375
S2_23143410	2	2	0.515	0.500	0.375
2_BLOCK_23184587_23186386	2	2	0.515	0.500	0.375
2_BLOCK_23246549_23402926	2	3	0.485	0.610	0.531
S2_23456505	2	2	0.515	0.500	0.375
S2_23466199	2	2	0.758	0.367	0.300
S2_23517308	2	2	0.636	0.463	0.356
S2_23552732	2	2	0.727	0.397	0.318
S2_23612045	2	2	0.848	0.257	0.224
S2_23612047	2	2	0.545	0.496	0.373
2_BLOCK_23698653_23890917	2	3	0.576	0.577	0.512
S2_23944127	2	2	0.606	0.478	0.363
S2_23957987	2	2	0.788	0.334	0.278
S2_24022828	2	2	0.758	0.367	0.300
2_BLOCK_24022845_24128845	2	4	0.303	0.742	0.694

S2_24136482	2	2	0.758	0.367	0.300
S2_24165883	2	2	0.606	0.478	0.363
S2_24221175	2	2	0.697	0.422	0.333
S2_24229224	2	2	0.758	0.367	0.300
S2_24278307	2	2	0.606	0.478	0.363
S2_24278397	2	2	0.758	0.367	0.300
S2_24283132	2	2	0.758	0.367	0.300
S2_24490847	2	2	0.848	0.257	0.224
S2_24498581	2	2	0.909	0.165	0.152
S2_24518628	2	2	0.848	0.257	0.224
2_BLOCK_24571861_24661819	2	2	0.879	0.213	0.190
S2_24668067	2	2	0.636	0.463	0.356
2_BLOCK_24680739_24680760	2	2	0.879	0.213	0.190
2_BLOCK_24680792_24752084	2	5	0.273	0.792	0.758
2_BLOCK_24820999_25013169	2	4	0.303	0.742	0.694
S2_25053102	2	2	0.606	0.478	0.363
2_BLOCK_25142102_25304942	2	3	0.455	0.628	0.551
2_BLOCK_25359391_25409193	2	2	0.515	0.500	0.375
S2_25445683	2	2	0.818	0.298	0.253
2_BLOCK_25460419_25653044	2	3	0.485	0.621	0.546
2_BLOCK_25684089_25871868	2	3	0.485	0.632	0.560
S2_25983298	2	2	0.545	0.496	0.373
2_BLOCK_26014682_26204848	2	2	0.515	0.500	0.375
S2_26247587	2	2	0.515	0.500	0.375
2_BLOCK_26289582_26453626	2	2	0.515	0.500	0.375
S2_26453706	2	2	0.848	0.257	0.224
2_BLOCK_26457004_26555485	2	2	0.515	0.500	0.375
2_BLOCK_26619058_26817007	2	2	0.515	0.500	0.375
2_BLOCK_26960912_27158256	2	2	0.515	0.500	0.375
2_BLOCK_27249669_27436851	2	2	0.515	0.500	0.375
2_BLOCK_27553845_27572325	2	2	0.515	0.500	0.375
2_BLOCK_27614723_27807851	2	2	0.515	0.500	0.375
S2_27915617	2	2	0.515	0.500	0.375
S2_27993957	2	2	0.515	0.500	0.375
2_BLOCK_28014459_28212508	2	2	0.515	0.500	0.375
2_BLOCK_28219934_28239237	2	2	0.515	0.500	0.375
S2_28267320	2	2	0.515	0.500	0.375
2_BLOCK_28350658_28546577	2	2	0.515	0.500	0.375
S2_28676972	2	2	0.515	0.500	0.375
S2_28789104	2	2	0.606	0.478	0.363

2_BLOCK_28831793_29020268	2	2	0.515	0.500	0.375
2_BLOCK_29035142_29227846	2	4	0.485	0.661	0.608
S2_29319307	2	2	0.667	0.444	0.346
S2_29330362	2	2	0.848	0.257	0.224
S2_29330952	2	2	0.515	0.500	0.375
2_BLOCK_29406549_29599982	2	2	0.515	0.500	0.375
S2_29609136	2	2	0.879	0.213	0.190
2_BLOCK_29637041_29637074	2	2	0.545	0.496	0.373
S2_29704169	2	2	0.515	0.500	0.375
2_BLOCK_29709333_29907333	2	2	0.515	0.500	0.375
2_BLOCK_29922689_30110079	2	2	0.515	0.500	0.375
2_BLOCK_30132327_30262343	2	3	0.515	0.601	0.524
2_BLOCK_30353435_30538317	2	2	0.515	0.500	0.375
2_BLOCK_30563440_30605618	2	3	0.545	0.588	0.515
2_BLOCK_30605636_30605660	2	2	0.667	0.444	0.346
2_BLOCK_30606460_30806327	2	3	0.485	0.621	0.546
S2_30815684	2	2	0.818	0.298	0.253
S2_30874372	2	2	0.818	0.298	0.253
2_BLOCK_30883632_31082829	2	3	0.485	0.628	0.556
2_BLOCK_31105614_31105624	2	2	0.515	0.500	0.375
2_BLOCK_31153479_31317170	2	2	0.515	0.500	0.375
2_BLOCK_31322102_31521245	2	2	0.515	0.500	0.375
S2_31544281	2	2	0.848	0.257	0.224
2_BLOCK_31570624_31725636	2	2	0.515	0.500	0.375
S2_31770057	2	2	0.788	0.334	0.278
2_BLOCK_31784170_31979982	2	2	0.515	0.500	0.375
2_BLOCK_32064193_32092772	2	2	0.515	0.500	0.375
2_BLOCK_32092807_32292788	2	2	0.515	0.500	0.375
2_BLOCK_32352294_32401348	2	3	0.485	0.632	0.560
2_BLOCK_32441761_32638373	2	2	0.515	0.500	0.375
2_BLOCK_32747385_32887271	2	2	0.515	0.500	0.375
S2_32911847	2	2	0.879	0.213	0.190
2_BLOCK_32991954_33190958	2	3	0.515	0.610	0.537
S2_33199545	2	2	0.515	0.500	0.375
S2_33246200	2	2	0.515	0.500	0.375
S2_33250796	2	2	0.758	0.367	0.300
2_BLOCK_33373078_33571449	2	2	0.515	0.500	0.375
S2_33596445	2	2	0.727	0.397	0.318
S2_33621612	2	2	0.515	0.500	0.375
2_BLOCK_33706123_33888029	2	2	0.515	0.500	0.375

2_BLOCK_33926825_33946459	2	2	0.545	0.496	0.373
S2_34000118	2	2	0.515	0.500	0.375
S2_34039452	2	2	0.879	0.213	0.190
2_BLOCK_34232277_34396486	2	4	0.455	0.687	0.636
S2_34408443	2	2	0.788	0.334	0.278
S2_34509829	2	2	0.545	0.496	0.373
S2_34578499	2	2	0.788	0.334	0.278
2_BLOCK_34586525_34769687	2	2	0.576	0.489	0.369
2_BLOCK_34796411_34798618	2	2	0.545	0.496	0.373
2_BLOCK_34802345_34921078	2	4	0.364	0.720	0.669
S2_34978568	2	2	0.848	0.257	0.224
2_BLOCK_34978639_35048180	2	3	0.485	0.610	0.531
2_BLOCK_35053482_35242550	2	4	0.333	0.722	0.671
S2_35261285	2	2	0.667	0.444	0.346
2_BLOCK_35263538_35308304	2	2	0.636	0.463	0.356
2_BLOCK_35329481_35527065	2	2	0.545	0.496	0.373
2_BLOCK_35543821_35573882	2	3	0.485	0.595	0.511
2_BLOCK_35591725_35790248	2	3	0.394	0.645	0.569
2_BLOCK_35817740_35924060	2	5	0.242	0.790	0.756
2_BLOCK_35924149_35924153	2	2	0.758	0.367	0.300
S3_249605	3	2	0.545	0.496	0.373
S3_363175	3	2	0.879	0.213	0.190
S3_413990	3	2	0.818	0.298	0.253
3_BLOCK_456769_656337	3	2	0.515	0.500	0.375
3_BLOCK_703621_760723	3	2	0.515	0.500	0.375
3_BLOCK_762653_961200	3	2	0.515	0.500	0.375
3_BLOCK_1010229_1026712	3	2	0.515	0.500	0.375
S3_1042153	3	2	0.515	0.500	0.375
3_BLOCK_1166581_1359657	3	2	0.515	0.500	0.375
3_BLOCK_1379149_1476389	3	2	0.515	0.500	0.375
3_BLOCK_1486602_1685407	3	3	0.485	0.595	0.511
S3_1693960	3	2	0.697	0.422	0.333
3_BLOCK_1705565_1762714	3	3	0.515	0.601	0.524
S3_1775268	3	2	0.667	0.444	0.346
3_BLOCK_1781402_1887875	3	3	0.515	0.601	0.524
S3_2052208	3	2	0.515	0.500	0.375
S3_2084667	3	2	0.515	0.500	0.375
S3_2118803	3	2	0.879	0.213	0.190
S3_2142571	3	2	0.848	0.257	0.224
3_BLOCK_2153195_2350558	3	4	0.485	0.669	0.618

S3_2390481	3	2	0.879	0.213	0.190
3_BLOCK_2507338_2683292	3	3	0.485	0.595	0.511
3_BLOCK_2736967_2935082	3	3	0.485	0.595	0.511
3_BLOCK_2954955_3151841	3	3	0.485	0.595	0.511
S3_3157232	3	2	0.515	0.500	0.375
3_BLOCK_3260150_3445001	3	3	0.485	0.610	0.531
S3_3469376	3	2	0.545	0.496	0.373
3_BLOCK_3534897_3729386	3	2	0.515	0.500	0.375
3_BLOCK_3778574_3976290	3	2	0.515	0.500	0.375
S3_3999625	3	2	0.515	0.500	0.375
3_BLOCK_4193314_4390073	3	2	0.545	0.496	0.373
S3_4539636	3	2	0.515	0.500	0.375
S3_4568976	3	2	0.515	0.500	0.375
S3_4591991	3	2	0.515	0.500	0.375
3_BLOCK_4614468_4806602	3	3	0.515	0.588	0.506
S3_4822428	3	2	0.848	0.257	0.224
3_BLOCK_4823893_4924144	3	3	0.515	0.601	0.524
3_BLOCK_4944877_5143310	3	3	0.485	0.595	0.511
S3_5182383	3	2	0.515	0.500	0.375
3_BLOCK_5211057_5411035	3	2	0.515	0.500	0.375
3_BLOCK_5451085_5620883	3	2	0.515	0.500	0.375
3_BLOCK_5669375_5869175	3	5	0.364	0.757	0.719
3_BLOCK_5870918_6048391	3	3	0.485	0.610	0.531
3_BLOCK_6060874_6252981	3	2	0.545	0.496	0.373
3_BLOCK_6279424_6404635	3	2	0.848	0.257	0.224
S3_6487939	3	2	0.545	0.496	0.373
3_BLOCK_6520556_6531124	3	2	0.545	0.496	0.373
3_BLOCK_6625585_6812441	3	3	0.455	0.615	0.535
3_BLOCK_6863874_6870558	3	2	0.515	0.500	0.375
S3_6948281	3	2	0.848	0.257	0.224
3_BLOCK_6981083_7067292	3	2	0.545	0.496	0.373
3_BLOCK_7126113_7318531	3	2	0.545	0.496	0.373
3_BLOCK_7318602_7518184	3	3	0.545	0.595	0.526
3_BLOCK_7558969_7670531	3	3	0.545	0.595	0.526
3_BLOCK_7670590_7836961	3	4	0.364	0.720	0.669
3_BLOCK_7917856_7946237	3	3	0.545	0.595	0.526
3_BLOCK_7966099_8157634	3	3	0.545	0.595	0.526
S3_8169815	3	2	0.606	0.478	0.363
3_BLOCK_8174321_8372262	3	3	0.455	0.615	0.535
S3_8418438	3	2	0.848	0.257	0.224

S3_8418485	3	2	0.606	0.478	0.363
S3_8434143	3	2	0.848	0.257	0.224
S3_8434151	3	2	0.606	0.478	0.363
S3_8547926	3	2	0.879	0.213	0.190
S3_8600006	3	2	0.848	0.257	0.224
S3_8624968	3	2	0.727	0.397	0.318
S3_8633455	3	2	0.848	0.257	0.224
S3_8648123	3	2	0.848	0.257	0.224
S3_8648429	3	2	0.545	0.496	0.373
S3_8702596	3	2	0.606	0.478	0.363
S3_8727351	3	2	0.848	0.257	0.224
S3_8727847	3	2	0.545	0.496	0.373
3_BLOCK_8727874_8811527	3	5	0.303	0.775	0.739
3_BLOCK_8823816_9020873	3	4	0.394	0.705	0.652
3_BLOCK_9069054_9212249	3	3	0.697	0.466	0.419
3_BLOCK_9356300_9359704	3	2	0.727	0.397	0.318
S3_9363022	3	2	0.727	0.397	0.318
3_BLOCK_9499433_9499513	3	2	0.636	0.463	0.356
3_BLOCK_9599101_9781950	3	2	0.848	0.257	0.224
3_BLOCK_9849247_9849252	3	3	0.455	0.599	0.513
S3_9896089	3	2	0.879	0.213	0.190
S3_9904884	3	2	0.545	0.496	0.373
3_BLOCK_9933834_10133555	3	6	0.242	0.819	0.794
3_BLOCK_10154683_10238722	3	2	0.545	0.496	0.373
S3_10250985	3	2	0.848	0.257	0.224
3_BLOCK_10276261_10474420	3	4	0.455	0.687	0.636
3_BLOCK_10477564_10651116	3	3	0.455	0.599	0.513
3_BLOCK_10657758_10857422	3	3	0.455	0.599	0.513
3_BLOCK_10987991_10990697	3	3	0.455	0.628	0.551
3_BLOCK_10990764_10990765	3	2	0.758	0.367	0.300
3_BLOCK_10991463_11168887	3	4	0.455	0.687	0.636
S3_11175823	3	2	0.879	0.213	0.190
S3_11192839	3	2	0.758	0.367	0.300
3_BLOCK_11308672_11377417	3	3	0.455	0.637	0.563
3_BLOCK_11490401_11673944	3	3	0.455	0.599	0.513
3_BLOCK_11713630_11750248	3	3	0.455	0.637	0.563
3_BLOCK_11820299_12019164	3	2	0.545	0.496	0.373
3_BLOCK_12027336_12152139	3	2	0.545	0.496	0.373
S3_12263361	3	2	0.636	0.463	0.356
3_BLOCK_12372675_12539571	3	4	0.455	0.681	0.629

S3_12717832	3	2	0.667	0.444	0.346
3_BLOCK_12760850_12946497	3	3	0.545	0.588	0.515
S3_13108834	3	2	0.818	0.298	0.253
3_BLOCK_13313026_13313029	3	2	0.667	0.444	0.346
S3_13431721	3	2	0.545	0.496	0.373
S3_13587616	3	2	0.697	0.422	0.333
S3_13605241	3	2	0.545	0.496	0.373
S3_13670246	3	2	0.727	0.397	0.318
S3_13670247	3	2	0.788	0.334	0.278
3_BLOCK_13838004_13996582	3	5	0.333	0.770	0.733
S3_13996583	3	2	0.727	0.397	0.318
S3_14000378	3	2	0.848	0.257	0.224
S3_14017252	3	2	0.879	0.213	0.190
3_BLOCK_14017261_14096569	3	3	0.455	0.599	0.513
3_BLOCK_14144685_14343272	3	3	0.455	0.615	0.535
3_BLOCK_14358468_14501870	3	4	0.394	0.696	0.641
3_BLOCK_14543264_14543295	3	2	0.606	0.478	0.363
3_BLOCK_14595689_14595704	3	2	0.515	0.500	0.375
3_BLOCK_14595705_14789758	3	3	0.515	0.615	0.545
S3_14813816	3	2	0.848	0.257	0.224
S3_14847139	3	2	0.848	0.257	0.224
S3_14872395	3	2	0.848	0.257	0.224
S3_14929985	3	2	0.848	0.257	0.224
S3_14935019	3	2	0.545	0.496	0.373
S3_15001137	3	2	0.667	0.444	0.346
S3_15001183	3	2	0.545	0.496	0.373
3_BLOCK_15011318_15203194	3	3	0.455	0.637	0.563
3_BLOCK_15214594_15332976	3	2	0.515	0.500	0.375
3_BLOCK_15377322_15573013	3	3	0.485	0.628	0.556
3_BLOCK_15618420_15790368	3	2	0.515	0.500	0.375
3_BLOCK_15825442_15826203	3	2	0.515	0.500	0.375
3_BLOCK_15864307_16039430	3	3	0.545	0.588	0.515
3_BLOCK_16066501_16089971	3	2	0.515	0.500	0.375
S3_16188838	3	2	0.848	0.257	0.224
3_BLOCK_16296298_16383958	3	2	0.758	0.367	0.300
3_BLOCK_16399707_16576029	3	3	0.424	0.654	0.580
S3_16645599	3	2	0.758	0.367	0.300
S3_16665988	3	2	0.879	0.213	0.190
S3_16707808	3	2	0.879	0.213	0.190
S3_16749068	3	2	0.758	0.367	0.300

3_BLOCK_16777275_16971984	3	2	0.788	0.334	0.278
3_BLOCK_17054281_17143926	3	2	0.545	0.496	0.373
3_BLOCK_17277287_17471750	3	2	0.545	0.496	0.373
3_BLOCK_17531508_17719230	3	3	0.515	0.588	0.506
3_BLOCK_17824913_17939572	3	3	0.485	0.610	0.531
S3_18181316	3	2	0.515	0.500	0.375
S3_18277614	3	2	0.697	0.422	0.333
S3_18410408	3	2	0.515	0.500	0.375
S3_18443765	3	2	0.848	0.257	0.224
S3_18564453	3	2	0.515	0.500	0.375
S3_19405589	3	2	0.515	0.500	0.375
S3_19930029	3	2	0.515	0.500	0.375
3_BLOCK_20012717_20012748	3	2	0.515	0.500	0.375
3_BLOCK_20032832_20216158	3	2	0.515	0.500	0.375
S3_20311999	3	2	0.515	0.500	0.375
S3_20369153	3	2	0.515	0.500	0.375
3_BLOCK_20580960_20778904	3	2	0.515	0.500	0.375
3_BLOCK_20869170_21056201	3	2	0.515	0.500	0.375
S3_21105409	3	2	0.727	0.397	0.318
S3_21123765	3	2	0.848	0.257	0.224
S3_21151152	3	2	0.758	0.367	0.300
S3_21203413	3	2	0.848	0.257	0.224
S3_21272710	3	2	0.515	0.500	0.375
3_BLOCK_21309394_21474197	3	3	0.485	0.621	0.546
S3_21635957	3	2	0.848	0.257	0.224
S3_21642315	3	2	0.818	0.298	0.253
S3_21642331	3	2	0.667	0.444	0.346
S3_21659472	3	2	0.788	0.334	0.278
S3_21660068	3	2	0.515	0.500	0.375
S3_21660079	3	2	0.818	0.298	0.253
S3_21667634	3	2	0.818	0.298	0.253
S3_21667646	3	2	0.515	0.500	0.375
3_BLOCK_21702272_21708917	3	2	0.848	0.257	0.224
S3_21710261	3	2	0.818	0.298	0.253
S3_21710263	3	2	0.879	0.213	0.190
S3_21758874	3	2	0.818	0.298	0.253
S3_22008164	3	2	0.697	0.422	0.333
S3_22010491	3	2	0.848	0.257	0.224
S3_22010502	3	2	0.515	0.500	0.375
S3_22029186	3	2	0.788	0.334	0.278

S3_22049672	3	2	0.848	0.257	0.224
3_BLOCK_22205955_22376503	3	4	0.485	0.676	0.629
3_BLOCK_22435236_22599561	3	4	0.485	0.669	0.618
3_BLOCK_22599562_22743505	3	3	0.455	0.645	0.572
3_BLOCK_22784145_22817514	3	4	0.455	0.689	0.639
3_BLOCK_22823088_22823134	3	2	0.545	0.496	0.373
3_BLOCK_22846153_23033364	3	3	0.455	0.615	0.535
S3_23053168	3	2	0.758	0.367	0.300
S3_23125034	3	2	0.879	0.213	0.190
3_BLOCK_23155569_23343573	3	3	0.455	0.599	0.513
S3_23356298	3	2	0.848	0.257	0.224
S3_23390353	3	2	0.545	0.496	0.373
3_BLOCK_23446209_23644731	3	3	0.455	0.599	0.513
3_BLOCK_23655154_23699609	3	2	0.545	0.496	0.373
3_BLOCK_23699614_23699620	3	2	0.879	0.213	0.190
3_BLOCK_23715656_23911358	3	4	0.455	0.687	0.636
S3_23985565	3	2	0.848	0.257	0.224
3_BLOCK_24050572_24242511	3	2	0.545	0.496	0.373
S3_24273011	3	2	0.545	0.496	0.373
3_BLOCK_24352224_24546847	3	3	0.515	0.588	0.506
S3_24579105	3	2	0.606	0.478	0.363
S3_24807009	3	2	0.879	0.213	0.190
S3_24866032	3	2	0.879	0.213	0.190
S3_24910805	3	2	0.879	0.213	0.190
S3_25096347	3	2	0.879	0.213	0.190
S3_25096634	3	2	0.515	0.500	0.375
S3_25140903	3	2	0.515	0.500	0.375
3_BLOCK_25310266_25482341	3	2	0.667	0.444	0.346
S3_25530777	3	2	0.727	0.397	0.318
S3_25733777	3	2	0.909	0.165	0.152
S3_25966710	3	2	0.848	0.257	0.224
3_BLOCK_26340981_26349267	3	2	0.848	0.257	0.224
S3_26365674	3	2	0.758	0.367	0.300
S3_26400599	3	2	0.758	0.367	0.300
3_BLOCK_26408270_26542118	3	3	0.636	0.522	0.460
S3_26546464	3	2	0.576	0.489	0.369
3_BLOCK_26576819_26597826	3	2	0.879	0.213	0.190
3_BLOCK_26597888_26785589	3	3	0.727	0.433	0.393
S3_26785635	3	2	0.818	0.298	0.253
S3_26845909	3	2	0.545	0.496	0.373

S3_26869445	3	2	0.515	0.500	0.375
S3_26923364	3	2	0.545	0.496	0.373
S3_27052904	3	2	0.879	0.213	0.190
3_BLOCK_27113689_27170198	3	2	0.545	0.496	0.373
3_BLOCK_27409078_27469504	3	2	0.545	0.496	0.373
S3_27520616	3	2	0.848	0.257	0.224
3_BLOCK_27655388_27851081	3	3	0.455	0.643	0.570
S3_27853983	3	2	0.606	0.478	0.363
3_BLOCK_27919300_28111909	3	2	0.545	0.496	0.373
3_BLOCK_28141092_28144050	3	3	0.545	0.595	0.526
3_BLOCK_28179401_28361061	3	3	0.455	0.628	0.551
3_BLOCK_28390337_28569033	3	3	0.545	0.577	0.499
S3_29019589	3	2	0.545	0.496	0.373
3_BLOCK_29154646_29305037	3	3	0.455	0.628	0.551
S3_29372257	3	2	0.879	0.213	0.190
S3_29402638	3	2	0.879	0.213	0.190
S3_29414614	3	2	0.879	0.213	0.190
3_BLOCK_29450021_29644550	3	4	0.455	0.689	0.639
3_BLOCK_29678724_29845947	3	3	0.455	0.643	0.570
S3_29896880	3	2	0.545	0.496	0.373
3_BLOCK_30004678_30203977	3	3	0.545	0.599	0.532
3_BLOCK_30261516_30267593	3	2	0.515	0.500	0.375
3_BLOCK_30317556_30516863	3	2	0.545	0.496	0.373
3_BLOCK_30523036_30523640	3	2	0.636	0.463	0.356
S3_30561228	3	2	0.545	0.496	0.373
S3_30607373	3	2	0.788	0.334	0.278
3_BLOCK_30724062_30724163	3	2	0.545	0.496	0.373
S3_30794834	3	2	0.727	0.397	0.318
S3_30843379	3	2	0.909	0.165	0.152
3_BLOCK_30843380_31033330	3	3	0.455	0.628	0.551
3_BLOCK_31065007_31085398	3	2	0.727	0.397	0.318
3_BLOCK_31148692_31329397	3	2	0.727	0.397	0.318
3_BLOCK_31372721_31437173	3	2	0.727	0.397	0.318
3_BLOCK_31441454_31528278	3	2	0.636	0.463	0.356
3_BLOCK_31565334_31754259	3	2	0.636	0.463	0.356
S3_31789099	3	2	0.636	0.463	0.356
3_BLOCK_31803423_32002941	3	4	0.333	0.725	0.674
3_BLOCK_32063213_32072052	3	2	0.545	0.496	0.373
3_BLOCK_32094919_32293169	3	2	0.545	0.496	0.373
3_BLOCK_32454923_32617863	3	2	0.545	0.496	0.373

S3_32734935	3	2	0.758	0.367	0.300
3_BLOCK_32744450_32923516	3	3	0.455	0.643	0.570
3_BLOCK_32969173_33157926	3	3	0.455	0.599	0.513
3_BLOCK_33174334_33302562	3	2	0.545	0.496	0.373
3_BLOCK_33314473_33512006	3	2	0.545	0.496	0.373
3_BLOCK_33811752_34004909	3	3	0.455	0.643	0.570
3_BLOCK_34011869_34194446	3	3	0.485	0.628	0.556
3_BLOCK_34231621_34431183	3	2	0.545	0.496	0.373
S3_34431673	3	2	0.545	0.496	0.373
3_BLOCK_34491593_34690605	3	2	0.545	0.496	0.373
3_BLOCK_34786863_34881796	3	3	0.485	0.632	0.560
S3_35146504	3	2	0.697	0.422	0.333
3_BLOCK_35183949_35197258	3	2	0.818	0.298	0.253
3_BLOCK_35212799_35266337	3	3	0.545	0.595	0.526
S3_35310534	3	2	0.788	0.334	0.278
3_BLOCK_35321311_35397952	3	3	0.455	0.645	0.572
S3_35562856	3	2	0.697	0.422	0.333
S3_35607287	3	2	0.545	0.496	0.373
S3_35611464	3	2	0.667	0.444	0.346
S3_35771770	3	2	0.515	0.500	0.375
3_BLOCK_35818153_35826272	3	3	0.455	0.628	0.551
3_BLOCK_35870708_36064855	3	3	0.455	0.615	0.535
S3_36064887	3	2	0.606	0.478	0.363
3_BLOCK_36189479_36189483	3	2	0.576	0.489	0.369
S3_36189485	3	2	0.879	0.213	0.190
S3_36189489	3	2	0.879	0.213	0.190
S3_36189496	3	2	0.576	0.489	0.369
S3_36284349	3	2	0.818	0.298	0.253
S3_36293343	3	2	0.515	0.500	0.375
S3_36360798	3	2	0.515	0.500	0.375
4_BLOCK_60991_204460	4	2	0.545	0.496	0.373
4_BLOCK_222357_411888	4	5	0.333	0.762	0.724
S4_413151	4	2	0.848	0.257	0.224
S4_414092	4	2	0.727	0.397	0.318
S4_438075	4	2	0.515	0.500	0.375
4_BLOCK_487343_487344	4	2	0.727	0.397	0.318
S4_488385	4	2	0.818	0.298	0.253
4_BLOCK_488394_559007	4	4	0.424	0.702	0.651
4_BLOCK_598877_604980	4	3	0.424	0.650	0.576
S4_604997	4	2	0.909	0.165	0.152

S4_795206	4	2	0.879	0.213	0.190
4_BLOCK_968563_1000320	4	2	0.606	0.478	0.363
S4_1019414	4	2	0.818	0.298	0.253
4_BLOCK_1025155_1188932	4	4	0.424	0.702	0.651
4_BLOCK_1234039_1248580	4	2	0.576	0.489	0.369
S4_1647249	4	2	0.697	0.422	0.333
4_BLOCK_1647264_1717761	4	4	0.485	0.661	0.608
4_BLOCK_1743726_1755810	4	3	0.455	0.637	0.563
4_BLOCK_1755817_1792720	4	3	0.515	0.615	0.545
4_BLOCK_2048257_2247084	4	3	0.455	0.599	0.513
4_BLOCK_2282119_2282140	4	2	0.576	0.489	0.369
4_BLOCK_2463662_2521608	4	4	0.515	0.654	0.607
S4_2890194	4	2	0.879	0.213	0.190
S4_2909020	4	2	0.515	0.500	0.375
S4_3108372	4	2	0.636	0.463	0.356
4_BLOCK_3250324_3426279	4	3	0.394	0.645	0.569
4_BLOCK_3531738_3531749	4	2	0.515	0.500	0.375
4_BLOCK_3611236_3657051	4	3	0.727	0.433	0.393
S4_3657061	4	2	0.879	0.213	0.190
S4_3785376	4	2	0.576	0.489	0.369
4_BLOCK_4319282_4319325	4	2	0.515	0.500	0.375
4_BLOCK_4320751_4442555	4	2	0.545	0.496	0.373
S4_4513315	4	2	0.879	0.213	0.190
S4_4513754	4	2	0.545	0.496	0.373
S4_4732607	4	2	0.758	0.367	0.300
S4_4739773	4	2	0.879	0.213	0.190
4_BLOCK_4739845_4739865	4	2	0.848	0.257	0.224
S4_4764096	4	2	0.515	0.500	0.375
4_BLOCK_4800096_4997120	4	3	0.515	0.601	0.524
4_BLOCK_5005782_5135455	4	4	0.333	0.729	0.679
S4_5147518	4	2	0.727	0.397	0.318
S4_5162078	4	2	0.515	0.500	0.375
4_BLOCK_5190213_5379435	4	4	0.364	0.725	0.676
S4_5484113	4	2	0.636	0.463	0.356
S4_5598742	4	2	0.545	0.496	0.373
S4_5649373	4	2	0.576	0.489	0.369
S4_5750215	4	2	0.727	0.397	0.318
S4_5750223	4	2	0.818	0.298	0.253
4_BLOCK_5806676_5996478	4	4	0.424	0.707	0.658
S4_6012337	4	2	0.606	0.478	0.363

S4_6012378	4	2	0.636	0.463	0.356
4_BLOCK_6228069_6243812	4	2	0.848	0.257	0.224
4_BLOCK_6255427_6455225	4	4	0.485	0.669	0.618
S4_6478912	4	2	0.515	0.500	0.375
S4_6638393	4	2	0.545	0.496	0.373
4_BLOCK_6659872_6659923	4	2	0.758	0.367	0.300
4_BLOCK_6925085_6925097	4	3	0.515	0.588	0.506
4_BLOCK_6937764_6939123	4	2	0.818	0.298	0.253
4_BLOCK_6939158_6968293	4	4	0.333	0.738	0.690
4_BLOCK_7311486_7318178	4	2	0.515	0.500	0.375
4_BLOCK_8040480_8064912	4	3	0.485	0.632	0.560
4_BLOCK_8303935_8485602	4	3	0.485	0.632	0.560
4_BLOCK_8506139_8518859	4	2	0.515	0.500	0.375
4_BLOCK_8771842_8774333	4	2	0.515	0.500	0.375
S4_8814555	4	2	0.788	0.334	0.278
S4_9076629	4	2	0.515	0.500	0.375
S4_9406014	4	2	0.515	0.500	0.375
S4_9891869	4	2	0.515	0.500	0.375
S4_9941614	4	2	0.515	0.500	0.375
4_BLOCK_10748099_10862434	4	2	0.515	0.500	0.375
4_BLOCK_11062780_11181446	4	3	0.485	0.632	0.560
4_BLOCK_11308986_11466373	4	4	0.485	0.669	0.618
4_BLOCK_11573916_11577326	4	3	0.485	0.632	0.560
4_BLOCK_11589351_11779886	4	3	0.485	0.632	0.560
S4_11803874	4	2	0.727	0.397	0.318
4_BLOCK_11861449_12047086	4	2	0.515	0.500	0.375
4_BLOCK_12396204_12591679	4	2	0.515	0.500	0.375
4_BLOCK_12641745_12647142	4	3	0.485	0.621	0.546
4_BLOCK_12725027_12725071	4	2	0.879	0.213	0.190
4_BLOCK_12957681_12957732	4	2	0.515	0.500	0.375
S4_13000650	4	2	0.879	0.213	0.190
4_BLOCK_13011200_13011205	4	2	0.697	0.422	0.333
S4_13217448	4	2	0.515	0.500	0.375
4_BLOCK_13523642_13523665	4	3	0.636	0.527	0.470
4_BLOCK_13619097_13619099	4	2	0.515	0.500	0.375
S4_13818469	4	2	0.848	0.257	0.224
4_BLOCK_14047340_14210685	4	3	0.606	0.551	0.488
4_BLOCK_14819450_14819459	4	2	0.667	0.444	0.346
S4_15886900	4	2	0.606	0.478	0.363
S4_16197065	4	2	0.879	0.213	0.190

S4_16441661	4	2	0.848	0.257	0.224
4_BLOCK_16530787_16547487	4	3	0.485	0.628	0.556
4_BLOCK_16672214_16709477	4	2	0.667	0.444	0.346
4_BLOCK_17240304_17260478	4	2	0.788	0.334	0.278
4_BLOCK_17319905_17493945	4	2	0.788	0.334	0.278
S4_17570220	4	2	0.909	0.165	0.152
4_BLOCK_17584975_17719708	4	2	0.788	0.334	0.278
4_BLOCK_17882078_17907416	4	1	1.000	0.000	0.000
4_BLOCK_17908884_17913705	4	2	0.788	0.334	0.278
4_BLOCK_17938400_18109397	4	3	0.394	0.661	0.587
4_BLOCK_18109398_18109400	4	2	0.545	0.496	0.373
4_BLOCK_18142835_18154552	4	2	0.848	0.257	0.224
4_BLOCK_18213262_18397094	4	2	0.848	0.257	0.224
S4_18420498	4	2	0.848	0.257	0.224
4_BLOCK_18786762_18877792	4	2	0.848	0.257	0.224
S4_19002084	4	2	0.758	0.367	0.300
S4_19135690	4	2	0.727	0.397	0.318
S4_19238998	4	2	0.697	0.422	0.333
4_BLOCK_19245131_19322232	4	2	0.727	0.397	0.318
S4_19530013	4	2	0.697	0.422	0.333
4_BLOCK_19576610_19769633	4	3	0.515	0.588	0.506
4_BLOCK_20025003_20066098	4	2	0.606	0.478	0.363
S4_20319162	4	2	0.515	0.500	0.375
4_BLOCK_20349163_20547988	4	2	0.515	0.500	0.375
4_BLOCK_20565503_20605570	4	2	0.515	0.500	0.375
4_BLOCK_20637941_20822397	4	2	0.515	0.500	0.375
4_BLOCK_20891905_20891925	4	2	0.515	0.500	0.375
4_BLOCK_20910535_20948382	4	3	0.485	0.621	0.546
S4_21089615	4	2	0.576	0.489	0.369
S4_21373827	4	2	0.879	0.213	0.190
4_BLOCK_21404851_21404855	4	2	0.879	0.213	0.190
4_BLOCK_21507256_21640100	4	3	0.515	0.610	0.537
S4_21663324	4	2	0.848	0.257	0.224
4_BLOCK_21880821_21885479	4	2	0.697	0.422	0.333
S4_21913302	4	2	0.636	0.463	0.356
S4_21970429	4	2	0.818	0.298	0.253
S4_22025095	4	2	0.758	0.367	0.300
4_BLOCK_22176511_22233555	4	2	0.545	0.496	0.373
S4_22408653	4	2	0.909	0.165	0.152
S4_22428157	4	2	0.606	0.478	0.363

4_BLOCK_22464187_22641150	4	2	0.606	0.478	0.363
4_BLOCK_23165656_23290067	4	2	0.606	0.478	0.363
S4_23306219	4	2	0.879	0.213	0.190
4_BLOCK_23481366_23481367	4	2	0.788	0.334	0.278
4_BLOCK_23508539_23626665	4	3	0.485	0.621	0.546
4_BLOCK_23856934_24054770	4	2	0.515	0.500	0.375
S4_24054783	4	2	0.879	0.213	0.190
4_BLOCK_24077748_24077754	4	2	0.515	0.500	0.375
4_BLOCK_24113495_24305626	4	2	0.667	0.444	0.346
S4_24348407	4	2	0.636	0.463	0.356
4_BLOCK_24461440_24644645	4	2	0.515	0.500	0.375
4_BLOCK_24656272_24856039	4	2	0.515	0.500	0.375
S4_24944619	4	2	0.515	0.500	0.375
S4_24987487	4	2	0.515	0.500	0.375
S4_25218058	4	2	0.576	0.489	0.369
4_BLOCK_25235709_25422291	4	2	0.515	0.500	0.375
S4_26344809	4	2	0.788	0.334	0.278
S4_26537343	4	2	0.818	0.298	0.253
S4_26559270	4	2	0.545	0.496	0.373
S4_27107735	4	2	0.818	0.298	0.253
4_BLOCK_27246052_27363791	4	2	0.606	0.478	0.363
4_BLOCK_27375131_27568602	4	3	0.576	0.562	0.489
4_BLOCK_27595686_27698917	4	3	0.576	0.571	0.503
4_BLOCK_27732096_27932020	4	3	0.606	0.551	0.488
S4_28013337	4	2	0.818	0.298	0.253
4_BLOCK_28067001_28147319	4	2	0.515	0.500	0.375
4_BLOCK_28189746_28389257	4	2	0.515	0.500	0.375
4_BLOCK_28439803_28440291	4	2	0.515	0.500	0.375
4_BLOCK_28487164_28676834	4	3	0.485	0.628	0.556
4_BLOCK_28679149_28877910	4	2	0.515	0.500	0.375
4_BLOCK_28910566_29088097	4	2	0.515	0.500	0.375
4_BLOCK_29111830_29119507	4	2	0.515	0.500	0.375
4_BLOCK_29142374_29340076	4	3	0.485	0.595	0.511
S4_29355978	4	2	0.879	0.213	0.190
4_BLOCK_29365468_29564075	4	3	0.485	0.595	0.511
4_BLOCK_29632434_29711763	4	2	0.515	0.500	0.375
S4_29715091	4	2	0.515	0.500	0.375
4_BLOCK_29858777_29965891	4	3	0.606	0.555	0.494
4_BLOCK_29968490_30168451	4	3	0.515	0.615	0.545
4_BLOCK_30168591_30169461	4	2	0.606	0.478	0.363

4_BLOCK_30192094_30386272	4	2	0.515	0.500	0.375
S4_30477127	4	2	0.515	0.500	0.375
S4_30483194	4	2	0.879	0.213	0.190
S4_30581991	4	2	0.606	0.478	0.363
S4_30642693	4	2	0.606	0.478	0.363
S4_30687900	4	2	0.515	0.500	0.375
4_BLOCK_30996754_31113356	4	3	0.485	0.595	0.511
S4_31188042	4	2	0.576	0.489	0.369
S4_31261398	4	2	0.788	0.334	0.278
4_BLOCK_31261400_31423655	4	4	0.394	0.718	0.669
S4_31439312	4	2	0.515	0.500	0.375
4_BLOCK_31446553_31446562	4	2	0.788	0.334	0.278
S4_31446579	4	2	0.818	0.298	0.253
S4_31493805	4	2	0.788	0.334	0.278
4_BLOCK_31632733_31812422	4	3	0.515	0.617	0.548
S4_31820600	4	2	0.879	0.213	0.190
4_BLOCK_31842220_32019553	4	4	0.303	0.744	0.696
S4_32049292	4	2	0.788	0.334	0.278
4_BLOCK_32051279_32061201	4	2	0.576	0.489	0.369
4_BLOCK_32061226_32064832	4	2	0.879	0.213	0.190
4_BLOCK_32092536_32092564	4	2	0.818	0.298	0.253
4_BLOCK_32144497_32252127	4	3	0.485	0.595	0.511
4_BLOCK_32347541_32358525	4	2	0.879	0.213	0.190
S4_32391428	4	2	0.697	0.422	0.333
4_BLOCK_32411131_32605147	4	3	0.485	0.628	0.556
4_BLOCK_32624873_32624908	4	2	0.788	0.334	0.278
4_BLOCK_32760098_32948488	4	2	0.515	0.500	0.375
4_BLOCK_33020703_33073447	4	2	0.515	0.500	0.375
4_BLOCK_33128044_33326841	4	2	0.576	0.489	0.369
4_BLOCK_33337373_33448911	4	2	0.515	0.500	0.375
4_BLOCK_33528451_33725105	4	2	0.515	0.500	0.375
4_BLOCK_33728866_33868949	4	3	0.485	0.628	0.556
S4_34038925	4	2	0.515	0.500	0.375
4_BLOCK_34316267_34506731	4	2	0.515	0.500	0.375
4_BLOCK_34587217_34783534	4	3	0.485	0.610	0.531
4_BLOCK_34794886_34872572	4	3	0.485	0.610	0.531
4_BLOCK_34914313_35086036	4	3	0.485	0.628	0.556
4_BLOCK_35116044_35245834	4	2	0.515	0.500	0.375
4_BLOCK_35271856_35469308	4	2	0.515	0.500	0.375
5_BLOCK_103237_278074	5	2	0.515	0.500	0.375

5_BLOCK_433016_548611	5	3	0.485	0.595	0.511
5_BLOCK_578975_777432	5	3	0.515	0.601	0.524
5_BLOCK_989770_1180798	5	2	0.515	0.500	0.375
5_BLOCK_1190162_1195858	5	2	0.515	0.500	0.375
5_BLOCK_1228866_1419529	5	2	0.515	0.500	0.375
5_BLOCK_1494420_1688535	5	2	0.515	0.500	0.375
5_BLOCK_1757663_1848337	5	2	0.515	0.500	0.375
5_BLOCK_1899095_2050660	5	2	0.515	0.500	0.375
5_BLOCK_2061347_2251987	5	2	0.515	0.500	0.375
5_BLOCK_2494602_2598888	5	2	0.515	0.500	0.375
5_BLOCK_2657581_2840215	5	3	0.485	0.595	0.511
5_BLOCK_3057426_3066279	5	2	0.606	0.478	0.363
S5_3207628	5	2	0.515	0.500	0.375
S5_3294263	5	2	0.636	0.463	0.356
S5_3307402	5	2	0.848	0.257	0.224
5_BLOCK_3352795_3548824	5	4	0.485	0.669	0.618
5_BLOCK_3565216_3629201	5	2	0.576	0.489	0.369
S5_3640106	5	2	0.909	0.165	0.152
S5_3648173	5	2	0.879	0.213	0.190
5_BLOCK_3673333_3842804	5	2	0.576	0.489	0.369
5_BLOCK_4075995_4092570	5	2	0.515	0.500	0.375
5_BLOCK_4094032_4185151	5	3	0.576	0.579	0.515
S5_4205575	5	2	0.879	0.213	0.190
S5_4266430	5	2	0.515	0.500	0.375
5_BLOCK_4303522_4303527	5	2	0.576	0.489	0.369
S5_4569194	5	2	0.515	0.500	0.375
5_BLOCK_4588815_4782402	5	3	0.485	0.632	0.560
5_BLOCK_4838077_4934882	5	2	0.758	0.367	0.300
5_BLOCK_5394309_5554279	5	2	0.515	0.500	0.375
5_BLOCK_5791024_5821403	5	3	0.424	0.617	0.536
S5_5871048	5	2	0.576	0.489	0.369
S5_5912385	5	2	0.909	0.165	0.152
5_BLOCK_5995844_6081211	5	2	0.697	0.422	0.333
S5_6211231	5	2	0.879	0.213	0.190
5_BLOCK_6314318_6398190	5	3	0.515	0.610	0.537
S5_6555463	5	2	0.697	0.422	0.333
S5_6576485	5	2	0.636	0.463	0.356
S5_6591515	5	2	0.818	0.298	0.253
S5_6607309	5	2	0.788	0.334	0.278
S5_6607326	5	2	0.697	0.422	0.333

S5_6660172	5	2	0.697	0.422	0.333
S5_6768706	5	2	0.515	0.500	0.375
5_BLOCK_6791185_6982283	5	3	0.515	0.588	0.506
5_BLOCK_7004230_7004330	5	2	0.515	0.500	0.375
5_BLOCK_7058066_7209324	5	3	0.424	0.617	0.536
5_BLOCK_7214419_7214421	5	2	0.515	0.500	0.375
S5_7250405	5	2	0.879	0.213	0.190
5_BLOCK_7277635_7277671	5	2	0.606	0.478	0.363
S5_7344597	5	2	0.545	0.496	0.373
5_BLOCK_7516118_7626450	5	2	0.515	0.500	0.375
S5_7756614	5	2	0.818	0.298	0.253
S5_7944289	5	2	0.515	0.500	0.375
S5_8005657	5	2	0.758	0.367	0.300
5_BLOCK_8039100_8159644	5	2	0.636	0.463	0.356
5_BLOCK_8447733_8451112	5	2	0.697	0.422	0.333
5_BLOCK_8492836_8691122	5	3	0.576	0.562	0.489
5_BLOCK_8693934_8693971	5	2	0.576	0.489	0.369
5_BLOCK_8773949_8957956	5	2	0.576	0.489	0.369
S5_9104709	5	2	0.848	0.257	0.224
S5_9151692	5	2	0.848	0.257	0.224
5_BLOCK_9184027_9354784	5	2	0.879	0.213	0.190
S5_9354799	5	2	0.848	0.257	0.224
5_BLOCK_9455785_9649642	5	2	0.879	0.213	0.190
S5_10269325	5	2	0.909	0.165	0.152
S5_10429784	5	2	0.545	0.496	0.373
S5_10914207	5	2	0.909	0.165	0.152
S5_12605244	5	2	0.909	0.165	0.152
S5_13259819	5	2	0.909	0.165	0.152
S5_13458628	5	2	0.909	0.165	0.152
S5_13944290	5	2	0.515	0.500	0.375
5_BLOCK_14307666_14470301	5	2	0.545	0.496	0.373
5_BLOCK_14593557_14785281	5	2	0.545	0.496	0.373
S5_14850103	5	2	0.576	0.489	0.369
S5_14948395	5	2	0.545	0.496	0.373
5_BLOCK_14993143_15166382	5	2	0.515	0.500	0.375
S5_15468517	5	2	0.576	0.489	0.369
S5_15686331	5	2	0.576	0.489	0.369
S5_15787927	5	2	0.576	0.489	0.369
5_BLOCK_15868742_16046528	5	2	0.606	0.478	0.363
S5_16181739	5	2	0.576	0.489	0.369

5_BLOCK_16327874_16474415	5	3	0.424	0.654	0.580
S5_16517568	5	2	0.515	0.500	0.375
S5_16671007	5	2	0.667	0.444	0.346
S5_16691086	5	2	0.515	0.500	0.375
S5_16794517	5	2	0.758	0.367	0.300
S5_16794530	5	2	0.667	0.444	0.346
S5_16916177	5	2	0.848	0.257	0.224
5_BLOCK_17053794_17161790	5	2	0.515	0.500	0.375
5_BLOCK_17195159_17362058	5	4	0.394	0.714	0.664
5_BLOCK_17453113_17649763	5	2	0.545	0.496	0.373
5_BLOCK_17684537_17803449	5	2	0.606	0.478	0.363
5_BLOCK_17852949_18047526	5	2	0.606	0.478	0.363
S5_18087808	5	2	0.606	0.478	0.363
5_BLOCK_18181480_18182265	5	2	0.788	0.334	0.278
5_BLOCK_18336720_18399139	5	3	0.485	0.628	0.556
S5_18444811	5	2	0.848	0.257	0.224
5_BLOCK_18444813_18628562	5	3	0.455	0.615	0.535
5_BLOCK_18754355_18766782	5	3	0.455	0.628	0.551
5_BLOCK_18807571_18997029	5	3	0.455	0.637	0.563
5_BLOCK_19041892_19240325	5	3	0.455	0.628	0.551
5_BLOCK_19259974_19426650	5	2	0.545	0.496	0.373
5_BLOCK_19450074_19646259	5	3	0.424	0.643	0.567
5_BLOCK_19678608_19744786	5	3	0.424	0.643	0.567
5_BLOCK_19794240_19961728	5	3	0.455	0.599	0.513
5_BLOCK_19963140_20162768	5	2	0.576	0.489	0.369
S5_20169127	5	2	0.576	0.489	0.369
5_BLOCK_20192094_20377233	5	2	0.576	0.489	0.369
S5_20407158	5	2	0.576	0.489	0.369
5_BLOCK_20440406_20639347	5	3	0.424	0.654	0.580
S5_20659055	5	2	0.576	0.489	0.369
5_BLOCK_20782896_20929538	5	2	0.576	0.489	0.369
5_BLOCK_20940853_21140333	5	2	0.576	0.489	0.369
S5_21145083	5	2	0.515	0.500	0.375
5_BLOCK_21162683_21345487	5	3	0.485	0.610	0.531
5_BLOCK_21472826_21668548	5	3	0.485	0.610	0.531
S5_21798367	5	2	0.515	0.500	0.375
S5_21882302	5	2	0.879	0.213	0.190
S5_21896115	5	2	0.606	0.478	0.363
5_BLOCK_21930884_22126790	5	3	0.485	0.595	0.511
S5_22142330	5	2	0.515	0.500	0.375

5_BLOCK_22142373_22294580	5	3	0.485	0.628	0.556
5_BLOCK_22330204_22529103	5	3	0.485	0.632	0.560
5_BLOCK_22574827_22584842	5	3	0.485	0.595	0.511
5_BLOCK_22662724_22838011	5	3	0.485	0.595	0.511
S5_22873637	5	2	0.515	0.500	0.375
S5_22933650	5	2	0.515	0.500	0.375
5_BLOCK_22975405_23162761	5	2	0.515	0.500	0.375
5_BLOCK_23179788_23218617	5	2	0.576	0.489	0.369
S5_23293656	5	2	0.636	0.463	0.356
5_BLOCK_23311211_23496438	5	3	0.515	0.588	0.506
5_BLOCK_23582685_23582702	5	2	0.545	0.496	0.373
5_BLOCK_23643715_23657422	5	2	0.879	0.213	0.190
S5_23781830	5	2	0.515	0.500	0.375
S5_23927823	5	2	0.515	0.500	0.375
5_BLOCK_23958959_24157035	5	3	0.485	0.632	0.560
5_BLOCK_24197318_24288587	5	2	0.576	0.489	0.369
5_BLOCK_24371022_24566769	5	2	0.515	0.500	0.375
S5_24627396	5	2	0.515	0.500	0.375
5_BLOCK_24637231_24835452	5	2	0.515	0.500	0.375
S5_24853754	5	2	0.515	0.500	0.375
S5_24908936	5	2	0.515	0.500	0.375
5_BLOCK_24991225_25189984	5	2	0.515	0.500	0.375
5_BLOCK_25194958_25282570	5	2	0.515	0.500	0.375
5_BLOCK_25284057_25484000	5	2	0.515	0.500	0.375
5_BLOCK_25552006_25686498	5	2	0.515	0.500	0.375
5_BLOCK_25842796_26029716	5	2	0.515	0.500	0.375
S5_26093864	5	2	0.515	0.500	0.375
S5_26116353	5	2	0.515	0.500	0.375
S5_26193353	5	2	0.515	0.500	0.375
5_BLOCK_26199389_26397257	5	2	0.545	0.496	0.373
5_BLOCK_26415155_26614363	5	2	0.545	0.496	0.373
S5_26624719	5	2	0.545	0.496	0.373
S5_26716093	5	2	0.515	0.500	0.375
5_BLOCK_26776676_26974078	5	3	0.545	0.577	0.499
S5_27063508	5	2	0.697	0.422	0.333
S5_27063529	5	2	0.848	0.257	0.224
S5_27220339	5	2	0.697	0.422	0.333
5_BLOCK_27321815_27521160	5	2	0.515	0.500	0.375
S5_27539634	5	2	0.545	0.496	0.373
5_BLOCK_27540075_27617072	5	2	0.515	0.500	0.375

5_BLOCK_27661282_27861129	5	3	0.455	0.599	0.513
S5_27877004	5	2	0.667	0.444	0.346
S5_27886749	5	2	0.515	0.500	0.375
5_BLOCK_27886769_28020620	5	3	0.485	0.621	0.546
5_BLOCK_28073161_28273117	5	3	0.455	0.637	0.563
5_BLOCK_28273176_28324130	5	2	0.545	0.496	0.373
5_BLOCK_28346250_28346268	5	2	0.848	0.257	0.224
5_BLOCK_28389026_28455342	5	2	0.545	0.496	0.373
5_BLOCK_28472424_28670241	5	3	0.455	0.628	0.551
5_BLOCK_28683037_28831954	5	2	0.545	0.496	0.373
5_BLOCK_28914195_28931704	5	2	0.515	0.500	0.375
5_BLOCK_29004623_29203515	5	4	0.364	0.727	0.678
S5_29209871	5	2	0.515	0.500	0.375
S5_29241279	5	2	0.818	0.298	0.253
S5_29311170	5	2	0.515	0.500	0.375
S5_29364774	5	2	0.515	0.500	0.375
S5_29364793	5	2	0.515	0.500	0.375
S5_29382884	5	2	0.667	0.444	0.346
5_BLOCK_29383560_29579134	5	2	0.545	0.496	0.373
S5_29597255	5	2	0.848	0.257	0.224
S5_29808911	5	2	0.515	0.500	0.375
S6_170185	6	2	0.515	0.500	0.375
S6_291405	6	2	0.515	0.500	0.375
S6_294858	6	2	0.667	0.444	0.346
6_BLOCK_411985_585557	6	3	0.515	0.615	0.545
S6_698086	6	2	0.606	0.478	0.363
6_BLOCK_738449_922752	6	2	0.606	0.478	0.363
6_BLOCK_970592_1159740	6	2	0.697	0.422	0.333
6_BLOCK_1208254_1361837	6	2	0.606	0.478	0.363
6_BLOCK_1374317_1573444	6	2	0.606	0.478	0.363
6_BLOCK_1585783_1681086	6	2	0.606	0.478	0.363
6_BLOCK_1760469_1959576	6	2	0.606	0.478	0.363
6_BLOCK_1968874_2117604	6	2	0.606	0.478	0.363
S6_2123411	6	2	0.758	0.367	0.300
6_BLOCK_2191272_2377585	6	2	0.545	0.496	0.373
6_BLOCK_2395083_2594113	6	2	0.545	0.496	0.373
6_BLOCK_2655251_2849385	6	2	0.545	0.496	0.373
S6_2889739	6	2	0.636	0.463	0.356
6_BLOCK_2940130_2946783	6	2	0.636	0.463	0.356
6_BLOCK_3057773_3243035	6	2	0.545	0.496	0.373

6_BLOCK_3314007_3338706	6	2	0.576	0.489	0.369
6_BLOCK_3339544_3537854	6	3	0.576	0.571	0.503
6_BLOCK_3542566_3583430	6	3	0.576	0.571	0.503
6_BLOCK_4202769_4402154	6	3	0.455	0.599	0.513
6_BLOCK_4487539_4655511	6	2	0.545	0.496	0.373
6_BLOCK_4704941_4720879	6	2	0.515	0.500	0.375
S6_4802988	6	2	0.636	0.463	0.356
S6_4813022	6	2	0.758	0.367	0.300
S6_4854279	6	2	0.758	0.367	0.300
S6_4888111	6	2	0.606	0.478	0.363
S6_4890290	6	2	0.788	0.334	0.278
S6_4991701	6	2	0.788	0.334	0.278
6_BLOCK_4998720_5198308	6	2	0.788	0.334	0.278
6_BLOCK_5260332_5348684	6	2	0.788	0.334	0.278
6_BLOCK_5660320_5732592	6	2	0.576	0.489	0.369
6_BLOCK_6091334_6253559	6	2	0.515	0.500	0.375
6_BLOCK_6292721_6294204	6	2	0.576	0.489	0.369
6_BLOCK_6352098_6551261	6	3	0.485	0.621	0.546
S6_6638922	6	2	0.576	0.489	0.369
S6_6688064	6	2	0.515	0.500	0.375
6_BLOCK_6737291_6924265	6	3	0.515	0.615	0.545
S6_6988053	6	2	0.515	0.500	0.375
S6_7045507	6	2	0.848	0.257	0.224
S6_7088738	6	2	0.515	0.500	0.375
S6_7113667	6	2	0.848	0.257	0.224
6_BLOCK_7132754_7319696	6	3	0.515	0.588	0.506
6_BLOCK_7498571_7537460	6	2	0.515	0.500	0.375
S6_7730845	6	2	0.636	0.463	0.356
S6_7843151	6	2	0.818	0.298	0.253
6_BLOCK_7843157_8034318	6	3	0.455	0.599	0.513
6_BLOCK_8043560_8162879	6	4	0.333	0.722	0.671
S6_8332175	6	2	0.515	0.500	0.375
S6_8332180	6	2	0.667	0.444	0.346
6_BLOCK_8332214_8342969	6	2	0.818	0.298	0.253
S6_8382386	6	2	0.667	0.444	0.346
6_BLOCK_8409631_8599271	6	3	0.485	0.621	0.546
6_BLOCK_8622843_8646263	6	4	0.364	0.709	0.655
6_BLOCK_8728735_8914671	6	3	0.485	0.621	0.546
S6_8948220	6	2	0.788	0.334	0.278
S6_9205228	6	2	0.515	0.500	0.375

6_BLOCK_9317529_9506708	6	2	0.515	0.500	0.375
6_BLOCK_9827682_10016501	6	4	0.394	0.705	0.652
6_BLOCK_10028703_10147242	6	3	0.394	0.661	0.587
S6_10203325	6	2	0.848	0.257	0.224
S6_10474039	6	2	0.758	0.367	0.300
6_BLOCK_10487577_10648832	6	2	0.606	0.478	0.363
6_BLOCK_10740899_10874676	6	3	0.485	0.595	0.511
S6_10906893	6	2	0.879	0.213	0.190
S6_10960287	6	2	0.788	0.334	0.278
S6_10974808	6	2	0.576	0.489	0.369
S6_11017200	6	2	0.848	0.257	0.224
6_BLOCK_11609001_11698312	6	2	0.576	0.489	0.369
6_BLOCK_11842577_12038704	6	2	0.515	0.500	0.375
S6_12062226	6	2	0.515	0.500	0.375
S6_12086486	6	2	0.576	0.489	0.369
6_BLOCK_12187330_12323681	6	2	0.606	0.478	0.363
6_BLOCK_12646228_12755088	6	2	0.515	0.500	0.375
S6_12825720	6	2	0.909	0.165	0.152
S6_12838548	6	2	0.909	0.165	0.152
S6_12860755	6	2	0.515	0.500	0.375
6_BLOCK_13055212_13055235	6	2	0.515	0.500	0.375
6_BLOCK_13205579_13375845	6	2	0.515	0.500	0.375
6_BLOCK_13412761_13412765	6	2	0.515	0.500	0.375
6_BLOCK_13500246_13698579	6	2	0.515	0.500	0.375
6_BLOCK_13835075_13989607	6	2	0.515	0.500	0.375
6_BLOCK_13996048_14195288	6	2	0.515	0.500	0.375
S6_14195335	6	2	0.879	0.213	0.190
S6_14195353	6	2	0.879	0.213	0.190
6_BLOCK_14332734_14385969	6	3	0.485	0.628	0.556
6_BLOCK_14640948_14640965	6	3	0.485	0.621	0.546
S6_15181849	6	2	0.515	0.500	0.375
S6_15181865	6	2	0.818	0.298	0.253
6_BLOCK_15733820_15921301	6	3	0.485	0.621	0.546
S6_15970091	6	2	0.848	0.257	0.224
6_BLOCK_16025528_16211742	6	2	0.515	0.500	0.375
6_BLOCK_16250416_16250430	6	2	0.576	0.489	0.369
S6_16441433	6	2	0.576	0.489	0.369
6_BLOCK_16472294_16657203	6	2	0.576	0.489	0.369
6_BLOCK_16804332_16881361	6	2	0.515	0.500	0.375
6_BLOCK_16980842_17173070	6	3	0.485	0.595	0.511

6_BLOCK_17194341_17257500	6	2	0.515	0.500	0.375
6_BLOCK_17500839_17698517	6	2	0.515	0.500	0.375
6_BLOCK_17732039_17910074	6	2	0.515	0.500	0.375
S6_17934214	6	2	0.515	0.500	0.375
6_BLOCK_17947054_18143876	6	2	0.515	0.500	0.375
S6_18451913	6	2	0.515	0.500	0.375
6_BLOCK_18902619_19023634	6	2	0.515	0.500	0.375
6_BLOCK_19323415_19492195	6	2	0.545	0.496	0.373
6_BLOCK_19740264_19868165	6	3	0.485	0.628	0.556
6_BLOCK_19951253_20137932	6	2	0.515	0.500	0.375
S6_20159515	6	2	0.515	0.500	0.375
6_BLOCK_20454921_20501183	6	3	0.485	0.595	0.511
S6_20707820	6	2	0.515	0.500	0.375
S6_20737593	6	2	0.515	0.500	0.375
6_BLOCK_20833426_21031879	6	2	0.515	0.500	0.375
6_BLOCK_21061104_21079858	6	2	0.515	0.500	0.375
S6_21082446	6	2	0.848	0.257	0.224
6_BLOCK_21191599_21370594	6	3	0.515	0.601	0.524
6_BLOCK_21539402_21539419	6	2	0.848	0.257	0.224
S6_21552893	6	2	0.848	0.257	0.224
S6_21703315	6	2	0.727	0.397	0.318
S6_21963718	6	2	0.697	0.422	0.333
S6_22279338	6	2	0.606	0.478	0.363
S6_22315623	6	2	0.879	0.213	0.190
S6_22363525	6	2	0.545	0.496	0.373
S6_22491379	6	2	0.545	0.496	0.373
6_BLOCK_22656024_22657411	6	3	0.394	0.645	0.569
6_BLOCK_22813935_22813976	6	3	0.485	0.628	0.556
6_BLOCK_22826624_23001271	6	2	0.576	0.489	0.369
6_BLOCK_23039886_23237994	6	4	0.333	0.722	0.671
6_BLOCK_23243204_23365001	6	4	0.333	0.722	0.671
S6_23442318	6	2	0.515	0.500	0.375
6_BLOCK_23451739_23451826	6	2	0.788	0.334	0.278
6_BLOCK_23500117_23519664	6	3	0.485	0.621	0.546
S6_23707453	6	2	0.576	0.489	0.369
6_BLOCK_23780104_23780127	6	2	0.545	0.496	0.373
S6_23780178	6	2	0.606	0.478	0.363
S6_23873865	6	2	0.515	0.500	0.375
S6_23913208	6	2	0.515	0.500	0.375
6_BLOCK_24005516_24187203	6	2	0.515	0.500	0.375

S6_24211955	6	2	0.515	0.500	0.375
S6_24403444	6	2	0.515	0.500	0.375
6_BLOCK_24517511_24716050	6	2	0.576	0.489	0.369
6_BLOCK_24762716_24907850	6	3	0.455	0.615	0.535
6_BLOCK_24949417_25096402	6	3	0.455	0.615	0.535
6_BLOCK_25170875_25177407	6	3	0.455	0.599	0.513
6_BLOCK_25210249_25389950	6	2	0.667	0.444	0.346
6_BLOCK_25737829_25790397	6	2	0.515	0.500	0.375
6_BLOCK_25905803_26098064	6	2	0.515	0.500	0.375
S6_26106383	6	2	0.515	0.500	0.375
6_BLOCK_26286901_26460009	6	2	0.515	0.500	0.375
6_BLOCK_26508305_26708220	6	3	0.515	0.588	0.506
S6_26778074	6	2	0.515	0.500	0.375
S6_26819217	6	2	0.515	0.500	0.375
6_BLOCK_27037751_27053504	6	2	0.515	0.500	0.375
6_BLOCK_27075368_27255126	6	2	0.515	0.500	0.375
6_BLOCK_27305456_27498819	6	2	0.515	0.500	0.375
S6_27530949	6	2	0.515	0.500	0.375
S6_27632618	6	2	0.818	0.298	0.253
6_BLOCK_27972059_28017283	6	2	0.788	0.334	0.278
6_BLOCK_28079760_28144543	6	3	0.545	0.577	0.499
6_BLOCK_28172005_28371201	6	2	0.545	0.496	0.373
6_BLOCK_28377309_28380658	6	2	0.515	0.500	0.375
6_BLOCK_28498289_28498297	6	2	0.545	0.496	0.373
6_BLOCK_28503669_28700905	6	3	0.455	0.599	0.513
S6_28703732	6	2	0.545	0.496	0.373
6_BLOCK_28723236_28919533	6	2	0.545	0.496	0.373
6_BLOCK_28952172_29132956	6	3	0.455	0.599	0.513
6_BLOCK_29163287_29358809	6	3	0.455	0.599	0.513
S6_29366320	6	2	0.636	0.463	0.356
S6_29423874	6	2	0.545	0.496	0.373
S6_29473620	6	2	0.606	0.478	0.363
6_BLOCK_29480354_29678694	6	3	0.455	0.637	0.563
6_BLOCK_29693560_29738557	6	2	0.576	0.489	0.369
6_BLOCK_29755504_29951383	6	2	0.576	0.489	0.369
6_BLOCK_29985628_30184940	6	2	0.576	0.489	0.369
S6_30196627	6	2	0.576	0.489	0.369
6_BLOCK_30336155_30512019	6	3	0.394	0.645	0.569
S6_30762430	6	2	0.576	0.489	0.369
6_BLOCK_30782394_30975096	6	3	0.424	0.643	0.567

6_BLOCK_31000883_31197011	6	2	0.576	0.489	0.369
S7_67847	7	2	0.545	0.496	0.373
7_BLOCK_114773_305846	7	5	0.242	0.795	0.762
S7_334301	7	2	0.848	0.257	0.224
S7_353159	7	2	0.788	0.334	0.278
S7_364849	7	2	0.848	0.257	0.224
7_BLOCK_367318_510646	7	3	0.394	0.654	0.579
S7_630899	7	2	0.636	0.463	0.356
7_BLOCK_631017_824464	7	2	0.636	0.463	0.356
S7_824478	7	2	0.879	0.213	0.190
7_BLOCK_930068_1027624	7	2	0.545	0.496	0.373
7_BLOCK_1048877_1248306	7	2	0.545	0.496	0.373
7_BLOCK_1500602_1696894	7	3	0.424	0.632	0.554
7_BLOCK_1701760_1717770	7	3	0.424	0.643	0.567
S7_1717803	7	2	0.606	0.478	0.363
S7_1717822	7	2	0.788	0.334	0.278
7_BLOCK_1926189_1991541	7	2	0.636	0.463	0.356
7_BLOCK_2034837_2216212	7	2	0.606	0.478	0.363
S7_2258819	7	2	0.606	0.478	0.363
7_BLOCK_2339243_2524412	7	2	0.515	0.500	0.375
7_BLOCK_2565701_2691107	7	2	0.515	0.500	0.375
7_BLOCK_3180647_3180736	7	2	0.788	0.334	0.278
7_BLOCK_3298460_3343497	7	2	0.515	0.500	0.375
S7_3397808	7	2	0.879	0.213	0.190
S7_3433329	7	2	0.879	0.213	0.190
S7_3447454	7	2	0.879	0.213	0.190
S7_3834570	7	2	0.545	0.496	0.373
S7_3988698	7	2	0.545	0.496	0.373
7_BLOCK_4272700_4425469	7	2	0.545	0.496	0.373
7_BLOCK_4499836_4687038	7	2	0.576	0.489	0.369
7_BLOCK_4704118_4776414	7	3	0.455	0.599	0.513
7_BLOCK_5179524_5348724	7	2	0.576	0.489	0.369
S7_5419423	7	2	0.576	0.489	0.369
S7_5559218	7	2	0.576	0.489	0.369
7_BLOCK_5777342_5956815	7	2	0.545	0.496	0.373
S7_6108654	7	2	0.879	0.213	0.190
S7_6232714	7	2	0.879	0.213	0.190
S7_6298841	7	2	0.879	0.213	0.190
S7_6301473	7	2	0.909	0.165	0.152
S7_6301487	7	2	0.909	0.165	0.152

7_BLOCK_6748850_6753276	7	3	0.485	0.632	0.560
7_BLOCK_6779615_6958829	7	3	0.455	0.599	0.513
7_BLOCK_6963821_6967818	7	2	0.636	0.463	0.356
S7_6967860	7	2	0.818	0.298	0.253
S7_6968832	7	2	0.636	0.463	0.356
S7_6993264	7	2	0.606	0.478	0.363
7_BLOCK_7003921_7186204	7	2	0.545	0.496	0.373
7_BLOCK_7366670_7474224	7	2	0.515	0.500	0.375
7_BLOCK_7549366_7739987	7	3	0.515	0.588	0.506
S7_7794071	7	2	0.879	0.213	0.190
7_BLOCK_7815819_7832772	7	3	0.515	0.588	0.506
7_BLOCK_7923605_8079406	7	2	0.606	0.478	0.363
S7_8320872	7	2	0.879	0.213	0.190
S7_8322755	7	2	0.515	0.500	0.375
S7_8360549	7	2	0.667	0.444	0.346
S7_8471410	7	2	0.818	0.298	0.253
7_BLOCK_8524014_8546054	7	2	0.545	0.496	0.373
S7_8815355	7	2	0.758	0.367	0.300
S7_8857486	7	2	0.697	0.422	0.333
7_BLOCK_9004296_9198157	7	3	0.485	0.628	0.556
S7_9198205	7	2	0.788	0.334	0.278
S7_9357411	7	2	0.545	0.496	0.373
7_BLOCK_9972678_9972706	7	3	0.485	0.621	0.546
7_BLOCK_9973262_10170349	7	3	0.455	0.637	0.563
S7_10299244	7	2	0.545	0.496	0.373
S7_10311727	7	2	0.667	0.444	0.346
S7_10637214	7	2	0.515	0.500	0.375
7_BLOCK_10955145_10955150	7	2	0.848	0.257	0.224
S7_10955266	7	2	0.515	0.500	0.375
S7_10979899	7	2	0.879	0.213	0.190
7_BLOCK_10991803_11175112	7	3	0.515	0.588	0.506
7_BLOCK_11255207_11255252	7	2	0.636	0.463	0.356
7_BLOCK_11385047_11561321	7	2	0.545	0.496	0.373
S7_11627960	7	2	0.515	0.500	0.375
S7_11650258	7	2	0.636	0.463	0.356
7_BLOCK_12684285_12773324	7	3	0.515	0.588	0.506
S7_13320608	7	2	0.545	0.496	0.373
S7_13391875	7	2	0.818	0.298	0.253
S7_13443683	7	2	0.636	0.463	0.356
7_BLOCK_13602658_13765974	7	3	0.515	0.588	0.506

7_BLOCK_13772943_13842947	7	2	0.818	0.298	0.253
S7_14502565	7	2	0.848	0.257	0.224
S7_14533308	7	2	0.636	0.463	0.356
S7_14541483	7	2	0.515	0.500	0.375
S7_14634551	7	2	0.545	0.496	0.373
S7_14814925	7	2	0.576	0.489	0.369
7_BLOCK_14973557_15075474	7	2	0.515	0.500	0.375
S7_15296596	7	2	0.848	0.257	0.224
7_BLOCK_15359641_15511213	7	3	0.455	0.599	0.513
S7_15584083	7	2	0.606	0.478	0.363
7_BLOCK_15785592_15887798	7	3	0.394	0.645	0.569
S7_15931676	7	2	0.727	0.397	0.318
S7_16071250	7	2	0.606	0.478	0.363
S7_16109913	7	2	0.545	0.496	0.373
S7_16109928	7	2	0.848	0.257	0.224
7_BLOCK_16173112_16365344	7	4	0.455	0.681	0.629
S7_16894880	7	2	0.576	0.489	0.369
S7_16924008	7	2	0.848	0.257	0.224
7_BLOCK_17131083_17311737	7	2	0.576	0.489	0.369
7_BLOCK_17439816_17455113	7	2	0.576	0.489	0.369
7_BLOCK_17526780_17684946	7	2	0.576	0.489	0.369
S7_17690335	7	2	0.848	0.257	0.224
S7_17690362	7	2	0.788	0.334	0.278
S7_17811135	7	2	0.818	0.298	0.253
S7_18113774	7	2	0.636	0.463	0.356
7_BLOCK_18114148_18115222	7	3	0.455	0.615	0.535
7_BLOCK_18121672_18240947	7	3	0.364	0.661	0.587
S7_18362083	7	2	0.515	0.500	0.375
S7_18412863	7	2	0.576	0.489	0.369
7_BLOCK_18456209_18512310	7	3	0.545	0.577	0.499
S7_18631191	7	2	0.545	0.496	0.373
7_BLOCK_18728431_18923889	7	3	0.424	0.617	0.536
7_BLOCK_19009198_19091425	7	2	0.576	0.489	0.369
7_BLOCK_19160923_19354599	7	3	0.424	0.632	0.554
7_BLOCK_19412558_19432234	7	2	0.636	0.463	0.356
7_BLOCK_19592108_19762897	7	3	0.636	0.527	0.470
S7_19772377	7	2	0.758	0.367	0.300
7_BLOCK_19888341_20087352	7	3	0.576	0.579	0.515
S7_20207992	7	2	0.758	0.367	0.300
7_BLOCK_20207998_20326900	7	3	0.424	0.650	0.576

S7_20650160	7	2	0.667	0.444	0.346
7_BLOCK_20650181_20826476	7	4	0.485	0.669	0.618
7_BLOCK_20826478_20826500	7	2	0.697	0.422	0.333
7_BLOCK_20959093_20969819	7	3	0.515	0.617	0.548
7_BLOCK_21044860_21238771	7	3	0.515	0.615	0.545
7_BLOCK_21246840_21439926	7	3	0.485	0.628	0.556
7_BLOCK_21472140_21655871	7	3	0.545	0.595	0.526
7_BLOCK_21708542_21708629	7	2	0.515	0.500	0.375
7_BLOCK_21713323_21911418	7	2	0.515	0.500	0.375
7_BLOCK_21923311_21923329	7	2	0.515	0.500	0.375
7_BLOCK_21970253_22170134	7	2	0.515	0.500	0.375
7_BLOCK_22178914_22370841	7	2	0.576	0.489	0.369
7_BLOCK_22505229_22690093	7	2	0.515	0.500	0.375
7_BLOCK_22774278_22774359	7	2	0.576	0.489	0.369
7_BLOCK_22858259_23046439	7	3	0.576	0.577	0.512
S7_23113876	7	2	0.636	0.463	0.356
S7_23146518	7	2	0.636	0.463	0.356
S7_23156737	7	2	0.879	0.213	0.190
S7_23160686	7	2	0.636	0.463	0.356
7_BLOCK_23213774_23402143	7	2	0.515	0.500	0.375
7_BLOCK_23491605_23502788	7	2	0.515	0.500	0.375
7_BLOCK_23858704_23859829	7	2	0.576	0.489	0.369
7_BLOCK_23904447_24104116	7	2	0.515	0.500	0.375
7_BLOCK_24126217_24263730	7	2	0.545	0.496	0.373
S7_24350575	7	2	0.758	0.367	0.300
7_BLOCK_24385360_24576268	7	2	0.606	0.478	0.363
7_BLOCK_24627656_24806954	7	2	0.545	0.496	0.373
7_BLOCK_24938715_25045013	7	2	0.515	0.500	0.375
7_BLOCK_25322927_25509425	7	3	0.485	0.595	0.511
7_BLOCK_25580725_25775018	7	2	0.515	0.500	0.375
7_BLOCK_25871455_26026391	7	3	0.515	0.601	0.524
7_BLOCK_26062155_26258860	7	3	0.515	0.601	0.524
7_BLOCK_26394557_26514034	7	3	0.485	0.595	0.511
7_BLOCK_26549570_26746263	7	3	0.485	0.595	0.511
7_BLOCK_26756643_26819778	7	3	0.485	0.610	0.531
S7_26929982	7	2	0.909	0.165	0.152
7_BLOCK_26963849_27155460	7	4	0.424	0.691	0.636
S7_27330666	7	2	0.848	0.257	0.224
7_BLOCK_27330677_27330694	7	3	0.545	0.588	0.515
S7_27629562	7	2	0.727	0.397	0.318

S7_27629575	7	2	0.606	0.478	0.363
7_BLOCK_27728152_27917280	7	4	0.333	0.736	0.688
7_BLOCK_27929531_28040452	7	2	0.818	0.298	0.253
7_BLOCK_28410254_28551250	7	3	0.515	0.615	0.545
7_BLOCK_28658525_28854695	7	3	0.515	0.601	0.524
7_BLOCK_28885823_29063624	7	3	0.515	0.601	0.524
7_BLOCK_29110550_29309603	7	2	0.515	0.500	0.375
7_BLOCK_29313707_29313721	7	2	0.515	0.500	0.375
7_BLOCK_29341180_29521314	7	2	0.515	0.500	0.375
7_BLOCK_29560837_29593249	7	2	0.515	0.500	0.375
8_BLOCK_20527_167971	8	3	0.455	0.628	0.551
8_BLOCK_185172_376154	8	3	0.455	0.628	0.551
S8_390576	8	2	0.788	0.334	0.278
S8_392281	8	2	0.818	0.298	0.253
S8_422807	8	2	0.667	0.444	0.346
S8_437373	8	2	0.545	0.496	0.373
S8_518501	8	2	0.879	0.213	0.190
S8_648281	8	2	0.879	0.213	0.190
S8_892300	8	2	0.879	0.213	0.190
S8_892330	8	2	0.879	0.213	0.190
S8_943798	8	2	0.848	0.257	0.224
8_BLOCK_1119267_1149412	8	3	0.455	0.599	0.513
S8_1149415	8	2	0.515	0.500	0.375
8_BLOCK_1179769_1293896	8	4	0.455	0.689	0.639
8_BLOCK_1509371_1705646	8	2	0.576	0.489	0.369
8_BLOCK_1748375_1753671	8	2	0.606	0.478	0.363
8_BLOCK_2012570_2033915	8	2	0.545	0.496	0.373
8_BLOCK_2057660_2257334	8	2	0.545	0.496	0.373
8_BLOCK_2270248_2365655	8	2	0.545	0.496	0.373
S8_2785303	8	2	0.727	0.397	0.318
S8_2786777	8	2	0.758	0.367	0.300
S8_2786791	8	2	0.758	0.367	0.300
8_BLOCK_2813743_2813748	8	2	0.727	0.397	0.318
8_BLOCK_2813764_3012135	8	3	0.485	0.632	0.560
S8_3131089	8	2	0.636	0.463	0.356
S8_3131124	8	2	0.758	0.367	0.300
S8_3213140	8	2	0.697	0.422	0.333
S8_3227293	8	2	0.879	0.213	0.190
8_BLOCK_3377540_3539439	8	2	0.667	0.444	0.346
S8_3545625	8	2	0.636	0.463	0.356

S8_3569420	8	2	0.545	0.496	0.373
8_BLOCK_3749605_3788532	8	2	0.545	0.496	0.373
8_BLOCK_3822446_4011456	8	3	0.515	0.601	0.524
8_BLOCK_4062696_4062709	8	2	0.788	0.334	0.278
8_BLOCK_4078946_4278372	8	4	0.455	0.692	0.644
8_BLOCK_4303396_4430945	8	2	0.545	0.496	0.373
8_BLOCK_4571294_4647712	8	2	0.515	0.500	0.375
S8_4709790	8	2	0.879	0.213	0.190
S8_4767926	8	2	0.879	0.213	0.190
8_BLOCK_4849012_4849029	8	3	0.455	0.599	0.513
8_BLOCK_4948258_5147772	8	2	0.515	0.500	0.375
8_BLOCK_5217229_5368173	8	4	0.424	0.702	0.651
S8_5368181	8	2	0.758	0.367	0.300
8_BLOCK_5383861_5572424	8	3	0.485	0.595	0.511
S8_5785963	8	2	0.545	0.496	0.373
8_BLOCK_5980996_5999826	8	3	0.485	0.628	0.556
8_BLOCK_6089564_6242609	8	3	0.455	0.615	0.535
8_BLOCK_6251405_6277158	8	2	0.848	0.257	0.224
S8_6728517	8	2	0.545	0.496	0.373
S8_7160224	8	2	0.545	0.496	0.373
8_BLOCK_7222675_7367795	8	2	0.545	0.496	0.373
8_BLOCK_7660493_7834738	8	4	0.364	0.711	0.658
S8_7834739	8	2	0.636	0.463	0.356
S8_7945987	8	2	0.879	0.213	0.190
S8_8062210	8	2	0.606	0.478	0.363
S8_8085728	8	2	0.636	0.463	0.356
8_BLOCK_8109843_8307744	8	3	0.455	0.599	0.513
8_BLOCK_8320772_8431560	8	2	0.515	0.500	0.375
S8_8435234	8	2	0.879	0.213	0.190
S8_8614900	8	2	0.879	0.213	0.190
S8_8615971	8	2	0.667	0.444	0.346
8_BLOCK_8812758_8951782	8	3	0.424	0.643	0.567
S8_8986534	8	2	0.879	0.213	0.190
S8_8990319	8	2	0.788	0.334	0.278
S8_9040979	8	2	0.545	0.496	0.373
S8_9069532	8	2	0.879	0.213	0.190
8_BLOCK_9100387_9100440	8	2	0.879	0.213	0.190
S8_9123908	8	2	0.515	0.500	0.375
S8_9210125	8	2	0.636	0.463	0.356
S8_9210129	8	2	0.818	0.298	0.253

S8_9226286	8	2	0.636	0.463	0.356
8_BLOCK_9629299_9797228	8	3	0.364	0.661	0.587
8_BLOCK_9797277_9801928	8	2	0.697	0.422	0.333
S8_10026106	8	2	0.848	0.257	0.224
8_BLOCK_10366701_10536394	8	3	0.576	0.571	0.503
S8_10620697	8	2	0.848	0.257	0.224
S8_10620758	8	2	0.576	0.489	0.369
S8_10630465	8	2	0.727	0.397	0.318
8_BLOCK_10685438_10884457	8	4	0.455	0.672	0.616
S8_11031959	8	2	0.545	0.496	0.373
S8_11142119	8	2	0.545	0.496	0.373
S8_11250548	8	2	0.515	0.500	0.375
S8_11564708	8	2	0.545	0.496	0.373
8_BLOCK_11974960_12079951	8	2	0.545	0.496	0.373
S8_13031841	8	2	0.545	0.496	0.373
S8_13120384	8	2	0.515	0.500	0.375
S8_13956746	8	2	0.515	0.500	0.375
S8_14153663	8	2	0.545	0.496	0.373
8_BLOCK_14327460_14327548	8	2	0.515	0.500	0.375
S8_14372764	8	2	0.515	0.500	0.375
8_BLOCK_14403015_14601405	8	3	0.515	0.617	0.548
S8_14703002	8	2	0.515	0.500	0.375
S8_14739841	8	2	0.788	0.334	0.278
8_BLOCK_14909551_15013691	8	3	0.545	0.599	0.532
8_BLOCK_15014559_15176935	8	3	0.545	0.599	0.532
8_BLOCK_15466378_15474378	8	2	0.545	0.496	0.373
8_BLOCK_15666570_15774912	8	2	0.545	0.496	0.373
8_BLOCK_15977128_16073718	8	2	0.515	0.500	0.375
8_BLOCK_16164785_16164792	8	2	0.788	0.334	0.278
8_BLOCK_16164808_16356444	8	3	0.485	0.628	0.556
S8_16401513	8	2	0.848	0.257	0.224
S8_16401555	8	2	0.515	0.500	0.375
S8_16489777	8	2	0.545	0.496	0.373
S8_16523752	8	2	0.879	0.213	0.190
8_BLOCK_16592773_16727754	8	3	0.485	0.621	0.546
8_BLOCK_17074980_17132496	8	3	0.485	0.628	0.556
S8_17136813	8	2	0.667	0.444	0.346
8_BLOCK_17179017_17328188	8	3	0.485	0.628	0.556
8_BLOCK_17511227_17705990	8	3	0.455	0.643	0.570
S8_17732912	8	2	0.515	0.500	0.375

S8_17836123	8	2	0.758	0.367	0.300
8_BLOCK_17852044_18026308	8	3	0.455	0.628	0.551
S8_18161762	8	2	0.545	0.496	0.373
S8_18203437	8	2	0.576	0.489	0.369
S8_18224121	8	2	0.879	0.213	0.190
S8_18243676	8	2	0.545	0.496	0.373
S8_18272781	8	2	0.758	0.367	0.300
8_BLOCK_18404685_18569612	8	2	0.667	0.444	0.346
8_BLOCK_18579377_18777521	8	3	0.606	0.551	0.488
8_BLOCK_18787104_18959270	8	2	0.576	0.489	0.369
8_BLOCK_18993850_18993878	8	2	0.545	0.496	0.373
S8_19080109	8	2	0.545	0.496	0.373
8_BLOCK_19173322_19204611	8	2	0.545	0.496	0.373
8_BLOCK_19220914_19420646	8	2	0.545	0.496	0.373
S8_19435392	8	2	0.879	0.213	0.190
S8_19435475	8	2	0.545	0.496	0.373
8_BLOCK_19556217_19556247	8	2	0.545	0.496	0.373
S8_19573293	8	2	0.576	0.489	0.369
S8_19625403	8	2	0.848	0.257	0.224
8_BLOCK_19719700_19859287	8	3	0.515	0.601	0.524
8_BLOCK_19859289_19859322	8	2	0.515	0.500	0.375
8_BLOCK_19884434_20083234	8	2	0.636	0.463	0.356
S8_20096110	8	2	0.515	0.500	0.375
S8_20150313	8	2	0.909	0.165	0.152
S8_20156246	8	2	0.667	0.444	0.346
S8_20219216	8	2	0.909	0.165	0.152
S8_20385499	8	2	0.788	0.334	0.278
S8_20391635	8	2	0.545	0.496	0.373
8_BLOCK_20459496_20649651	8	3	0.455	0.615	0.535
8_BLOCK_20690040_20853755	8	2	0.515	0.500	0.375
8_BLOCK_20873536_21072281	8	2	0.515	0.500	0.375
8_BLOCK_21108949_21268386	8	2	0.515	0.500	0.375
S8_21414724	8	2	0.515	0.500	0.375
8_BLOCK_21433512_21627617	8	3	0.485	0.621	0.546
S8_21667007	8	2	0.515	0.500	0.375
S8_21851814	8	2	0.545	0.496	0.373
S8_22149823	8	2	0.545	0.496	0.373
8_BLOCK_22365510_22507063	8	2	0.545	0.496	0.373
8_BLOCK_22688814_22887904	8	2	0.545	0.496	0.373
S8_22945050	8	2	0.545	0.496	0.373

8_BLOCK_23013797_23030989	8	2	0.545	0.496	0.373
8_BLOCK_23046436_23241542	8	2	0.545	0.496	0.373
8_BLOCK_23386935_23586361	8	2	0.545	0.496	0.373
S8_23614213	8	2	0.545	0.496	0.373
8_BLOCK_23650185_23656017	8	2	0.545	0.496	0.373
S8_24084321	8	2	0.636	0.463	0.356
S8_24476709	8	2	0.515	0.500	0.375
S8_24525162	8	2	0.818	0.298	0.253
8_BLOCK_24738259_24738292	8	2	0.545	0.496	0.373
8_BLOCK_24960175_25156978	8	2	0.606	0.478	0.363
8_BLOCK_25217908_25217947	8	2	0.606	0.478	0.363
8_BLOCK_25580745_25652487	8	2	0.848	0.257	0.224
S8_25884960	8	2	0.515	0.500	0.375
S8_25932932	8	2	0.515	0.500	0.375
8_BLOCK_26037233_26233400	8	3	0.485	0.632	0.560
S8_26256196	8	2	0.515	0.500	0.375
S8_26295625	8	2	0.758	0.367	0.300
S8_26328622	8	2	0.545	0.496	0.373
S8_26333573	8	2	0.697	0.422	0.333
8_BLOCK_26387970_26571411	8	3	0.455	0.628	0.551
S8_26604364	8	2	0.545	0.496	0.373
S8_26749559	8	2	0.879	0.213	0.190
8_BLOCK_26796283_26827393	8	3	0.545	0.599	0.532
8_BLOCK_26862470_27057202	8	3	0.455	0.599	0.513
S8_27119735	8	2	0.576	0.489	0.369
S8_27123385	8	2	0.606	0.478	0.363
S8_27168384	8	2	0.758	0.367	0.300
S8_27176044	8	2	0.697	0.422	0.333
8_BLOCK_27176617_27180546	8	2	0.697	0.422	0.333
8_BLOCK_27204498_27400688	8	3	0.424	0.632	0.554
8_BLOCK_27502895_27669522	8	3	0.455	0.628	0.551
S8_27671337	8	2	0.606	0.478	0.363
S8_27671952	8	2	0.818	0.298	0.253
8_BLOCK_27685940_27885469	8	3	0.424	0.617	0.536
8_BLOCK_27905115_28102021	8	3	0.424	0.617	0.536
8_BLOCK_28102044_28122169	8	2	0.545	0.496	0.373
8_BLOCK_28163740_28164360	8	2	0.818	0.298	0.253
8_BLOCK_28177537_28375664	8	3	0.455	0.615	0.535
S8_28399689	8	2	0.515	0.500	0.375
9_BLOCK_248793_344526	8	2	0.636	0.463	0.356

S9_489976	9	2	0.727	0.397	0.318
S9_629325	9	2	0.788	0.334	0.278
S9_629357	9	2	0.636	0.463	0.356
S9_657446	9	2	0.606	0.478	0.363
S9_678193	9	2	0.758	0.367	0.300
9_BLOCK_739068_925951	9	2	0.727	0.397	0.318
9_BLOCK_945588_1092266	9	2	0.727	0.397	0.318
9_BLOCK_1272612_1272682	9	2	0.727	0.397	0.318
S9_1320702	9	2	0.727	0.397	0.318
S9_1577990	9	2	0.727	0.397	0.318
S9_1710535	9	2	0.727	0.397	0.318
S9_1814967	9	2	0.727	0.397	0.318
S9_1862423	9	2	0.727	0.397	0.318
S9_1986875	9	2	0.727	0.397	0.318
S9_2086424	9	2	0.727	0.397	0.318
S9_2335622	9	2	0.727	0.397	0.318
S9_2698548	9	2	0.727	0.397	0.318
9_BLOCK_3063991_3141809	9	2	0.727	0.397	0.318
9_BLOCK_3208563_3370771	9	2	0.727	0.397	0.318
S9_3493434	9	2	0.727	0.397	0.318
9_BLOCK_3565351_3761186	9	2	0.727	0.397	0.318
S9_3866977	9	2	0.727	0.397	0.318
9_BLOCK_4020834_4210235	9	2	0.727	0.397	0.318
S9_4228636	9	2	0.818	0.298	0.253
9_BLOCK_4451412_4597522	9	3	0.515	0.615	0.545
S9_4780587	9	2	0.788	0.334	0.278
S9_4991542	9	2	0.727	0.397	0.318
S9_5020921	9	2	0.667	0.444	0.346
9_BLOCK_5095480_5110929	9	2	0.848	0.257	0.224
9_BLOCK_5211949_5396427	9	3	0.424	0.632	0.554
9_BLOCK_5432371_5432410	9	2	0.606	0.478	0.363
S9_5601109	9	2	0.576	0.489	0.369
S9_5624489	9	2	0.909	0.165	0.152
S9_5809537	9	2	0.606	0.478	0.363
9_BLOCK_5863998_5871928	9	2	0.576	0.489	0.369
S9_5900694	9	2	0.818	0.298	0.253
S9_6017739	9	2	0.667	0.444	0.346
S9_6118798	9	2	0.727	0.397	0.318
S9_6266643	9	2	0.697	0.422	0.333
S9_6325397	9	2	0.576	0.489	0.369

S9_6394711	9	2	0.545	0.496	0.373
9_BLOCK_6583643_6746211	9	2	0.758	0.367	0.300
S9_6793152	9	2	0.606	0.478	0.363
9_BLOCK_6799274_6903110	9	2	0.576	0.489	0.369
S9_7083333	9	2	0.515	0.500	0.375
S9_7278736	9	2	0.515	0.500	0.375
S9_7335019	9	2	0.788	0.334	0.278
9_BLOCK_7350967_7518963	9	2	0.606	0.478	0.363
9_BLOCK_7776410_7776411	9	2	0.515	0.500	0.375
9_BLOCK_7852608_7852647	9	2	0.848	0.257	0.224
S9_8194594	9	2	0.697	0.422	0.333
9_BLOCK_8520088_8608506	9	2	0.727	0.397	0.318
9_BLOCK_8871518_9063468	9	2	0.576	0.489	0.369
9_BLOCK_9112825_9136079	9	2	0.515	0.500	0.375
9_BLOCK_9168442_9341076	9	2	0.545	0.496	0.373
S9_9586169	9	2	0.848	0.257	0.224
9_BLOCK_9857526_9948559	9	3	0.485	0.610	0.531
S9_9974181	9	2	0.576	0.489	0.369
S9_10129882	9	2	0.576	0.489	0.369
S9_10153398	9	2	0.848	0.257	0.224
9_BLOCK_10187530_10325181	9	2	0.545	0.496	0.373
9_BLOCK_10362205_10362325	9	2	0.848	0.257	0.224
9_BLOCK_10363957_10363960	9	2	0.606	0.478	0.363
S9_10772308	9	2	0.636	0.463	0.356
S9_10899300	9	2	0.636	0.463	0.356
9_BLOCK_11020728_11020733	9	2	0.515	0.500	0.375
9_BLOCK_11028674_11028707	9	2	0.879	0.213	0.190
9_BLOCK_11128244_11230987	9	3	0.455	0.643	0.570
S9_11238608	9	2	0.879	0.213	0.190
S9_11265081	9	2	0.697	0.422	0.333
9_BLOCK_11265084_11459537	9	3	0.636	0.522	0.460
9_BLOCK_11459549_11556081	9	2	0.818	0.298	0.253
9_BLOCK_11880937_12080879	9	3	0.455	0.637	0.563
S9_12080951	9	2	0.545	0.496	0.373
S9_12237256	9	2	0.545	0.496	0.373
9_BLOCK_12287469_12474330	9	2	0.545	0.496	0.373
S9_12601832	9	2	0.848	0.257	0.224
9_BLOCK_12636807_12697367	9	2	0.697	0.422	0.333
9_BLOCK_12770477_12963879	9	2	0.606	0.478	0.363
9_BLOCK_12975053_13147265	9	2	0.606	0.478	0.363

S9_13317616	9	2	0.606	0.478	0.363
9_BLOCK_13636111_13786564	9	2	0.606	0.478	0.363
S9_13878665	9	2	0.606	0.478	0.363
S9_14164652	9	2	0.576	0.489	0.369
S9_14359329	9	2	0.879	0.213	0.190
S9_14519362	9	2	0.879	0.213	0.190
9_BLOCK_14599124_14797034	9	3	0.455	0.645	0.572
9_BLOCK_14825884_14854433	9	3	0.455	0.645	0.572
9_BLOCK_14895354_15091551	9	3	0.455	0.645	0.572
9_BLOCK_15135247_15212081	9	3	0.455	0.599	0.513
9_BLOCK_15228311_15426076	9	3	0.455	0.599	0.513
9_BLOCK_15438410_15458640	9	3	0.455	0.599	0.513
9_BLOCK_15489280_15688172	9	2	0.545	0.496	0.373
9_BLOCK_15762553_15959818	9	3	0.455	0.637	0.563
9_BLOCK_16002455_16014831	9	2	0.545	0.496	0.373
9_BLOCK_16144504_16311633	9	3	0.485	0.621	0.546
S9_16362244	9	2	0.758	0.367	0.300
S9_16362265	9	2	0.515	0.500	0.375
9_BLOCK_16389457_16588508	9	2	0.515	0.500	0.375
S9_16614350	9	2	0.848	0.257	0.224
9_BLOCK_16614361_16807906	9	2	0.515	0.500	0.375
9_BLOCK_16824549_16988020	9	2	0.515	0.500	0.375
S9_17108685	9	2	0.606	0.478	0.363
9_BLOCK_17208578_17407032	9	2	0.515	0.500	0.375
S9_17468691	9	2	0.515	0.500	0.375
9_BLOCK_17535393_17676486	9	3	0.485	0.610	0.531
9_BLOCK_17740280_17905429	9	4	0.303	0.735	0.685
9_BLOCK_17912079_18109473	9	3	0.485	0.621	0.546
S9_18161150	9	2	0.576	0.489	0.369
S9_18384640	9	2	0.515	0.500	0.375
9_BLOCK_18494127_18688812	9	2	0.606	0.478	0.363
9_BLOCK_18715972_18722835	9	2	0.576	0.489	0.369
9_BLOCK_18754427_18953808	9	3	0.576	0.571	0.503
9_BLOCK_18980217_19158559	9	3	0.606	0.544	0.476
9_BLOCK_19158571_19220394	9	3	0.606	0.544	0.476
S9_19220395	9	2	0.727	0.397	0.318
S9_19262107	9	2	0.848	0.257	0.224
9_BLOCK_19270192_19428376	9	3	0.606	0.551	0.488
9_BLOCK_19430544_19432064	9	2	0.848	0.257	0.224
9_BLOCK_19575308_19760644	9	3	0.606	0.551	0.488

S9_19760717	9	2	0.788	0.334	0.278
S9_19786664	9	2	0.879	0.213	0.190
9_BLOCK_19806236_19979493	9	3	0.576	0.562	0.489
9_BLOCK_20008740_20203750	9	4	0.576	0.606	0.562
S9_20249643	9	2	0.818	0.298	0.253
9_BLOCK_20278565_20464993	9	3	0.576	0.562	0.489
9_BLOCK_20484265_20562624	9	2	0.788	0.334	0.278
9_BLOCK_20562671_20726155	9	3	0.697	0.468	0.423
S9_20789788	9	2	0.848	0.257	0.224
S9_20789810	9	2	0.848	0.257	0.224
9_BLOCK_20789844_20829514	9	2	0.697	0.422	0.333
9_BLOCK_20844799_20871078	9	3	0.515	0.588	0.506
9_BLOCK_20925868_21081274	9	2	0.515	0.500	0.375
9_BLOCK_21086471_21086508	9	2	0.606	0.478	0.363
S9_21092077	9	2	0.848	0.257	0.224
9_BLOCK_21166188_21364247	9	2	0.515	0.500	0.375
S9_21484884	9	2	0.909	0.165	0.152
S9_21615896	9	2	0.606	0.478	0.363
9_BLOCK_21637627_21821091	9	2	0.515	0.500	0.375
9_BLOCK_21834826_22034801	9	2	0.515	0.500	0.375
9_BLOCK_22059777_22243100	9	2	0.515	0.500	0.375
9_BLOCK_22280642_22303549	9	2	0.515	0.500	0.375
9_BLOCK_22308334_22508230	9	2	0.515	0.500	0.375
9_BLOCK_22511791_22706202	9	2	0.515	0.500	0.375
9_BLOCK_22733274_22925531	9	2	0.515	0.500	0.375
10_BLOCK_46485_95672	10	3	0.455	0.628	0.551
10_BLOCK_102419_299285	10	3	0.455	0.628	0.551
10_BLOCK_426788_526921	10	3	0.455	0.645	0.572
10_BLOCK_589244_788546	10	3	0.455	0.643	0.570
10_BLOCK_798815_928508	10	3	0.424	0.650	0.576
S10_1226529	10	2	0.576	0.489	0.369
10_BLOCK_1469464_1564941	10	2	0.636	0.463	0.356
S10_1565126	10	2	0.788	0.334	0.278
10_BLOCK_1624313_1631865	10	3	0.485	0.628	0.556
S10_1839482	10	2	0.727	0.397	0.318
10_BLOCK_1909296_1909352	10	2	0.727	0.397	0.318
S10_2115860	10	2	0.848	0.257	0.224
S10_2147132	10	2	0.818	0.298	0.253
S10_2151363	10	2	0.545	0.496	0.373
S10_2165343	10	2	0.606	0.478	0.363

10_BLOCK_2314605_2484931	10	3	0.576	0.562	0.489
S10_2484963	10	2	0.515	0.500	0.375
S10_2505181	10	2	0.545	0.496	0.373
S10_2505199	10	2	0.909	0.165	0.152
S10_2718469	10	2	0.545	0.496	0.373
S10_2796551	10	2	0.576	0.489	0.369
10_BLOCK_2847641_3020516	10	2	0.515	0.500	0.375
S10_3090566	10	2	0.636	0.463	0.356
10_BLOCK_3412563_3412614	10	2	0.788	0.334	0.278
S10_3430234	10	2	0.667	0.444	0.346
S10_3501404	10	2	0.879	0.213	0.190
S10_3502190	10	2	0.576	0.489	0.369
S10_3516111	10	2	0.788	0.334	0.278
S10_3793646	10	2	0.727	0.397	0.318
S10_3806349	10	2	0.727	0.397	0.318
S10_3847351	10	2	0.727	0.397	0.318
S10_3847375	10	2	0.818	0.298	0.253
S10_3928990	10	2	0.727	0.397	0.318
S10_3992382	10	2	0.667	0.444	0.346
10_BLOCK_3992402_4082613	10	2	0.576	0.489	0.369
S10_4348182	10	2	0.788	0.334	0.278
S10_4349670	10	2	0.818	0.298	0.253
10_BLOCK_4349686_4417153	10	3	0.455	0.628	0.551
10_BLOCK_4699166_4699184	10	3	0.394	0.661	0.587
S10_4761136	10	2	0.818	0.298	0.253
S10_4783121	10	2	0.606	0.478	0.363
S10_4947977	10	2	0.576	0.489	0.369
10_BLOCK_5164478_5341512	10	3	0.424	0.632	0.554
S10_5341542	10	2	0.545	0.496	0.373
10_BLOCK_5442582_5562381	10	2	0.576	0.489	0.369
S10_5828161	10	2	0.848	0.257	0.224
10_BLOCK_5917275_5929867	10	2	0.606	0.478	0.363
S10_5967116	10	2	0.636	0.463	0.356
10_BLOCK_6017408_6017573	10	2	0.788	0.334	0.278
10_BLOCK_6017610_6214157	10	3	0.394	0.659	0.585
10_BLOCK_6253808_6296013	10	2	0.576	0.489	0.369
S10_6738604	10	2	0.788	0.334	0.278
S10_7017746	10	2	0.879	0.213	0.190
S10_7018473	10	2	0.879	0.213	0.190
S10_7413163	10	2	0.545	0.496	0.373

S10_7441637	10	2	0.879	0.213	0.190
10_BLOCK_7497012_7692820	10	3	0.455	0.628	0.551
10_BLOCK_7702384_7760357	10	2	0.606	0.478	0.363
S10_7950252	10	2	0.545	0.496	0.373
S10_7978592	10	2	0.606	0.478	0.363
S10_8428939	10	2	0.606	0.478	0.363
10_BLOCK_8788796_8892061	10	2	0.606	0.478	0.363
S10_9097952	10	2	0.576	0.489	0.369
S10_9158402	10	2	0.788	0.334	0.278
S10_9234164	10	2	0.545	0.496	0.373
S10_9259556	10	2	0.818	0.298	0.253
S10_9523930	10	2	0.788	0.334	0.278
S10_9523931	10	2	0.818	0.298	0.253
10_BLOCK_9627559_9627587	10	3	0.515	0.588	0.506
10_BLOCK_9668241_9668250	10	2	0.818	0.298	0.253
S10_9670286	10	2	0.667	0.444	0.346
S10_9697329	10	2	0.636	0.463	0.356
S10_9885049	10	2	0.545	0.496	0.373
S10_9897750	10	2	0.788	0.334	0.278
10_BLOCK_9969481_10089562	10	2	0.667	0.444	0.346
S10_10103776	10	2	0.758	0.367	0.300
10_BLOCK_10110977_10304232	10	3	0.394	0.654	0.579
10_BLOCK_10329302_10485970	10	3	0.455	0.615	0.535
10_BLOCK_10531770_10728242	10	2	0.576	0.489	0.369
10_BLOCK_10738884_10751273	10	2	0.576	0.489	0.369
10_BLOCK_11033189_11058596	10	2	0.545	0.496	0.373
S10_11244593	10	2	0.576	0.489	0.369
S10_11317806	10	2	0.545	0.496	0.373
S10_11406924	10	2	0.545	0.496	0.373
10_BLOCK_11592606_11600311	10	2	0.576	0.489	0.369
S10_11851216	10	2	0.879	0.213	0.190
S10_11860166	10	2	0.636	0.463	0.356
S10_11881424	10	2	0.636	0.463	0.356
10_BLOCK_11908061_11908074	10	2	0.848	0.257	0.224
10_BLOCK_12020169_12210939	10	2	0.515	0.500	0.375
10_BLOCK_12338596_12343926	10	2	0.606	0.478	0.363
S10_12662763	10	2	0.515	0.500	0.375
S10_12798477	10	2	0.515	0.500	0.375
10_BLOCK_12931913_13127021	10	3	0.485	0.610	0.531
S10_13127024	10	2	0.818	0.298	0.253

S10_13127084	10	2	0.636	0.463	0.356
S10_13260874	10	2	0.515	0.500	0.375
S10_13353377	10	2	0.515	0.500	0.375
S10_13383994	10	2	0.515	0.500	0.375
S10_13525472	10	2	0.576	0.489	0.369
10_BLOCK_13700509_13861589	10	2	0.515	0.500	0.375
10_BLOCK_13870687_13990579	10	4	0.455	0.681	0.629
10_BLOCK_14066509_14229058	10	2	0.545	0.496	0.373
10_BLOCK_14282689_14482330	10	3	0.455	0.599	0.513
10_BLOCK_14591726_14767448	10	4	0.455	0.681	0.629
S10_14853371	10	2	0.879	0.213	0.190
S10_14853380	10	2	0.879	0.213	0.190
10_BLOCK_14874544_14874728	10	2	0.576	0.489	0.369
10_BLOCK_15007484_15187240	10	3	0.455	0.599	0.513
S10_15214298	10	2	0.545	0.496	0.373
S10_15436846	10	2	0.879	0.213	0.190
10_BLOCK_15462530_15631149	10	3	0.455	0.599	0.513
10_BLOCK_15681032_15692654	10	3	0.455	0.599	0.513
10_BLOCK_15696945_15894896	10	2	0.545	0.496	0.373
10_BLOCK_15900012_15968753	10	3	0.455	0.637	0.563
10_BLOCK_15984379_16174302	10	2	0.545	0.496	0.373
10_BLOCK_16231872_16231896	10	2	0.545	0.496	0.373
10_BLOCK_16256329_16433911	10	3	0.455	0.599	0.513
10_BLOCK_16457852_16652665	10	3	0.455	0.615	0.535
S10_16726886	10	2	0.606	0.478	0.363
10_BLOCK_16836159_16938931	10	3	0.455	0.645	0.572
10_BLOCK_17006312_17186967	10	3	0.455	0.637	0.563
S10_17237839	10	2	0.758	0.367	0.300
S10_17250111	10	2	0.788	0.334	0.278
S10_17258757	10	2	0.697	0.422	0.333
10_BLOCK_17313096_17314532	10	2	0.848	0.257	0.224
10_BLOCK_17320832_17407158	10	4	0.455	0.692	0.644
10_BLOCK_17454775_17653772	10	3	0.485	0.621	0.546
10_BLOCK_17687236_17749574	10	2	0.818	0.298	0.253
10_BLOCK_17876649_18064728	10	2	0.545	0.496	0.373
S10_18076945	10	2	0.576	0.489	0.369
10_BLOCK_18098306_18295343	10	3	0.455	0.599	0.513
S10_18309715	10	2	0.515	0.500	0.375
10_BLOCK_18309716_18509112	10	2	0.545	0.496	0.373
S10_18523886	10	2	0.545	0.496	0.373

10_BLOCK_18659347_18659783	10	2	0.606	0.478	0.363
10_BLOCK_18740946_18879115	10	3	0.485	0.595	0.511
S10_19036076	10	2	0.515	0.500	0.375
S10_19067413	10	2	0.909	0.165	0.152
S10_19193532	10	2	0.879	0.213	0.190
10_BLOCK_19210071_19325960	10	3	0.424	0.617	0.536
10_BLOCK_19390468_19580696	10	2	0.576	0.489	0.369
10_BLOCK_19715859_19847545	10	3	0.394	0.659	0.585
S10_19847547	10	2	0.727	0.397	0.318
10_BLOCK_19879690_20078288	10	3	0.394	0.659	0.585
10_BLOCK_20115143_20261451	10	3	0.394	0.654	0.579
10_BLOCK_20301106_20488702	10	2	0.606	0.478	0.363
10_BLOCK_20538805_20731579	10	2	0.606	0.478	0.363
S10_20783380	10	2	0.606	0.478	0.363
10_BLOCK_20809027_21004805	10	2	0.545	0.496	0.373
10_BLOCK_21023206_21222981	10	2	0.606	0.478	0.363
S10_21226019	10	2	0.697	0.422	0.333
S10_21248068	10	2	0.697	0.422	0.333
10_BLOCK_21251260_21449027	10	3	0.394	0.659	0.585
10_BLOCK_21454980_21544296	10	3	0.394	0.659	0.585
10_BLOCK_21579941_21770013	10	2	0.606	0.478	0.363
S10_21803569	10	2	0.576	0.489	0.369
10_BLOCK_21816410_22013222	10	2	0.758	0.367	0.300
10_BLOCK_22016646_22044461	10	2	0.758	0.367	0.300
10_BLOCK_22070293_22270204	10	4	0.515	0.646	0.596
S10_22321022	10	2	0.636	0.463	0.356
10_BLOCK_22373037_22523770	10	3	0.485	0.610	0.531
10_BLOCK_22557864_22744020	10	2	0.667	0.444	0.346
10_BLOCK_23014068_23184485	10	2	0.758	0.367	0.300
11_BLOCK_156051_160767	11	2	0.697	0.422	0.333
S11_317209	11	2	0.697	0.422	0.333
11_BLOCK_422416_439071	11	2	0.879	0.213	0.190
11_BLOCK_558540_726259	11	2	0.818	0.298	0.253
11_BLOCK_750967_853188	11	2	0.515	0.500	0.375
11_BLOCK_858441_1053492	11	2	0.515	0.500	0.375
11_BLOCK_1125501_1126003	11	2	0.515	0.500	0.375
11_BLOCK_1166376_1360704	11	2	0.545	0.496	0.373
S11_1400212	11	2	0.545	0.496	0.373
11_BLOCK_1430880_1618103	11	2	0.545	0.496	0.373
S11_1775483	11	2	0.545	0.496	0.373

11_BLOCK_1868332_2056063	11	4	0.455	0.681	0.629
S11_2074647	11	2	0.697	0.422	0.333
S11_2074650	11	2	0.758	0.367	0.300
11_BLOCK_2255547_2426246	11	4	0.455	0.687	0.636
S11_2435427	11	2	0.879	0.213	0.190
S11_2464998	11	2	0.515	0.500	0.375
11_BLOCK_2490872_2556879	11	3	0.455	0.643	0.570
11_BLOCK_2556880_2556884	11	2	0.758	0.367	0.300
11_BLOCK_2556887_2743975	11	3	0.515	0.617	0.548
S11_2955469	11	2	0.879	0.213	0.190
11_BLOCK_3027015_3028240	11	2	0.758	0.367	0.300
S11_3054458	11	2	0.758	0.367	0.300
S11_3096683	11	2	0.879	0.213	0.190
11_BLOCK_3099034_3266976	11	4	0.455	0.687	0.636
S11_3311540	11	2	0.545	0.496	0.373
S11_3585577	11	2	0.788	0.334	0.278
11_BLOCK_3585600_3673599	11	3	0.455	0.628	0.551
S11_3714394	11	2	0.758	0.367	0.300
11_BLOCK_3714400_3765793	11	3	0.515	0.617	0.548
11_BLOCK_3776875_3851210	11	3	0.455	0.615	0.535
S11_3857756	11	2	0.727	0.397	0.318
S11_3858444	11	2	0.545	0.496	0.373
11_BLOCK_4083700_4084491	11	2	0.545	0.496	0.373
11_BLOCK_4132702_4325428	11	2	0.545	0.496	0.373
S11_4402216	11	2	0.515	0.500	0.375
11_BLOCK_4402235_4429583	11	5	0.303	0.781	0.746
S11_4445141	11	2	0.848	0.257	0.224
S11_4445195	11	2	0.727	0.397	0.318
11_BLOCK_4451034_4526609	11	3	0.515	0.588	0.506
11_BLOCK_4564443_4754088	11	3	0.424	0.654	0.580
S11_4787370	11	2	0.576	0.489	0.369
S11_4807369	11	2	0.636	0.463	0.356
11_BLOCK_4807408_4994476	11	4	0.333	0.729	0.679
11_BLOCK_5069825_5137900	11	3	0.485	0.628	0.556
11_BLOCK_5383281_5386854	11	2	0.576	0.489	0.369
11_BLOCK_5442188_5589735	11	3	0.576	0.579	0.515
11_BLOCK_5625611_5809104	11	3	0.424	0.632	0.554
11_BLOCK_5852938_5869438	11	2	0.576	0.489	0.369
S11_5918620	11	2	0.758	0.367	0.300
11_BLOCK_5918662_6105083	11	3	0.545	0.599	0.532

11_BLOCK_6205655_6382821	11	2	0.515	0.500	0.375
11_BLOCK_6649884_6707091	11	3	0.485	0.595	0.511
11_BLOCK_6934633_7106764	11	4	0.303	0.735	0.685
S11_7106767	11	2	0.879	0.213	0.190
S11_7194365	11	2	0.879	0.213	0.190
S11_7194368	11	2	0.697	0.422	0.333
S11_7487225	11	2	0.758	0.367	0.300
11_BLOCK_7568617_7570762	11	2	0.606	0.478	0.363
S11_7603154	11	2	0.545	0.496	0.373
11_BLOCK_7630915_7821282	11	2	0.545	0.496	0.373
11_BLOCK_7985757_8036822	11	2	0.545	0.496	0.373
11_BLOCK_8084484_8275155	11	3	0.455	0.615	0.535
11_BLOCK_8323714_8323732	11	2	0.606	0.478	0.363
S11_8388165	11	2	0.848	0.257	0.224
11_BLOCK_8553901_8702585	11	2	0.545	0.496	0.373
S11_8993081	11	2	0.545	0.496	0.373
S11_9004341	11	2	0.667	0.444	0.346
11_BLOCK_9066216_9214449	11	2	0.545	0.496	0.373
11_BLOCK_9214528_9323354	11	3	0.455	0.599	0.513
11_BLOCK_9323355_9329741	11	2	0.606	0.478	0.363
S11_9686091	11	2	0.667	0.444	0.346
S11_9803381	11	2	0.788	0.334	0.278
11_BLOCK_9852004_9957335	11	2	0.545	0.496	0.373
11_BLOCK_10057175_10253867	11	3	0.455	0.628	0.551
11_BLOCK_10262474_10354126	11	4	0.455	0.687	0.636
S11_10354132	11	2	0.758	0.367	0.300
11_BLOCK_10354136_10354144	11	2	0.545	0.496	0.373
S11_10582375	11	2	0.545	0.496	0.373
S11_10609357	11	2	0.545	0.496	0.373
S11_10688307	11	2	0.545	0.496	0.373
S11_10717985	11	2	0.758	0.367	0.300
S11_10824142	11	2	0.545	0.496	0.373
11_BLOCK_10912910_11096460	11	3	0.545	0.588	0.515
11_BLOCK_11147860_11336565	11	3	0.545	0.588	0.515
11_BLOCK_11382906_11436523	11	3	0.545	0.588	0.515
11_BLOCK_11566351_11765666	11	2	0.545	0.496	0.373
S11_11783530	11	2	0.848	0.257	0.224
11_BLOCK_11783543_11981216	11	4	0.364	0.720	0.669
S11_12077123	11	2	0.697	0.422	0.333
S11_12612263	11	2	0.697	0.422	0.333

S11_12809008	11	2	0.545	0.496	0.373
S11_12865885	11	2	0.545	0.496	0.373
S11_13253982	11	2	0.758	0.367	0.300
S11_13253989	11	2	0.545	0.496	0.373
11_BLOCK_13466576_13466577	11	2	0.545	0.496	0.373
S11_13560432	11	2	0.697	0.422	0.333
S11_13674694	11	2	0.545	0.496	0.373
11_BLOCK_13724315_13724328	11	2	0.848	0.257	0.224
11_BLOCK_13784931_13978111	11	3	0.545	0.577	0.499
S11_14052029	11	2	0.788	0.334	0.278
11_BLOCK_14118290_14118965	11	2	0.818	0.298	0.253
S11_14169508	11	2	0.848	0.257	0.224
S11_14314411	11	2	0.788	0.334	0.278
S11_14555418	11	2	0.788	0.334	0.278
11_BLOCK_14555436_14606851	11	3	0.606	0.551	0.488
11_BLOCK_14628594_14823390	11	4	0.424	0.691	0.636
S11_14860445	11	2	0.576	0.489	0.369
S11_14962328	11	2	0.576	0.489	0.369
S11_15119012	11	2	0.848	0.257	0.224
11_BLOCK_15309810_15457242	11	3	0.545	0.599	0.532
S11_15460315	11	2	0.515	0.500	0.375
S11_15462589	11	2	0.788	0.334	0.278
11_BLOCK_15694616_15755896	11	3	0.455	0.643	0.570
11_BLOCK_15768008_15904813	11	3	0.455	0.643	0.570
S11_16200411	11	2	0.758	0.367	0.300
11_BLOCK_16200772_16397090	11	4	0.424	0.705	0.656
S11_16448317	11	2	0.576	0.489	0.369
S11_16510199	11	2	0.818	0.298	0.253
11_BLOCK_16511045_16710899	11	3	0.424	0.632	0.554
11_BLOCK_16710912_16770852	11	3	0.455	0.615	0.535
S11_16779579	11	2	0.818	0.298	0.253
11_BLOCK_16864000_16951755	11	4	0.455	0.681	0.629
11_BLOCK_17130489_17181463	11	2	0.848	0.257	0.224
11_BLOCK_17292444_17382241	11	2	0.576	0.489	0.369
11_BLOCK_17417544_17614894	11	3	0.424	0.632	0.554
11_BLOCK_17619458_17750430	11	3	0.424	0.643	0.567
S11_17819934	11	2	0.606	0.478	0.363
11_BLOCK_17820001_17945021	11	4	0.545	0.628	0.581
11_BLOCK_18063454_18076335	11	3	0.576	0.562	0.489
S11_18101826	11	2	0.727	0.397	0.318

11_BLOCK_18121821_18288410	11	3	0.727	0.433	0.393
S11_18314461	11	2	0.788	0.334	0.278
S11_18314466	11	2	0.879	0.213	0.190
S11_18363347	11	2	0.667	0.444	0.346
S11_18386955	11	2	0.848	0.257	0.224
S11_18413569	11	2	0.848	0.257	0.224
11_BLOCK_18431697_18435610	11	3	0.545	0.577	0.499
S11_18435612	11	2	0.818	0.298	0.253
S11_18435615	11	2	0.818	0.298	0.253
S11_18435636	11	2	0.818	0.298	0.253
S11_18490507	11	2	0.879	0.213	0.190
S11_18531896	11	2	0.879	0.213	0.190
S11_18625126	11	2	0.879	0.213	0.190
S11_18732403	11	2	0.909	0.165	0.152
S11_18810175	11	2	0.545	0.496	0.373
S11_18818238	11	2	0.727	0.397	0.318
11_BLOCK_18901416_19072571	11	4	0.485	0.669	0.618
S11_19227697	11	2	0.576	0.489	0.369
S11_19263107	11	2	0.909	0.165	0.152
11_BLOCK_19263139_19263152	11	2	0.727	0.397	0.318
S11_19309293	11	2	0.788	0.334	0.278
11_BLOCK_19309311_19309329	11	2	0.697	0.422	0.333
11_BLOCK_19355277_19542991	11	4	0.424	0.709	0.661
S11_19607658	11	2	0.727	0.397	0.318
S11_19636808	11	2	0.697	0.422	0.333
S11_19707581	11	2	0.879	0.213	0.190
S11_19707591	11	2	0.879	0.213	0.190
11_BLOCK_19709834_19709850	11	2	0.667	0.444	0.346
S11_19875835	11	2	0.515	0.500	0.375
S11_19926628	11	2	0.879	0.213	0.190
11_BLOCK_20029670_20036719	11	4	0.333	0.740	0.692
S11_20036735	11	2	0.667	0.444	0.346
S11_20036741	11	2	0.758	0.367	0.300
S11_20101765	11	2	0.667	0.444	0.346
S11_20171573	11	2	0.909	0.165	0.152
S11_20176606	11	2	0.727	0.397	0.318
11_BLOCK_20176616_20356516	11	4	0.333	0.736	0.688
11_BLOCK_20480700_20564349	11	3	0.424	0.643	0.567
S11_20644161	11	2	0.636	0.463	0.356
S11_20742520	11	2	0.667	0.444	0.346

S11_20746698	11	2	0.667	0.444	0.346
S11_20799527	11	2	0.758	0.367	0.300
11_BLOCK_20799553_20985157	11	3	0.394	0.659	0.585
S11_21040172	11	2	0.606	0.478	0.363
S11_21215473	11	2	0.818	0.298	0.253
11_BLOCK_21235159_21382794	11	2	0.576	0.489	0.369
11_BLOCK_21463003_21485911	11	3	0.485	0.621	0.546
11_BLOCK_21495207_21687706	11	2	0.515	0.500	0.375
S11_21691103	11	2	0.848	0.257	0.224
11_BLOCK_21789767_21795488	11	3	0.485	0.595	0.511
11_BLOCK_21823911_22012543	11	3	0.394	0.659	0.585
S11_22031762	11	2	0.606	0.478	0.363
S11_22064972	11	2	0.515	0.500	0.375
S11_22130966	11	2	0.909	0.165	0.152
11_BLOCK_22256189_22256198	11	2	0.576	0.489	0.369
11_BLOCK_22266277_22278397	11	2	0.606	0.478	0.363
S11_22278440	11	2	0.848	0.257	0.224
S11_22302306	11	2	0.697	0.422	0.333
S11_22463659	11	2	0.667	0.444	0.346
S11_22463660	11	2	0.818	0.298	0.253
S11_22463680	11	2	0.636	0.463	0.356
S11_22476195	11	2	0.879	0.213	0.190
11_BLOCK_22513889_22659856	11	4	0.364	0.727	0.678
11_BLOCK_22661149_22848601	11	4	0.303	0.736	0.687
11_BLOCK_22884532_22884541	11	2	0.697	0.422	0.333
11_BLOCK_22899060_23033776	11	4	0.333	0.738	0.690
S11_23069712	11	2	0.606	0.478	0.363
S11_23179607	11	2	0.879	0.213	0.190
11_BLOCK_23190863_23358199	11	3	0.485	0.595	0.511
11_BLOCK_23378612_23378628	11	2	0.667	0.444	0.346
S11_23422768	11	2	0.697	0.422	0.333
11_BLOCK_23565814_23565855	11	3	0.667	0.500	0.448
11_BLOCK_23750643_23750756	11	2	0.636	0.463	0.356
S11_23861735	11	2	0.758	0.367	0.300
11_BLOCK_23861754_23986595	11	4	0.303	0.746	0.698
S11_23986619	11	2	0.818	0.298	0.253
S11_23986631	11	2	0.818	0.298	0.253
S11_24102991	11	2	0.636	0.463	0.356
S11_24102994	11	2	0.758	0.367	0.300
S11_24222650	11	2	0.758	0.367	0.300

S11_24264059	11	2	0.545	0.496	0.373
11_BLOCK_24319508_24340191	11	3	0.394	0.659	0.585
S11_24349429	11	2	0.818	0.298	0.253
11_BLOCK_24442254_24636131	11	3	0.545	0.595	0.526
11_BLOCK_24639193_24639195	11	2	0.576	0.489	0.369
S11_24824536	11	2	0.818	0.298	0.253
S11_24824538	11	2	0.697	0.422	0.333
S11_24839099	11	2	0.848	0.257	0.224
11_BLOCK_24934768_24934773	11	2	0.818	0.298	0.253
S11_24971235	11	2	0.606	0.478	0.363
S11_25044919	11	2	0.848	0.257	0.224
S11_25137188	11	2	0.697	0.422	0.333
S11_25138789	11	2	0.727	0.397	0.318
11_BLOCK_25138881_25138887	11	2	0.788	0.334	0.278
S11_25214331	11	2	0.667	0.444	0.346
S11_25239221	11	2	0.697	0.422	0.333
S11_25435421	11	2	0.545	0.496	0.373
11_BLOCK_25467677_25502067	11	2	0.758	0.367	0.300
11_BLOCK_25542769_25578556	11	4	0.394	0.714	0.664
S11_25578562	11	2	0.636	0.463	0.356
11_BLOCK_25578595_25605743	11	2	0.576	0.489	0.369
11_BLOCK_25606324_25773649	11	2	0.818	0.298	0.253
S11_25773661	11	2	0.788	0.334	0.278
S11_25821693	11	2	0.788	0.334	0.278
S11_25839380	11	2	0.727	0.397	0.318
S11_25854146	11	2	0.788	0.334	0.278
S11_25890875	11	2	0.697	0.422	0.333
11_BLOCK_26029941_26213611	11	4	0.394	0.714	0.664
S11_26218645	11	2	0.727	0.397	0.318
11_BLOCK_26219551_26261114	11	2	0.636	0.463	0.356
11_BLOCK_26261366_26261369	11	2	0.515	0.500	0.375
S11_26261395	11	2	0.818	0.298	0.253
S11_26273266	11	2	0.576	0.489	0.369
S11_26376645	11	2	0.606	0.478	0.363
S11_26472423	11	2	0.667	0.444	0.346
11_BLOCK_26529099_26529140	11	3	0.485	0.628	0.556
S11_26797495	11	2	0.788	0.334	0.278
S11_26799179	11	2	0.818	0.298	0.253
S11_26812502	11	2	0.848	0.257	0.224
S11_26878717	11	2	0.515	0.500	0.375

S11_26878737	11	2	0.727	0.397	0.318
11_BLOCK_26916905_27078112	11	4	0.424	0.707	0.658
11_BLOCK_27149504_27177944	11	3	0.515	0.588	0.506
S11_27204539	11	2	0.545	0.496	0.373
S11_27282373	11	2	0.788	0.334	0.278
S11_27573844	11	2	0.758	0.367	0.300
S11_27573854	11	2	0.636	0.463	0.356
11_BLOCK_27581508_27581518	11	2	0.848	0.257	0.224
S11_27581519	11	2	0.636	0.463	0.356
11_BLOCK_27581521_27581527	11	3	0.485	0.632	0.560
S11_27660814	11	2	0.788	0.334	0.278
11_BLOCK_27692769_27692798	11	2	0.788	0.334	0.278
11_BLOCK_27793983_27794015	11	2	0.879	0.213	0.190
11_BLOCK_27804861_27804887	11	2	0.758	0.367	0.300
11_BLOCK_27812941_27812964	11	2	0.879	0.213	0.190
S11_27817978	11	2	0.727	0.397	0.318
S11_27829598	11	2	0.606	0.478	0.363
S11_27829621	11	2	0.909	0.165	0.152
S11_27829638	11	2	0.909	0.165	0.152
S11_27831269	11	2	0.727	0.397	0.318
11_BLOCK_27831419_27831437	11	2	0.818	0.298	0.253
S11_28004835	11	2	0.697	0.422	0.333
S11_28005681	11	2	0.606	0.478	0.363
S11_28005704	11	2	0.818	0.298	0.253
S11_28437774	11	2	0.727	0.397	0.318
11_BLOCK_28652054_28715756	11	2	0.697	0.422	0.333
S11_28748848	11	2	0.879	0.213	0.190
S11_28763056	11	2	0.667	0.444	0.346
S11_28763081	11	2	0.879	0.213	0.190
S11_28785923	11	2	0.818	0.298	0.253
S11_28789197	11	2	0.848	0.257	0.224
S11_28797088	11	2	0.848	0.257	0.224
11_BLOCK_28808840_28808879	11	3	0.364	0.665	0.591
11_BLOCK_28871662_28989817	11	3	0.485	0.628	0.556
12_BLOCK_124195_227740	12	3	0.485	0.610	0.531
S12_469155	12	2	0.606	0.478	0.363
S12_469535	12	2	0.818	0.298	0.253
12_BLOCK_659037_839035	12	3	0.424	0.632	0.554
S12_953349	12	2	0.545	0.496	0.373
S12_1027062	12	2	0.545	0.496	0.373

12_BLOCK_1158035_1158042	12	2	0.879	0.213	0.190
S12_1319144	12	2	0.879	0.213	0.190
12_BLOCK_1336421_1515198	12	2	0.515	0.500	0.375
12_BLOCK_1660440_1691823	12	2	0.576	0.489	0.369
12_BLOCK_1752449_1938225	12	2	0.576	0.489	0.369
S12_1974147	12	2	0.576	0.489	0.369
S12_1998100	12	2	0.576	0.489	0.369
S12_2142425	12	2	0.576	0.489	0.369
S12_2176201	12	2	0.576	0.489	0.369
S12_2227916	12	2	0.576	0.489	0.369
12_BLOCK_2252898_2447969	12	2	0.576	0.489	0.369
12_BLOCK_2455671_2458830	12	2	0.576	0.489	0.369
S12_2872032	12	2	0.879	0.213	0.190
S12_2946686	12	2	0.909	0.165	0.152
S12_2948477	12	2	0.909	0.165	0.152
S12_2975941	12	2	0.909	0.165	0.152
S12_2975971	12	2	0.909	0.165	0.152
S12_3033666	12	2	0.879	0.213	0.190
S12_3119929	12	2	0.879	0.213	0.190
S12_3148965	12	2	0.727	0.397	0.318
S12_3180750	12	2	0.879	0.213	0.190
S12_3209332	12	2	0.879	0.213	0.190
12_BLOCK_3214955_3413848	12	3	0.485	0.595	0.511
12_BLOCK_3413851_3600003	12	2	0.515	0.500	0.375
S12_3650166	12	2	0.515	0.500	0.375
S12_3682470	12	2	0.788	0.334	0.278
12_BLOCK_3744485_3884843	12	2	0.515	0.500	0.375
12_BLOCK_3963103_4104325	12	2	0.515	0.500	0.375
S12_4146672	12	2	0.879	0.213	0.190
S12_4199682	12	2	0.606	0.478	0.363
12_BLOCK_4216046_4216147	12	2	0.788	0.334	0.278
12_BLOCK_4499453_4591247	12	2	0.515	0.500	0.375
12_BLOCK_4900237_5082848	12	2	0.515	0.500	0.375
12_BLOCK_5381325_5457883	12	2	0.606	0.478	0.363
12_BLOCK_5589779_5776355	12	3	0.394	0.645	0.569
12_BLOCK_6138072_6138113	12	2	0.667	0.444	0.346
S12_6584008	12	2	0.818	0.298	0.253
12_BLOCK_6717439_6717453	12	2	0.667	0.444	0.346
12_BLOCK_6733428_6870968	12	4	0.515	0.654	0.607
12_BLOCK_7021618_7204973	12	2	0.606	0.478	0.363

12_BLOCK_7222770_7420337	12	4	0.394	0.707	0.655
S12_7434458	12	2	0.758	0.367	0.300
12_BLOCK_7503930_7561908	12	2	0.606	0.478	0.363
12_BLOCK_7812791_7918400	12	2	0.606	0.478	0.363
S12_8026679	12	2	0.606	0.478	0.363
S12_8035728	12	2	0.879	0.213	0.190
S12_8035737	12	2	0.879	0.213	0.190
12_BLOCK_8036070_8183497	12	3	0.485	0.595	0.511
S12_8695968	12	2	0.606	0.478	0.363
S12_8874326	12	2	0.606	0.478	0.363
12_BLOCK_8961173_9126472	12	3	0.394	0.645	0.569
S12_9180371	12	2	0.606	0.478	0.363
S12_9229559	12	2	0.576	0.489	0.369
12_BLOCK_9229569_9229576	12	2	0.818	0.298	0.253
S12_9478016	12	2	0.758	0.367	0.300
S12_9478546	12	2	0.576	0.489	0.369
12_BLOCK_9491041_9499921	12	2	0.848	0.257	0.224
S12_9570099	12	2	0.758	0.367	0.300
12_BLOCK_9613731_9788284	12	4	0.424	0.705	0.656
12_BLOCK_9896211_9896221	12	2	0.758	0.367	0.300
12_BLOCK_10085866_10085913	12	2	0.576	0.489	0.369
12_BLOCK_10106776_10210885	12	3	0.606	0.551	0.488
S12_10255733	12	2	0.848	0.257	0.224
S12_10255774	12	2	0.818	0.298	0.253
S12_10274260	12	2	0.848	0.257	0.224
12_BLOCK_10364946_10364985	12	2	0.848	0.257	0.224
12_BLOCK_10611754_10652708	12	4	0.455	0.689	0.639
S12_10652724	12	2	0.848	0.257	0.224
12_BLOCK_10698110_10872869	12	4	0.424	0.700	0.649
12_BLOCK_10901320_10901329	12	2	0.848	0.257	0.224
S12_10904513	12	2	0.576	0.489	0.369
S12_10996127	12	2	0.848	0.257	0.224
12_BLOCK_11127130_11133685	12	2	0.848	0.257	0.224
S12_11140415	12	2	0.697	0.422	0.333
S12_11205242	12	2	0.758	0.367	0.300
S12_11250179	12	2	0.576	0.489	0.369
S12_11277279	12	2	0.879	0.213	0.190
S12_11735537	12	2	0.879	0.213	0.190
S12_11798751	12	2	0.667	0.444	0.346
S12_12075553	12	2	0.879	0.213	0.190

S12_12075592	12	2	0.879	0.213	0.190
S12_12079083	12	2	0.697	0.422	0.333
S12_12244170	12	2	0.758	0.367	0.300
S12_12257799	12	2	0.667	0.444	0.346
S12_12303559	12	2	0.879	0.213	0.190
S12_12415365	12	2	0.879	0.213	0.190
S12_12430501	12	2	0.848	0.257	0.224
S12_12430520	12	2	0.879	0.213	0.190
S12_12764533	12	2	0.697	0.422	0.333
S12_12797009	12	2	0.848	0.257	0.224
S12_12797013	12	2	0.576	0.489	0.369
12_BLOCK_12967864_13140942	12	4	0.303	0.727	0.676
S12_13173461	12	2	0.697	0.422	0.333
12_BLOCK_13178723_13190461	12	2	0.697	0.422	0.333
S12_13190499	12	2	0.818	0.298	0.253
12_BLOCK_13449169_13540737	12	3	0.485	0.628	0.556
S12_13611619	12	2	0.758	0.367	0.300
12_BLOCK_13663061_13861944	12	3	0.485	0.628	0.556
S12_13879225	12	2	0.879	0.213	0.190
S12_13885659	12	2	0.758	0.367	0.300
S12_13914348	12	2	0.788	0.334	0.278
S12_13915685	12	2	0.636	0.463	0.356
12_BLOCK_13997092_14180506	12	3	0.455	0.637	0.563
12_BLOCK_14219148_14220620	12	2	0.788	0.334	0.278
S12_14220621	12	2	0.545	0.496	0.373
12_BLOCK_14433687_14627294	12	3	0.394	0.659	0.585
12_BLOCK_14665324_14675306	12	2	0.697	0.422	0.333
12_BLOCK_14675317_14684759	12	3	0.424	0.650	0.576
12_BLOCK_14684767_14684776	12	2	0.758	0.367	0.300
12_BLOCK_14748333_14941089	12	3	0.394	0.659	0.585
S12_14954848	12	2	0.758	0.367	0.300
S12_15134595	12	2	0.697	0.422	0.333
S12_15179069	12	2	0.758	0.367	0.300
12_BLOCK_15246376_15423919	12	3	0.424	0.643	0.567
S12_15433199	12	2	0.879	0.213	0.190
S12_15433200	12	2	0.879	0.213	0.190
12_BLOCK_15459159_15459163	12	2	0.667	0.444	0.346
S12_15462024	12	2	0.879	0.213	0.190
12_BLOCK_15779533_15962782	12	2	0.545	0.496	0.373
12_BLOCK_15986984_16168174	12	3	0.455	0.637	0.563

12_BLOCK_16289635_16289710	12	3	0.485	0.595	0.511
12_BLOCK_16347543_16465249	12	3	0.424	0.632	0.554
12_BLOCK_16507330_16706204	12	3	0.606	0.544	0.476
12_BLOCK_16993438_16993457	12	2	0.576	0.489	0.369
S12_17021871	12	2	0.848	0.257	0.224
S12_17073823	12	2	0.879	0.213	0.190
S12_17197741	12	2	0.758	0.367	0.300
12_BLOCK_17312701_17353624	12	3	0.545	0.588	0.515
S12_17478598	12	2	0.576	0.489	0.369
12_BLOCK_17546417_17610813	12	3	0.485	0.595	0.511
12_BLOCK_17625671_17779917	12	4	0.485	0.669	0.618
12_BLOCK_17782389_17782470	12	3	0.606	0.551	0.488
12_BLOCK_18060799_18171316	12	3	0.485	0.610	0.531
12_BLOCK_18514027_18514081	12	2	0.545	0.496	0.373
12_BLOCK_18717643_18913133	12	2	0.515	0.500	0.375
12_BLOCK_19092702_19106374	12	2	0.515	0.500	0.375
12_BLOCK_19106376_19183058	12	3	0.515	0.588	0.506
12_BLOCK_19301094_19378024	12	3	0.667	0.496	0.441
S12_19378055	12	2	0.697	0.422	0.333
S12_19378057	12	2	0.818	0.298	0.253
12_BLOCK_19392454_19392459	12	3	0.485	0.595	0.511
S12_19392469	12	2	0.879	0.213	0.190
S12_19392470	12	2	0.879	0.213	0.190
S12_19396387	12	2	0.727	0.397	0.318
S12_19453096	12	2	0.818	0.298	0.253
S12_19537089	12	2	0.606	0.478	0.363
S12_19537102	12	2	0.576	0.489	0.369
S12_19608008	12	2	0.636	0.463	0.356
12_BLOCK_19663756_19862387	12	3	0.515	0.617	0.548
12_BLOCK_19892955_19895558	12	2	0.606	0.478	0.363
S12_19917539	12	2	0.848	0.257	0.224
12_BLOCK_19991579_20008889	12	2	0.545	0.496	0.373
12_BLOCK_20086502_20086511	12	2	0.788	0.334	0.278
12_BLOCK_20729984_20802707	12	2	0.515	0.500	0.375
12_BLOCK_21021854_21030823	12	2	0.545	0.496	0.373
S12_21488077	12	2	0.879	0.213	0.190
S12_21692001	12	2	0.758	0.367	0.300
12_BLOCK_21772484_21801623	12	2	0.697	0.422	0.333
12_BLOCK_21830076_22029174	12	2	0.545	0.496	0.373
12_BLOCK_22138191_22172441	12	2	0.515	0.500	0.375

12_BLOCK_22192187_22392149	12	3	0.485	0.621	0.546
12_BLOCK_22479868_22591686	12	3	0.515	0.610	0.537
12_BLOCK_22628802_22628856	12	2	0.848	0.257	0.224
S12_22670945	12	2	0.515	0.500	0.375
12_BLOCK_22857742_23007055	12	4	0.485	0.669	0.618
12_BLOCK_23007089_23173265	12	4	0.515	0.646	0.596
S12_23249911	12	2	0.727	0.397	0.318
12_BLOCK_23272380_23460051	12	3	0.515	0.601	0.524
12_BLOCK_23480845_23623637	12	3	0.424	0.654	0.580
S12_23663878	12	2	0.636	0.463	0.356
S12_23663893	12	2	0.758	0.367	0.300
S12_23711644	12	2	0.758	0.367	0.300
S12_23711678	12	2	0.667	0.444	0.346
12_BLOCK_23805060_23805090	12	2	0.576	0.489	0.369
S12_23806388	12	2	0.818	0.298	0.253
12_BLOCK_23823020_23980991	12	2	0.545	0.496	0.373
S12_24054067	12	2	0.697	0.422	0.333
S12_24054126	12	2	0.758	0.367	0.300
S12_24318577	12	2	0.879	0.213	0.190
S12_24349772	12	2	0.636	0.463	0.356
S12_24380437	12	2	0.667	0.444	0.346
S12_24417484	12	2	0.848	0.257	0.224
S12_24450369	12	2	0.758	0.367	0.300
S12_24457241	12	2	0.879	0.213	0.190
12_BLOCK_24485830_24485832	12	2	0.515	0.500	0.375
12_BLOCK_24520989_24716741	12	3	0.485	0.610	0.531
12_BLOCK_24722643_24821303	12	2	0.848	0.257	0.224
12_BLOCK_25058516_25142846	12	2	0.545	0.496	0.373
S12_25308018	12	2	0.788	0.334	0.278
S12_25325902	12	2	0.545	0.496	0.373
S12_25730140	12	2	0.697	0.422	0.333
S12_25764706	12	2	0.909	0.165	0.152
S12_25764740	12	2	0.697	0.422	0.333
12_BLOCK_25780544_25979966	12	2	0.515	0.500	0.375
S12_25986925	12	2	0.515	0.500	0.375
12_BLOCK_26004684_26200309	12	2	0.515	0.500	0.375
12_BLOCK_26204775_26213179	12	2	0.515	0.500	0.375
12_BLOCK_26273123_26372176	12	2	0.788	0.334	0.278
S12_26418121	12	2	0.909	0.165	0.152
S12_26418163	12	2	0.909	0.165	0.152

12_BLOCK_26449118_26631100	12	2	0.727	0.397	0.318
S12_26703212	12	2	0.667	0.444	0.346
S12_26771751	12	2	0.818	0.298	0.253
S12_26771753	12	2	0.667	0.444	0.346
S12_26897395	12	2	0.667	0.444	0.346
S12_27041522	12	2	0.879	0.213	0.190
S12_27041548	12	2	0.879	0.213	0.190
12_BLOCK_27093900_27125673	12	2	0.636	0.463	0.356
S12_27210040	12	2	0.667	0.444	0.346
S12_27238229	12	2	0.667	0.444	0.346
S12_27241143	12	2	0.758	0.367	0.300
S12_27267561	12	2	0.697	0.422	0.333
S12_27317181	12	2	0.515	0.500	0.375
S12_27317214	12	2	0.879	0.213	0.190
S12_6584008	12	3	0.485	0.632	0.560
12_BLOCK_6717439_6717453	12	3	0.485	0.610	0.531
12_BLOCK_6733428_6870968	12	2	0.606	0.478	0.363
12_BLOCK_7021618_7204973	12	2	0.818	0.298	0.253
12_BLOCK_7222770_7420337	12	3	0.424	0.632	0.554
S12_7434458	12	2	0.545	0.496	0.373
12_BLOCK_27321355_27430607	12	2	0.545	0.496	0.373

Supporting Information Table S3- The developed 48 indica and 78 japonica crosses and their mean phenotypic performances for 11 yield-related traits

Indica Combinations	DH	PH	NPPP	NSPP	NFGPP	PL	SW	ST	SL	TGW	GYPP
256A×Minghui 63	98.7	112.8	17.7	147.1	135.7	26.1	2.8	1.9	10.0	30.8	1527.0
256A×Zhenhui 084	96.0	107.0	16.1	216.9	197.5	24.5	2.6	1.9	9.0	32.8	1337.3
256A×Yanhui 559	99.0	111.9	16.4	261.3	233.9	27.4	2.6	2.0	9.6	28.3	1432.3
256A×Huizi 04	100.3	109.3	16.0	154.9	141.0	22.0	2.4	1.9	9.3	30.7	1401.0
256A×Hui 9368	99.3	113.7	16.1	125.3	112.3	19.9	3.1	1.8	7.6	27.7	1218.0
256A×Kanghui98	92.0	118.4	15.3	134.6	118.5	22.4	3.1	2.1	8.0	26.0	1576.3
Zhenpin A×Minghui 63	102.7	113.7	12.5	199.4	177.8	26.0	2.6	2.0	8.2	28.2	1831.3
Zhenpin A×Zhenhui 084	92.3	80.2	13.0	153.9	135.2	25.5	2.3	1.9	9.3	25.3	1626.3
Zhenpin A×Yanhui 559	99.7	105.9	11.0	198.0	151.0	25.7	2.4	1.8	8.6	23.8	1932.0
Zhenpin A×Huizi 04	102.3	112.3	14.4	223.8	185.2	28.4	2.4	1.9	8.3	27.7	1349.3
Zhenpin AHui 9368	104.7	103.2	12.8	225.1	201.8	24.0	2.9	2.0	9.1	26.2	1247.3
Zhenpin A×Kanghui98	97.7	111.8	10.3	211.6	189.0	24.5	2.6	2.0	9.9	27.2	1406.7
257A×Minghui 63	101.3	122.6	13.3	171.7	157.7	26.2	2.6	1.9	9.7	22.0	1473.0
257A×Zhenhui 084	96.0	114.8	14.0	247.2	210.3	28.4	2.1	1.8	9.3	23.3	1497.7
257A×Yanhui 559	99.3	118.1	23.2	270.7	245.6	28.6	2.8	1.9	8.9	32.7	1351.7
257A×Huizi 04	99.7	134.3	16.8	225.0	205.4	27.7	2.6	1.9	8.4	27.8	2053.2
257A×Hui 9368	97.0	105.9	13.5	243.2	204.3	26.9	2.2	1.8	9.1	25.0	1540.0
257A×Kanghui98	99.7	120.4	14.3	267.1	239.0	27.7	2.6	2.0	8.8	26.3	1911.0
II-32A×Minghui 63	109.3	114.7	13.8	174.4	145.5	28.5	2.6	1.9	8.5	28.0	745.7
II-32A×Zhenhui 084	109.0	101.7	13.5	208.8	174.7	23.8	2.7	1.8	8.4	21.7	804.9
II-32A×Yanhui 559	112.0	104.4	16.8	161.2	147.2	23.8	2.6	1.9	8.1	21.2	990.3
II-32A×Huizi 04	115.3	107.6	15.3	168.5	150.7	21.8	2.6	2.0	8.4	26.2	1109.7
II-32A×Hui 9368	110.3	99.5	23.6	165.6	156.8	27.1	2.5	1.9	8.5	22.5	978.1
II-32A×Kanghui98	108.0	98.8	16.4	219.4	197.7	28.4	2.4	1.9	8.4	22.7	935.7
Zhenshan 97 A ×Minghui 63	107.3	93.0	13.5	154.2	153.6	26.1	2.8	1.8	8.7	24.3	1931.3
Zhenshan 97 A ×Zhenhui 084	105.3	110.3	22.7	189.3	165.3	26.7	2.8	2.0	9.6	24.3	2017.3
Zhenshan 97 A ×Yanhui 559	103.3	109.5	9.4	224.9	202.2	30.3	2.6	2.0	8.3	26.7	707.3
Zhenshan 97 A×Huizi 04	107.3	113.2	12.6	219.1	174.5	28.3	3.2	2.1	7.9	27.5	1102.7
Zhenshan 97 A× Hui 9368	104.7	109.2	23.0	214.3	194.3	27.3	2.6	2.0	9.4	25.8	1317.0
Zhenshan 97 A ×Kanghui98	108.3	109.8	13.0	195.0	176.4	26.3	2.7	2.0	9.6	26.7	1531.0
Yuetai A×Minghui 63	108.0	115.2	19.9	224.6	195.3	25.4	2.5	1.9	9.3	26.8	679.0
Yuetai A×Zhenhui 084	104.7	101.1	11.1	156.8	120.8	21.4	2.3	1.9	8.6	22.7	1004.5
Yuetai A×Yanhui 559	91.7	112.6	23.3	220.1	123.2	24.2	2.5	2.0	10.0	23.2	1922.9
Yuetai A×Huizi 04	104.3	110.1	12.3	222.1	204.5	25.2	2.5	2.0	9.6	21.3	1464.7
Yuetai A×Hui 9368	102.0	107.8	10.5	209.1	181.6	25.8	2.4	1.8	8.3	26.2	1020.7
Yuetai A×Kanghui98	101.0	115.5	14.5	225.3	192.7	28.2	2.4	2.0	9.4	31.3	1212.7
You 1A ×Minghui 63	101.0	120.6	13.8	177.3	168.1	23.3	2.6	1.9	8.7	23.0	931.7
You 1A ×Zhenhui 084	100.0	117.2	18.8	226.2	207.7	26.0	2.7	1.9	9.6	26.5	1595.3
You 1A ×Yanhui 559	98.7	123.8	10.0	193.8	174.3	24.0	2.6	1.9	8.8	26.7	1443.9
You 1A ×Huizi 04	99.0	113.3	10.9	265.7	225.3	27.5	2.5	1.9	8.3	25.2	1756.7
You 1A ×Hui 9368	105.3	98.8	16.6	162.3	144.3	26.1	2.4	1.9	9.3	23.7	1174.7
You 1A ×Kanghui98	100.3	105.8	17.3	228.7	200.8	26.2	2.3	1.9	9.0	26.5	1428.0
Zhong 9A×Minghui 63	98.3	102.9	7.8	210.4	163.8	25.4	2.7	1.9	9.2	26.2	1616.7
Zhong 9A×Zhenhui 084	102.3	96.7	7.8	184.8	144.0	25.0	2.3	1.8	7.7	27.0	1225.0
Zhong 9A×Yanhui 559	103.3	111.0	12.0	141.2	123.3	21.3	2.4	1.8	8.5	25.0	868.7
Zhong 9A×Huizi 04	105.7	113.8	13.7	227.6	195.0	27.3	2.7	2.1	9.8	28.8	1020.0
Zhong 9A×Hui 9368	108.7	113.9	22.0	206.6	188.0	28.5	2.5	1.9	9.1	30.2	1315.3

Zhong 9A×Kanghui98	101.3	118.0	9.8	226.3	198.8	26.9	2.5	1.9	9.2	21.8	1309.3
**Japonica combinations**											
863A×C418	100.7	121.2	12.1	258.4	234.5	28.3	3.3	2.1	8.3	27.6	1223.0
863A×Ninghui8hao	102.3	108.7	13.1	151.4	139.0	24.7	3.1	2.2	8.0	25.8	1616.3
863A×Yunhui 4 hao	106.7	110.3	10.8	171.9	151.3	25.1	3.2	1.9	7.4	26.3	1126.0
863A×Zhehui 315	109.7	104.9	10.8	285.9	239.1	27.8	2.7	2.1	7.4	27.2	1396.7
863A×Yanhui R50	104.3	113.5	15.5	142.8	126.7	26.2	3.2	2.1	7.7	27.2	1234.3
863A×Xiushui 04R	104.7	109.3	13.3	146.2	124.6	21.0	3.1	1.8	6.8	25.0	1315.0
9201A×C418	106.7	108.9	11.5	174.6	152.5	25.0	3.1	2.1	7.9	27.8	1036.2
9201A×Ninghui8hao	109.0	108.4	14.7	223.8	204.8	24.8	3.1	2.2	7.5	25.8	1155.7
9201A×Yunhui 4 hao	108.7	103.5	8.1	262.3	226.4	27.3	3.1	2.1	7.7	26.5	1242.9
9201A×Zhehui 315	106.0	94.2	13.7	288.5	210.6	24.6	2.8	1.9	7.9	24.5	2005.9
9201A×Yanhui R50	108.7	98.5	12.5	172.4	158.8	28.4	3.4	2.2	7.7	27.8	1501.7
9201A×Xiushui 04R	103.7	99.8	20.8	134.1	125.2	24.5	3.2	2.1	7.9	27.2	1704.3
Xu 2A×C418	104.0	109.4	17.0	245.5	222.6	23.0	3.4	2.3	7.4	31.2	1580.3
Xu 2A×Ninghui8hao	104.3	106.9	11.0	141.9	121.1	23.1	3.2	2.2	7.2	27.2	842.4
Xu 2A×Yunhui 4 hao	96.7	84.9	11.5	113.0	100.1	19.6	3.2	2.0	7.4	25.0	661.3
Xu 2A×Zhehui 315	105.3	108.8	11.0	226.6	209.1	19.7	3.1	2.0	6.9	26.4	997.1
Xu 2A×Yanhui R50	103.0	93.7	10.6	155.9	137.0	19.8	2.9	2.2	7.9	25.0	1052.3
Xu 2A×Xiushui 04R	106.3	93.6	11.7	71.2	59.1	14.9	3.3	2.2	8.2	27.0	1850.5
Nanjing 46A×C418	106.0	114.2	10.0	278.7	259.0	25.1	3.2	2.1	7.7	31.8	1231.0
Nanjing 46A×Ninghui8hao	104.0	95.0	14.5	158.3	132.5	25.3	2.9	2.1	7.2	28.1	1315.3
Nanjing 46A×Yunhui 4 hao	103.7	108.0	20.0	182.1	147.7	26.3	2.9	2.0	7.4	21.2	849.6
Nanjing 46A×Zhehui 315	108.0	102.2	16.0	152.3	135.8	25.7	2.9	2.0	7.7	27.5	1404.3
Nanjing 46A×Yanhui R50	109.0	108.8	17.8	155.1	134.6	26.5	3.1	2.1	7.6	28.5	1435.0
Nanjing 46A×Xiushui 04R	105.3	102.6	13.1	197.9	171.7	19.6	3.4	2.2	7.6	27.5	1109.7
731A×C418	104.7	82.2	13.4	115.8	106.5	15.2	3.3	2.4	7.6	27.0	2492.4
731A×Ninghui8hao	104.7	101.8	14.6	135.9	124.9	25.8	3.2	2.2	7.9	27.0	1450.7
731A×Yunhui 4 hao	110.0	92.6	12.8	119.7	110.6	23.2	3.2	2.3	7.8	28.0	966.4
731A×Zhehui 315	96.0	93.2	15.8	246.1	231.8	26.8	2.7	2.0	8.7	27.5	786.4
731A×Yanhui R50	100.0	112.8	7.4	180.0	147.2	23.8	3.0	2.1	8.1	27.5	1620.0
731A×Xiushui 04R	102.7	101.0	11.8	173.8	156.9	24.1	3.1	2.1	7.6	26.8	929.3
Liuqianxin A×C418	102.3	106.9	18.7	147.3	109.5	25.0	3.1	1.9	7.5	22.8	1204.5
Liuqianxin A×Ninghui8hao	102.7	103.8	11.2	143.1	100.3	24.4	3.1	2.0	7.5	27.3	1653.1
Liuqianxin A×Yunhui 4 hao	105.3	104.1	9.3	244.8	215.3	25.6	3.2	2.2	7.8	25.3	1157.9
Liuqianxin A×Zhehui 315	104.7	96.3	11.8	156.5	138.4	22.1	3.3	2.1	8.0	24.8	1012.0
Liuqianxin A×Yanhui R50	104.3	106.8	13.5	239.5	205.8	27.3	3.1	2.2	7.7	27.0	1936.7
Liuqianxin A×Xiushui 04R	103.0	101.3	15.1	113.5	105.2	24.7	3.4	2.2	7.7	26.8	1073.9
6427A×C418	101.7	115.9	10.7	250.0	229.8	27.6	3.5	2.2	8.0	26.3	1569.9
6427A×Ninghui8hao	107.0	105.8	9.5	254.3	224.5	24.9	3.6	2.1	7.4	26.0	1927.3
6427A×Yunhui 4 hao	105.0	110.7	8.8	271.2	230.6	26.0	3.2	2.2	7.4	24.7	1636.7
6427A×Zhehui 315	104.3	100.6	6.8	250.8	204.0	23.5	3.0	2.1	7.4	27.2	1116.3
6427A×Yanhui R50	108.7	110.7	11.4	152.5	132.8	20.5	3.1	2.1	7.5	27.0	1709.7
6427A×Xiushui 04R	100.7	107.7	14.8	206.5	188.1	24.7	3.0	2.1	6.9	25.8	1209.3
Zhendao 88A×C418	106.7	101.4	17.0	223.9	202.3	25.7	3.3	2.2	7.9	27.5	1686.0
Zhendao 88A×Ninghui8hao	107.0	93.4	10.9	207.7	135.3	20.2	3.1	2.0	7.3	27.7	2024.0
Zhendao 88A×Yunhui 4 hao	106.0	101.3	15.2	166.9	124.1	21.4	3.1	2.2	7.3	26.0	682.9
Zhendao 88A×Zhehui 315	93.7	97.8	15.7	145.8	130.3	21.5	3.1	2.1	7.5	27.8	994.9
Zhendao 88A×Yanhui R50	107.7	106.9	16.3	155.9	112.4	20.2	3.1	2.1	7.8	26.2	1531.7
Zhendao 88A×Xiushui 04R	110.3	119.1	9.8	211.2	171.8	26.8	2.9	2.1	6.8	27.5	1110.7
Qingkong A×C418	89.3	107.8	16.2	218.0	154.7	25.4	2.9	2.0	7.1	27.5	1356.8
Qingkong A×Ninghui8hao	91.3	111.3	14.0	163.5	142.7	25.0	3.1	2.2	7.1	26.7	1229.3
Qingkong A×Yunhui 4 hao	104.0	111.7	19.3	192.7	150.3	25.7	3.0	1.9	6.6	25.0	2158.9

Qingkong A××Zhehui 315	101.0	125.5	19.5	224.1	155.8	25.4	3.1	2.0	7.0	27.3	1241.3
Qingkong A×Yanhui R50	96.3	134.5	13.3	175.4	119.9	25.6	3.2	2.1	7.8	27.2	1653.3
Qingkong A×Xiushui 04R	97.7	113.8	11.4	278.1	232.9	29.1	3.1	2.1	7.6	26.3	1451.0
Yueguang A×C418	93.0	120.5	13.5	225.2	196.1	28.6	2.6	1.9	9.1	21.7	1985.6
Yueguang A×Ninghui8hao	96.7	117.3	12.6	251.5	224.0	27.8	3.0	1.9	7.9	26.3	1402.9
Yueguang A×Yunhui 4 hao	99.3	103.2	8.8	191.3	124.3	24.0	3.1	2.1	7.3	23.8	966.9
Yueguang A×Zhehui 315	106.7	134.8	15.1	166.4	90.2	26.9	3.1	2.1	7.7	26.3	1194.9
Yueguang A×Yanhui R50	115.3	137.8	20.3	123.1	86.3	23.0	3.0	2.1	7.7	28.2	1550.0
Yueguang A×Xiushui 04R	100.3	129.3	17.8	251.6	207.5	25.6	3.2	2.0	7.1	27.5	1565.9
Wuqiang A×C418	104.3	94.4	14.4	215.8	184.7	20.5	3.0	1.9	7.6	28.3	1522.7
Wuqiang A×Ninghui8hao	110.7	89.1	9.8	149.6	128.7	20.0	3.1	2.1	7.9	27.7	1231.7
Wuqiang A×Yunhui 4 hao	114.7	91.3	10.6	242.8	153.9	21.6	3.1	2.0	8.0	28.5	866.3
Wuqiang A×Zhehui 315	113.7	98.1	13.5	158.3	147.1	19.7	3.1	2.3	7.4	30.2	1298.9
Wuqiang A×Yanhui R50	100.3	92.8	15.9	221.3	158.0	21.2	2.8	2.2	7.7	25.3	1133.3
Wuqiang A×Xiushui 04R	98.0	129.6	10.7	231.8	169.3	24.8	3.3	2.2	8.2	21.8	1086.9
Wuyujing 3A×C418	103.7	99.5	13.7	142.7	125.6	21.0	3.1	2.2	7.3	27.5	1666.7
Wuyujing 3A×Ninghui8hao	104.3	80.7	12.3	116.9	110.8	18.4	3.4	2.4	7.6	31.5	850.1
Wuyujing 3A×Yunhui 4 hao	110.7	109.8	9.0	247.1	225.5	23.7	3.2	2.2	8.2	27.7	1938.7
Wuyujing 3A×Zhehui 315	102.3	98.6	12.3	239.2	218.8	23.1	3.3	2.3	7.6	26.2	1942.1
Wuyujing 3A×Yanhui R50	102.7	96.0	11.9	150.6	130.5	21.6	3.2	2.2	8.0	28.0	1341.0
Wuyujing 3A×Xiushui 04R	106.7	109.6	12.8	257.3	240.7	23.9	3.2	2.1	7.6	26.0	1637.6
Liuyan 189A×C418	98.7	94.6	10.2	154.8	133.8	22.9	3.4	2.2	7.7	21.3	809.4
Liuyan 189A×Ninghui8hao	98.3	100.9	11.6	128.4	105.8	23.9	2.9	1.9	7.8	23.0	1173.5
Liuyan 189A×Yunhui 4 hao	95.3	91.3	12.7	134.1	123.8	22.7	3.2	2.2	7.9	28.3	872.8
Liuyan 189A×Zhehui 315	98.3	89.4	14.7	129.0	116.8	22.6	3.5	2.2	8.0	30.3	1241.9
Liuyan 189A×Yanhui R50	98.3	90.7	11.6	128.2	119.3	21.9	3.1	2.2	7.9	30.8	955.0
Liuyan 189A×Xiushui 04R	100.7	85.8	12.8	156.1	131.2	23.7	3.1	2.1	7.9	28.2	982.9

Days to heading (DH), plant height (PH), number of panicles per plant (NPPP), number of spikelets per panicles (NSPP), number of filled grains per panicles (NFGPP), panicle length (PL), seed width (SW), seed thickness (ST) ,seed length (SL), thousand grain weights (TGW) and grain yield per plot (GYPP).

Supporting Information Table S4-Number of identified SNPs on individual chromosomes of the indica and japonica cytoplasmic male sterile and restorer lines

**CMS lines**	Chr1	Chr2	Chr3	Chr4	Chr5	Chr6	Chr7	Chr8	Chr9	Chr10	Chr11	Chr12	Total
256A	1849	1609	1668	1290	1156	1341	1104	910	894	914	1124	947	**14,806**
Zhenpin A	2330	1718	1753	1506	1324	1605	1440	1206	1075	1093	1418	1095	**17,563**
257A	1948	1687	1737	1351	1161	1408	1230	1048	914	1047	1172	1005	**15,708**
II-32A	2081	1852	1844	1408	1238	1630	1404	1052	856	1121	1395	1022	**16,903**
Zhenshan 97A	2099	1849	1874	1476	1270	1594	1359	1033	849	1139	1410	1091	**17,043**
Yuetai A	1575	1245	1333	970	808	1029	893	696	672	745	1047	746	**11759**
You 1A	1709	1523	1411	1121	907	1228	1157	907	690	832	1095	902	**13,482**
Zhong 9A	2065	1629	1682	1298	1125	1380	1146	1027	776	988	1082	991	**15,189**
**Restorers**													
Minghui 63	1938	1381	1529	1133	910	1052	1107	774	832	862	1063	791	**13,372**
Zhenhui 084	2023	1550	1585	1183	1139	1262	1165	926	890	1013	1233	933	**14,902**
Yanhui 559	1753	1245	1333	960	864	979	794	816	813	774	1082	702	**12,115**
Huizi 04	379	370	161	219	70	302	170	169	147	433	338	353	**3,111**
Hui 9368	1757	1281	1274	992	847	917	970	765	596	749	1043	748	**11,939**
Kanghui98	1865	1420	1366	1023	946	1217	818	838	793	833	929	765	**12,813**

**CMS lines**	Chr1	Chr2	Chr3	Chr4	Chr5	Chr6	Chr7	Chr8	Chr9	Chr10	Chr11	Chr12	Total
863A	526	236	198	188	83	151	127	141	74	238	440	86	**2488**
9201A	268	242	147	206	122	209	256	182	60	143	462	343	**2640**
Xu 2A	423	249	169	244	72	220	199	185	161	145	523	513	**3103**
Nanjing 46A	261	253	180	185	199	206	192	200	45	145	335	328	**2529**
731A	391	388	172	211	72	228	150	174	169	168	483	207	**2813**
Liuqianxin A	247	249	174	276	121	230	174	141	120	151	603	284	**2770**
6427A	253	236	165	220	81	284	199	199	100	172	383	148	**2440**
Zhendao 88A	395	191	145	272	71	189	159	139	105	157	292	359	**2474**
Qingkong A	402	185	172	288	76	215	295	208	102	136	497	116	**2692**
Yueguang A	404	168	156	269	77	202	304	202	90	134	483	107	**2596**

Wuqiang A	1875	1525	1630	1251	1107	1287	1127	952	888	974	1103	932	**14651**
Wuyujing 3A	284	187	115	217	63	155	145	177	45	141	319	138	**1986**
Liuyan 189A	288	193	128	215	63	162	162	151	40	112	324	165	**2003**
**Restorer lines**													
C418	704	995	1101	443	388	597	753	487	285	635	958	135	**7481**
Ninghui8hao	1998	1527	1533	1176	1109	1218	1152	917	864	960	1192	885	**14531**
Yunhui 4 hao	471	399	124	281	128	276	395	427	48	325	1018	155	**4047**
Zhehui 315	1702	1385	1416	1157	880	1125	965	742	827	818	1044	734	**12795**
Yanhui R50	2061	1531	1583	1190	1123	1256	1168	934	864	1011	1166	949	**14836**
Xiushui 04R	243	257	129	208	230	299	118	129	44	209	482	146	**2494**

Supporting Information Table S5-Annotation of identified SNPs between indica and japonica cytoplasmic male sterile and restorer lines

CMS lines	Total	Intergenic	Splice site	Intron	3´-UTR	5´-UTR	Exon	Synonymous	Non-synonymous	
									Missense	Nonsense
256A	14,806	8,345	161	2,668	1,121	800	3,797	1543	1,967	27
Zhenpin A	17,563	10,164	168	3,061	1,253	856	4,423	1,720	2,352	28
257A	15,708	8,878	156	2,663	1,130	842	4,143	1,642	2,192	32
II-32A	16,903	9,636	175	2920	1,204	908	4,371	1,717	2,303	34
Zhenshan 97 A	17,043	9765	170	2,982	1,238	898	4,355	1,752	2,297	31
Yuetai A	11759	8956	154	2789	195	103	4012	1676	1939	32
You 1A	13,482	7,758	125	2,154	906	734	3,550	1428	1,886	30
Zhong 9A	15,189	8,489	157	2,588	1,105	823	4,139	1,669	2,185	35
**Restorers**										
Minghui 63	13,372	7,601	125	2075	886	682	3,764	1,472	2,021	25
Zhenhui 084	14,902	8574	146	2,384	1,017	765	4,010	1,575	2,170	30
Yanhui 559	12,115	6,766	110	1,849	814	694	3,550	1,443	1,829	29
Huizi 04	3,111	1721	22	353	198	220	1,003	333	609	11
Hui 9368	11,939	6,681	102	1,871	868	633	3,418	1,394	1,773	26
Kanghui98	12,813	7,140	119	2,093	914	684	3,625	1,472	1918	23

**CMS lines**	Total	Intergenic	Splice site	Intron	3´-UTR	5´-UTR	Exon	Synonymous	Non-synonymous	
									Missense	Nonsense
863A	2,488	1,349	18	264	184	165	878	262	528	12
9201A	2,640	1,528	18	245	144	171	863	263	535	11
Xu 2A	3,103	1,825	18	301	191	192	963	296	598	10
Nanjing 46A	2,529	1,452	14	237	152	168	835	242	526	10
731A	2,813	1,591	21	275	188	198	895	285	538	11
Liuqianxin A	2,770	1,554	9	242	150	187	950	316	569	11
6427A	2,440	1,389	16	270	167	151	752	238	460	6
Zhendao 88A	2,474	1,438	11	223	163	170	757	219	487	7
Qingkong A	2,692	1,533	18	294	149	191	827	265	508	6
Yueguang A	2,596	1,470	17	262	156	164	831	269	508	6
Wuqiang A	14,651	8,251	152	2,397	1,101	817	3958	1,580	2,105	31
Wuyujing 3A	1,986	1,042	7	140	121	127	815	246	506	10
Liuyan 189A	2,003	1,014	172	6	140	148	793	236	499	10
**Restorers**										
C418	7,481	4,153	63	1,106	499	412	2,328	877	1,268	16
Ninghui8hao	14,531	8,312	140	2,248	1,019	755	3,992	1,585	2,140	27
Yunhui 4 hao	4,047	2,319	26	529	274	238	1202	394	729	6
Zhehui 315	12,795	7329	122	1948	860	693	3,487	1,375	1894	24
Yanhui R50	14,836	8,446	155	2,378	1,032	780	4,006	1,614	2,149	30
Xiushui 04R	2,494	1,345	16	261	148	167	891	276	557	11

Supporting Information Table S6- Classifications of SNPs detected in indica and japonica cytoplasmic male sterile and restorer lines

**CMS lines**	**Transitions**		**Transversions**				
	C/T	G/A	C/G	A/T	A/C	G/T	Ts/Tv ratio
256A	2,266	2,143	675	770	843	774	1.36
Zhenpin A	2,730	2,615	843	899	951	969	1.37
257A	2,418	2,268	769	806	870	855	1.32
II-32A	2,627	2,516	794	864	887	909	1.40
Zhenshan 97 A	2,596	2,493	815	891	947	924	1.34
Yuetai A	365	364	129	129	151	154	1.32
You 1A	2,085	1,980	663	638	751	729	1.38
Zhong 9A	2,376	2,228	734	763	828	835	1.34
**Restorers**							
Minghui 63	2,037	1,926	696	671	740	735	1.34
Zhenhui 084	2,328	2,183	770	737	807	785	1.37
Yanhui 559	1,864	1,791	628	611	693	656	1.36
Huizi 04	419	392	243	159	190	227	0.90
Hui 9368	1,804	1,712	604	604	66	654	1.32
Kanghui98	1,955	1,889	639	604	719	707	1.36

CMS lines	Transitions		Transversions				
	C/T	G/A	C/G	A/T	A/C	G/T	Ts/Tv ratio
863A	318	315	192	120	131	179	0.90
9201A	381	313	203	114	152	187	0.92
2A	415	378	226	151	184	227	0.93
Nanjing 46A	342	285	209	128	151	186	0.82
731A	373	344	222	140	176	214	0.84
Liuqianxin A	382	329	214	148	152	198	0.90
6427A	333	282	191	124	141	181	0.85
Zhendao 88A	335	278	198	130	148	194	0.85
Qingkong A	359	345	204	137	172	194	0.92
Yueguang A	346	323	201	126	155	187	0.92
Wuqiang A	2,274	2,113	712	748	799	790	1.34
Wuyujing 3A	245	207	177	90	126	157	0.74
Liuyan 189A	244	206	181	104	139	158	0.73
**Restorers**							
C418	1,136	1,042	455	363	416	441	1.20
Ninghui8hao	2,239	2,131	740	718	788	771	1.35
Yunhui 4 hao	557	488	276	210	229	270	1.01
Zhehui 315	1,950	1,851	651	662	704	678	1.34
Yanhui R50	2,283	2,167	762	759	792	797	1.35
Xiushui 04R	345	279	192	115	115	182	0.82

Supporting Information Table S7- Parental genomes with favorable GCA alleles of 11 yield-related traits

**CMS lines**	DH	PH	NPPP	NSPP	NFGPP	PL	SW	ST	SL	TGW	GYPP
256A	4	6	1	-	-	-	-	-	-	3	
Zhenpin A	4	7	1	-	-	-	1		-	-	1
257A	2	7	1	-	-	-	1	-	-	3	1
II-32A	-	7	1	-	-	1	1	-	5	4	2
Zhenshan 97A	2	7	1	-	-	1	-	-	-	1	1
Yuetai A	1	6	1	-	-	-	1	-	-	-	-
You 1A	1	7	1	-	-	-	1	-	1	3	-
Zhong 9A	3	6	1	-	-	-	1	-	-	2	-
863A	4	3	1	-	1	2	-	2	1	-	-
9201A	4	6	2	-	1	1	-	1	1	-	-
Xu 2A	5	6	1	-	-	2	1	1	5	-	-
Nanjing 46A	4	6	2	-	1	1	-	1	1	-	-
731A	5	7	1	-	-	-	1	1	-	-	-
Liuqianxin A	4	4	1	-	2	-	1	2	1	1	-
6427A	5	4	1	1	-	-	1	2	2	-	-
Zhendao 88A	5	1	1	-	-	1	-	1	5	-	-
Qingkong A	5	-	3	1	-	2	-	3	2	1	-
Yueguang A	5	-	3	1	-	4	-	3	1	1	-
Wuqiang A	4	7	1	-	-	-	1	-	-	1	-
Wuyujing 3A	5	7	1	-	-	1	1	3	-	-	-
Liuyan 189A	5	7	1	-	-	-	1	3	-	-	-
**Restorers**											-
Minghui 63	3	7	2	-	-	-	-	-	-	-	-
Zhenhui 084	2	7	1	-	-	-	1	-	-	1	1
Yanhui 559	1	6	2	1	-	1	-	-	2	-	1
Huizi 04	3	6	1	-	-	-	1	1	4	-	-
Hui 9368	2	7	2	-	-	-	-	1	3	1	-
Kanghui98	1	6	1	-	-	-	-	1	-	-	-
C418	5	5	2	-	-	1	-	-	-	1	1
Ninghui8hao	3	7	1	-	-	-	1	-	-	1	1
Yunhi 4 hao	4	5	2	-	-	5	1	-	-	1	-
Zhehui 315	2	5	3	-	-	1	-	-	3	-	-
Yanhui R50	3	5	1	-	-	1	1	-	-	1	1

Xiushui 04R	4	4	1	-	-	1	1	3	-	-	-

Character (–) shows that none of the superior alleles of GCA are situated within the sequenced genome of parents of hybrid rice.

alleles of DH and PH are positive in sense of favoring shorter plant height and early maturity, Days to heading (DH), plant height (PH), number of panicles per plant (NPPP), number of spikelets per panicles (NSPP), number of filled grains per panicles (NFGPP), panicle length (PL), seed width (SW), seed thickness (ST), seed length (SL), thousand grain weights (TGW) and grain yield per plot (GYPP).

Supporting Information Table S8- Annotations of detected genes within the intervals of associated SNPLDBs for GCA of 11 yield related traits

Traits	Associated SNPLBDs	Chr	Gene id (MSU. v7)	Gene product (protein) information	Nucleotide length	Biological process	Molecular process	Ref
DH	1_BLOCK_28934801_29133392	1	LOC_Os01g50400.1	STE_MEKK_ste11_MAP3K.5 - STE kinases include homologs to sterile 7, sterile 11 and sterile 20 from yeast, expressed	1257	metabolic and cellular process	kinase activity and protein binding	
			LOC_Os01g50420.1	STE_MEKK_ste11_MAP3K.7 - STE kinases include homologs to sterile 7, sterile 11 and sterile 20 from yeast, expressed	1626	protein modification process	kinase activity	
			LOC_Os01g50460.1	nodulin MtN3 family protein, putative, expressed	693	transport and cellular process	transporter activity	
			LOC_Os01g50470.1	regulator of chromosome condensation, putative, expressed	3135		binding	
			LOC_Os01g50490.1	cytochrome P450, putative, expressed	1521	metabolic process	oxygen binding	
			LOC_Os01g50530.1	cytochrome P450, putative, expressed	1236	lipid metabolic process, multicellular organismal development	catalytic activity	
			LOC_Os01g50590.1	cytochrome P450, putative, expressed	1551	anatomical structure morphogenesis	oxygen binding	
			LOC_Os01g50616.1	phosphatidylinositol transfer, putative, expressed	2016	transport	transporter activity	
			LOC_Os01g50622.1	DNA-binding protein DSP1, putative, expressed	996	response to stress	nuclease activity	
			LOC_Os01g50680.1	OsSub2 - Putative Subtilisin homologue, expressed	1281	protein metabolic process	hydrolase activity	(Bessho-Uehara

								et al., 2016)
			LOC_Os01g50690.1	WD domain, G-beta repeat domain containing protein, expressed	2706	biological_process	nucleotide binding	
	2_BLOCK_23246549_23402926	2	LOC_Os02g38690.1	protein phosphatase 2C containing protein, expressed	2292	protein modification process	plasma membrane	
			LOC_Os02g38680.1	EMB2423, putative, expressed	2112	reproduction and embryo development	molecular_function	
			LOC_Os02g38580.1	protein phosphatase 2C, putative, expressed	1566	protein modification process	hydrolase activity	
			LOC_Os02g38494.1	protein phosphatase 2C, putative, expressed	1566	protein modification process	hydrolase activity	
			LOC_Os02g38470.1	B3 DNA binding domain containing protein, expressed	3147	biological_process	molecular_function	
	4_BLOCK_11861449_12047086	4	LOC_Os04g21320.1	membrane associated DUF588 domain containing protein, putative, expressed	630	response to abiotic stimulus	protein binding	
			LOC_Os04g21160.1	triacylglycerol lipase 1 precursor, putative, expressed	1407	metabolic process	catalytic activity	
			LOC_Os04g21110.1	phosphoribulokinase/Uridine kinase family protein, expressed	2661	cellular process	hydrolase activity	
	4_BLOCK_14047340_14210685	4	LOC_Os04g24710.1	ubiquitin carboxyl-terminal hydrolase, family 1, putative, expressed	1509	post-embryonic development	embryo development	
			LOC_Os04g24600.1	cysteine proteinase 1 precursor, putative, expressed	1146	response to stress	hydrolase activity	
			LOC_Os04g24550.1	ATP-binding region, ATPase-like domain containing protein, expressed	1872		nucleotide binding	
			LOC_Os04g24520.1	ribosomal protein L51, putative, expressed	213	metabolic process	catalytic activity	
			LOC_Os04g24510.1	OsWAK36 - OsWAK receptor-like protein kinase, expressed	2052	metabolic process	kinase activity	

	5_BLOCK_6791185_6982283	5	LOC_Os05g11950.1	GDSL-like lipase/acylhydrolase, putative, expressed	1113	metabolic process	hydrolase activity	
			LOC_Os05g11970.1	GDSL-like lipase/acylhydrolase, putative, expressed	1143	lipid metabolic process	hydrolase activity	
			LOC_Os05g11980.1	timeless protein, expressed	3624	biological_process	nucleus	
			LOC_Os05g11990.1	TTL1, putative, expressed	1446	response to endogenous stimulus	binding	
			LOC_Os05g12040.1	cytochrome P450 51, putative, expressed	1503	biosynthetic process	oxygen binding	(Xia et al., 2015)
			LOC_Os05g12090.1	VQ domain containing protein, putative	285	response to stress	molecular_function	
			LOC_Os05g12130.1	EMB2261, putative, expressed	2469	post-embryonic development		
			LOC_Os05g12140.1	Leucine Rich Repeat family protein, expressed	1821			
			LOC_Os05g12150.1	endoglucanase precursor, putative, expressed	1890	carbohydrate metabolic process	catalytic activity	
			LOC_Os05g12170.1	plant-specific domain TIGR01589 family protein, putative, expressed	1053	biological_process	molecular_function	
			LOC_Os05g12180.1	chalcone synthase, putative, expressed	1179	response to endogenous stimulus	transferase activity	
	5_BLOCK_27886769_28020620	5	LOC_Os05g48660.1	MRH1, putative, expressed	1365	anatomical structure morphogenesis	nucleotide binding	
			LOC_Os05g48700.1	gibberellin 2-beta-dioxygenase, putative, expressed	1062	response to abiotic stimulus	catalytic activity	(Lo et al., 2008)
			LOC_Os05g48690.1	transcription factor like protein, putative, expressed	1041	nucleobase, nucleoside, nucleotide and nucleic acid metabolic process	sequence-specific DNA binding transcription factor activity	

			LOC_Os05g48670.1	OsFBT10 - F-box and tubby domain containing protein, expressed	1197	metabolic process	sequence-specific DNA binding transcription factor activity	
			LOC_Os05g48750.1	3-deoxy-manno-octulosonate cytidylyltransferase, putative, expressed	894	biosynthetic process	transferase activity	
			LOC_Os05g48800.2	drought induced 19 protein, putative, expressed	681	response to stress	response to abiotic stimulus	(Wang et al., 2014b)
			LOC_Os05g48810.1	dnaJ domain containing protein, expressed	1089	protein metabolic process	protein binding	
			LOC_Os05g48820.1	DDT, putative, expressed	5568	nucleobase, nucleoside, nucleotide and nucleic acid metabolic process	sequence-specific DNA binding transcription factor activity	
			LOC_Os05g48855.1	GTP binding protein, putative, expressed	729	nucleobase, nucleoside, nucleotide and nucleic acid metabolic process	hydrolase activity	
			LOC_Os05g48850.1	no apical meristem protein, putative, expressed	945	nucleobase, nucleoside, nucleotide and nucleic acid metabolic process	sequence-specific DNA binding transcription factor activity	
			LOC_Os07g46480.1	eukaryotic aspartyl protease domain containing protein, expressed	1350	protein metabolic process	hydrolase activity	
			LOC_Os07g46490.1	phosphatidylinositol-4-phosphate 5-kinase, putative, expressed	2376	cellular component organization	kinase activity	
			LOC_Os07g46500.1	ankyrin repeat domain containing protein, putative, expressed	453	biological_process		

			LOC_Os07g46520.1	rhythmically expressed gene 2 protein, putative, expressed	849	metabolic process	catalytic activity	
			LOC_Os07g46540.1	condensin complex subunit 1, putative, expressed	3978	cellular component organization	cell cycle	
			LOC_Os07g46550.1	annexin, putative, expressed	915	response to abiotic stimulus	lipid binding	
			LOC_Os07g46555.1	F-box domain containing protein, expressed	897	biological_process	molecular_function	
			LOC_Os07g46560.1	seven in absentia protein family domain containing protein, expressed	909	protein metabolic process	catalytic activity	
			LOC_Os07g46570.1	glutaredoxin, putative, expressed	636	metabolic process	catalytic activity	
			LOC_Os07g46590.1	SNF2 family N-terminal domain containing protein, expressed	3009			(Hu et al., 2012; Hu et al., 2013)
	11_BLOCK_19263139_19263152	11	LOC_Os11g40030.2	cyclin-dependent kinase inhibitor, putative, expressed	678			
	11_BLOCK_23861754_23986595	11	LOC_Os11g40180.1	dirigent, putative, expressed	891	response to stress	molecular_function	
			LOC_Os11g40150.1	DNA repair protein Rad51, putative, expressed	1020	DNA metabolic process	protein binding	(Morozumi et al., 2013)
			LOC_Os11g40140.1	peptidase, T1 family, putative, expressed	714	metabolic process	nuclease activity	
			LOC_Os11g40180.1	dirigent, putative, expressed	891	response to stress	molecular_function	
			LOC_Os11g40110.1	RWP-RK, putative, expressed	750	biosynthetic process	sequence-specific DNA binding transcription factor activity	
			LOC_Os11g40100.3	GRF-interacting factor 2, putative, expressed	618	biological_process	protein binding	(Liu et al., 2014)
			LOC_Os11g40080.1	lipin, N-terminal conserved region family protein, expressed	4164	response to extracellular stimulus	hydrolase activity	

			LOC_Os11g40090.2	A49-like RNA polymerase I associated factor family protein, expressed	1488	biosynthetic process	transferase activity	(Li et al., 2015)
PH	2_BLOCK_35817740_35924060	2	LOC_Os02g58790.1	cell division inhibitor, putative, expressed	1080	cellular component organization	binding	
			LOC_Os02g58720.2	peroxidase precursor, putative, expressed	981	response to stress	catalytic activity	
			LOC_Os02g58730.1	ras-related protein, putative, expressed	594	catabolic process	nucleotide binding	
			LOC_Os02g58640.1	tumor susceptibility gene 101, putative, expressed	1209	cellular process	protein binding	
			LOC_Os02g58670.1	bZIP transcription factor domain containing protein, expressed	429	flower development	protein binding	(Ji et al., 2009; Nijhawan et al., 2008)
			LOC_Os02g58730.1	ras-related protein, putative, expressed	594	catabolic process	nucleotide binding	(Xu et al., 2004)
			LOC_Os02g58660.1	ATCHX15, putative, expressed	2493	transport	transporter activity	
	3_BLOCK_9933834_10133555	3	LOC_Os03g18130.1	asparagine synthetase, putative, expressed	1815	biosynthetic process	catalytic activity	
			LOC_Os03g18120.1	proteins of unknown function domain containing protein, expressed	1329	biosynthetic process		
			LOC_Os03g18110.1	proteins of unknown function domain containing protein, putative, expressed	1182	response to biotic stimulus	molecular_function	
			LOC_Os03g18080.1	SacI homology domain containing protein, expressed	1803	metabolic process		
			LOC_Os03g18070.1	omega-3 fatty acid desaturase, chloroplast precursor, putative, expressed	1377	biosynthetic process	catalytic activity	(Liu et al., 2012; Nair et al., 2009; Yara et al., 2007)
			LOC_Os03g18030.1	leucoanthocyanidin dioxygenase, putative, expressed	1164	metabolic process	catalytic activity	
			LOC_Os03g18050.1	OsSAUR13 - Auxin-responsive SAUR gene family member, expressed	624	response to endogenous stimulus	molecular_function	

			LOC_Os03g18010.1	phospholipase C, putative, expressed	1455	signal transduction	hydrolase activity	
			LOC_Os03g17990.1	Yip1 domain containing protein, expressed	771	biological_process	molecular_function	
			LOC_Os03g17980.1	CAMK_KIN1/SNF1/Nim1_like_AMPKh.2 - CAMK includes calcium/calmodulin depedent protein kinases, expressed	1647	reproduction	kinase activity	
			LOC_Os03g17940.1	phosphatase, putative, expressed	675	biological_process	catalytic activity	
			LOC_Os03g17930.1	alpha-taxilin, putative, expressed	1311	biological_process		
	S4_16441661	4						
	6_BLOCK_738449_922752	6	LOC_Os06g02340.1	OsFBX183 - F-box domain containing protein, expressed	1230	biological_process	molecular_function	
			LOC_Os06g02380.2	T-complex protein, putative, expressed	1806	protein metabolic process	protein binding	
			LOC_Os06g02400.1	OsFBO18 - F-box and other domain containing protein, expressed	2775	nucleobase, nucleoside, nucleotide and nucleic acid metabolic process	molecular_function	
			LOC_Os06g02490.1	acyl CoA binding protein, putative, expressed	276	response to stress	lipid binding	(Meng et al., 2014)
			LOC_Os06g02500.1	superoxide dismutase, chloroplast, putative, expressed	1176	response to abiotic stimulus	catalytic activity	
			LOC_Os06g02510.2	ribosomal protein L13, putative, expressed	627	cellular process	structural molecule activity	
			LOC_Os06g02520.1	interacting protein of DMI3, putative, expressed	1527			
			LOC_Os06g02550.1	CPuORF21 - conserved peptide uORF-containing transcript, expressed	1455	metabolic process	nucleotide binding	
			LOC_Os06g02560.1	growth-regulating factor, putative, expressed	1071	multicellular organismal development		(Choi et al., 2004)
			LOC_Os06g02570.1	syntaxin, putative, expressed	993	cellular component organization	protein binding	

			LOC_Os06g02590.1	DUF1336 domain containing protein, expressed	1962	biological_process	lipid binding	
	6_BLOCK_970592_1159740	6	LOC_Os06g02780.1	aspartic protease, putative, expressed	1485	protein metabolic process	hydrolase activity	
			LOC_Os06g02900.1	aspartic proteinase nepenthesin-2 precursor, putative, expressed	1488	protein metabolic process	hydrolase activity	
			LOC_Os06g03080.1	aspartic protease, putative, expressed	1329	protein metabolic process	hydrolase activity	
	6_BLOCK_2655251_2849385	6	LOC_Os06g05860.1	6-phosphofructokinase, putative, expressed	1677	carbohydrate metabolic process	protein binding	
			LOC_Os06g05880.1	profilin domain containing protein, expressed	396	cellular component organization	protein binding	
			LOC_Os06g05890.1	B-box zinc finger family protein, putative, expressed	1083	biosynthetic process	sequence-specific DNA binding transcription factor activity	
			LOC_Os06g05900.1	methyltransferase, putative, expressed	984	post-embryonic development	transferase activity	
			LOC_Os06g05910.2	methyltransferase domain containing protein, expressed	711	biosynthetic process	transferase activity	
			LOC_Os06g05920.1	Rf1, mitochondrial precursor, putative, expressed	2154	post-embryonic development		
			LOC_Os06g05940.1	DTA2, putative, expressed	1644			
			LOC_Os06g05980.1	transporter family protein, putative, expressed	1296			
			,LOC_Os06g05990.1	zinc finger family protein, putative, expressed	1524	protein modification process	catalytic activity	
			LOC_Os06g06030.1	peptidase, T1 family, putative, expressed	834	protein metabolic process	hydrolase activity	
			LOC_Os06g06050.1	OsFBL27 - F-box domain and LRR containing protein, expressed	2163	multicellular organismal development	catalytic activity	(Ishikawa et al., 2005; Sang et al.,

								2014; Yan et al., 2007; Yasuno et al., 2007; Zhao et al., 2014)
			LOC_Os06g06080.1	serine esterase family protein, putative, expressed	840			
	S7_10979899	7	LOC_Os07g18560.1	OsFBLD6 - F-box, LRR and FBD domain containing protein, expressed	1428			
	7_BLOCK_24627656_24806954	7	LOC_Os07g41180.1	RNA-binding protein-like, putative, expressed	1710	transport	nucleotide binding	
			LOC_Os07g41190.1	WD domain, G-beta repeat domain containing protein, expressed	1674	biological_process	nucleotide binding	
			LOC_Os07g41220.1	peptidase aspartic family protein, putative, expressed	738	protein metabolic process	hydrolase activity	
			LOC_Os07g41230.1	esterase, putative, expressed	1155	metabolic process	hydrolase activity	
			LOC_Os07g41250.1	peptide transporter PTR2, putative, expressed	2037	transport	transporter activity	.
			LOC_Os07g41280.3	6-phosphogluconolactonase, putative, expressed	843	carbohydrate metabolic process	catalytic activity	
			LOC_Os07g41310.1	COBRA, putative, expressed	1341			
			LOC_Os07g41330.1	mitochondrial import inner membrane translocase subunit Tim17, putative, expressed	672	transport	transporter activity	
			LOC_Os07g41320.1	COBRA-like protein precursor, putative, expressed	1422	cellular process		
			LOC_Os07g41370.1	OsMADS18 - MADS-box family gene with MIKCc type-box, expressed	750	cell differentiation	protein binding	(Kobayashi et al., 2012; Lee et al., 2004; Masiero et al., 2002)

			LOC_Os07g41360.1	alpha-1,4-glucan-protein synthase, putative, expressed	1101	carbohydrate metabolic process	catalytic activity	
	7_BLOCK_26549570_26746263	7	LOC_Os07g44499.1	peroxidase, putative, expressed	1155	response to stress	catalytic activity	
			LOC_Os07g44560.1	AMP-binding domain containing protein, expressed	1830	response to stress	catalytic activity	
			LOC_Os07g44610.1	pyrrolidone-carboxylate peptidase, putative, expressed	660	protein metabolic process		
			LOC_Os07g44640.1	ZOS7-13 - C2H2 zinc finger protein, expressed		protein modification process	binding	
			LOC_Os07g44660.1					
	8_BLOCK_6251405_6277158	8	LOC_Os08g10630.1	metal cation transporter, putative, expressed	1191	biological_process	transporter activity	(Chen et al., 2008)
	9_BLOCK_12636807_12697367	9	LOC_Os09g20990.1	trehalose-6-phosphate synthase, putative, expressed	2592	biosynthetic process	transferase activity	
			LOC_Os09g21000.1	potassium transporter, putative, expressed	2634	transport	transporter activity	
	S10_2115860	10						
	10_BLOCK_5442582_5562381	10	LOC_Os10g10040.1	cytochrome P450, putative, expressed	1185	metabolic process	oxygen binding	
			LOC_Os10g10080.1	exostosin family domain containing protein, expressed	1254	carbohydrate metabolic process	catalytic activity	
			LOC_Os10g10130.1	OsWAK112d - OsWAK receptor-like protein kinase, expressed	3048	protein modification process	kinase activity	(Delteil et al., 2016)
	10_BLOCK_10531770_10728242	10	LOC_Os10g20990.1	ribosomal protein S12 containing protein, expressed	1686	translation	structural molecule activity	
			LOC_Os10g21000.1	nucleoside-triphosphatase, putative, expressed	2109	metabolic process	hydrolase activity	
			LOC_Os10g21090.1	ATP binding protein, putative, expressed	1995	protein modification process	nucleotide binding	
			LOC_Os10g21100.1	glycosyl hydrolase family 10 protein, putative, expressed	1761	carbohydrate metabolic process	catalytic activity	
NPPP	2_BLOCK_22143883_22260164	2	LOC_Os02g47440.1	syntaxin, putative, expressed	804	response to abiotic stimulus	protein binding	

			LOC_Os02g47470.1	cytochrome P450, putative, expressed	1416	response to stress	catalytic activity	(Saika et al., 2007; Zhu et al., 2009)
			LOC_Os02g47500.1	nodulin, putative, expressed	1191			
			LOC_Os02g47510.1	9-cis-epoxycarotenoid dioxygenase 1, chloroplast precursor, putative, expressed	1917		protein binding	(Zhu et al., 2009)
			LOC_Os02g47560.1	DNA-binding protein, putative, expressed	1254	metabolic process	biological_process	
			LOC_Os02g47570.1	phosphatase, putative, expressed	828	metabolic process	catalytic activity	
			LOC_Os02g47590.1	ornithine carbamoyltransferase, putative, expressed	1104	biological_process	molecular_function	
			LOC_Os02g47600.2	soluble inorganic pyrophosphatase, putative, expressed	645	metabolic process	hydrolase activity	
			LOC_Os02g47610.1	nmrA-like family domain containing protein, expressed	921	metabolic process	catalytic activity	
			LOC_Os02g47620.1	hydrolase, alpha/beta fold family domain containing protein, expressed	1284	metabolic process	catalytic activity	
			LOC_Os02g47650.1	universal stress protein domain containing protein, putative, expressed	513	response to stress	protein binding	
	S6_7843151	6						
	9_BLOCK_17535393_17676486	9	LOC_Os09g28880.1	DUF617 domain containing protein, expressed	735			
			LOC_Os09g28930.1	DNA binding protein, putative, expressed	1005			
			LOC_Os09g28940.1	ubiquitin carboxyl-terminal hydrolase domain containing protein, expressed	2814	protein metabolic process	hydrolase activity	
			LOC_Os09g28900.1	basic helix-loop-helix, putative, expressed	648	biosynthetic process	DNA binding	
			LOC_Os09g28910.1	carbonic anhydrase, chloroplast precursor, putative, expressed	1002	biological_process	catalytic activity	
	9_BLOCK_19575308_19760644	9	LOC_Os09g33460.1	double-stranded RNA binding motif containing protein, expressed	1215	biological_process	RNA binding	
			LOC_Os09g32952.1	L-ascorbate oxidase precursor, putative, expressed	1725	metabolic process	binding	
			LOC_Os09g32860.1	OsFBX335 - F-box domain containing protein, expressed	1236	biological_process	molecular_function	

			LOC_Os09g32870.1	OsFBX336 - F-box domain containing protein, expressed	915			
			LOC_Os09g32910.1	sFBX337 - F-box domain containing protein, expressed	1389			
			LOC_Os09g32944.1	OsSPL18 - SBP-box gene family member, expressed	1419	flower development	sequence-specific DNA binding transcription factor activity	
			LOC_Os09g32948.1	OsMADS8 - MADS-box family gene with MIKCc type-box, expressed	747	flower development	protein binding	(Arora et al., 2007; Cui et al., 2010)
			LOC_Os09g32964.1	peroxidase precursor, putative, expressed	996	response to stress	catalytic activity	
			LOC_Os09g32968.2	emp24/gp25L/p24 family protein, putative, expressed	636	transport	transporter activity	
			LOC_Os09g32976.1	ribosomal protein L7Ae, putative, expressed	777	translation	structural molecule activity	
			LOC_Os09g32988.1	POEI18 - Pollen Ole e I allergen and extensin family protein precursor, expressed	789			
			LOC_Os09g33450.1	zinc finger DHHC domain-containing protein, putative, expressed	1326		binding	
	11_BLOCK_18431697_18435610	11	LOC_Os11g31540.1	BRASSINOSTEROID INSENSITIVE 1-associated receptor kinase 1 precursor, putative, expressed	753	metabolic process	protein binding	
	11_BLOCK_18901416_19072571	11	LOC_Os11g32260.1	lysosomal alpha-mannosidase precursor, putative, expressed	3063	metabolic process	binding	
			LOC_Os11g32100.1	inducer of CBF expression 1, putative, expressed	1575	post-embryonic development	sequence-specific DNA binding transcription factor activity	(Arora et al., 2007)
			LOC_Os11g32110.1	auxin response factor, putative, expressed	2562	biosynthetic process	sequence-specific DNA binding	

							transcription factor activity	
			LOC_Os11g32170.1	stripe rust resistance protein Yr10, putative, expressed	4155	response to stress	nucleotide binding	
			LOC_Os11g32240.1	cytochrome P450 51, putative, expressed	1473	biosynthetic process	oxygen binding	
			LOC_Os11g32210.1	jacalin-like lectin domain containing protein, expressed	4161	response to stress	nucleotide binding	
	11_BLOCK_23861754_23986595	11	LOC_Os11g40150.1	DNA repair protein Rad51, putative, expressed	1020	metabolic process	protein binding	(Morozumi et al., 2013)
			LOC_Os11g40080.1	lipin, N-terminal conserved region family protein, expressed	4164	response to extracellular stimulus	response to extracellular stimulus	
			LOC_Os11g40090.2	A49-like RNA polymerase I associated factor family protein, expressed	1488	biosynthetic process	DNA binding	(Li et al., 2015)
			LOC_Os11g40100.3	GRF-interacting factor 2, putative, expressed	618	GRF-interacting factor 2, putative, expressed	protein binding	(Liu et al., 2014)
			LOC_Os11g40180.1	dirigent, putative, expressed	891	response to stress	molecular_function	
			LOC_Os11g40140.1	peptidase, T1 family, putative, expressed	714	metabolic process	nuclease activity	
	11_BLOCK_17820001_17945021	11						
	11_BLOCK_19309311_19309329	11						
NSPP	7_BLOCK_9972678_9972706	7	LOC_Os07g16950.4	Mak16 protein domain containing protein, expressed	873			
	S7_26929982	7						
NFGPP	2_BLOCK_24571861_24661819	2	LOC_Os02g40530.1	MYB family transcription factor, putative, expressed	870	biosynthetic process	DNA binding	(Schmidt et al., 2013)
			LOC_Os02g40664.1	zinc finger family protein, putative, expressed	2130	protein modification process	catalytic activity	
			LOC_Os02g40550.1	Lung seven transmembrane receptor domain containing protein, putative, expressed	1395			

	2_BLOCK_25142102_25304942	2	LOC_Os02g41930.1	OsFBX57 - F-box domain containing protein, expressed	1197			
			LOC_Os02g41860.1	aquaporin protein, putative, expressed	870	cellular process	transporter activity	(Sakurai et al., 2005)
			LOC_Os02g41890.1	phytosulfokine receptor precursor, putative, expressed	3159	protein modification process	kinase activity	
			LOC_Os02g41954.1	gibberellin 2-beta-dioxygenase 7, putative, expressed	1080	cellular process	catalytic activity	(Lo et al., 2008)
			LOC_Os02g42040.1	MIF4G domain containing protein, putative, expressed	3315	translation	binding	
	2_BLOCK_25359391_25409193	2	LOC_Os02g42210.1	membrane-associated salt-inducible protein like, putative, expressed	1368			
			LOC_Os02g42170.1	phospholipase, putative, expressed	975	response to stress	hydrolase activity	
			LOC_Os02g42190.1	OsWAK16 - OsWAK receptor-like protein kinase, expressed	2514	cell growth	binding	
			LOC_Os02g42200.1	transcription factor-related, putative, expressed	5463			
			LOC_Os02g42230.1	Putative single-stranded DNA binding complex subunit 2, expressed	897	metabolic process	protein binding	
	2_BLOCK_26014682_26204848	2	LOC_Os02g43330.1	homeobox associated leucine zipper, putative, expressed	786	response to endogenous stimulus	sequence-specific DNA binding transcription factor activity	
			LOC_Os02g43180.1	OsGrx_C3 - glutaredoxin subgroup I, expressed	396	metabolic process	catalytic activity	(Garg et al., 2010)
			LOC_Os02g43194.1	aldehyde dehydrogenase, putative, expressed	1464	metabolic process	protein binding	
			LOC_Os02g43280.1	aldehyde dehydrogenase, putative, expressed	1476	metabolic process	protein binding	
			LOC_Os02g43350.1	OsFtsH7 FtsH protease, homologue of AtFtsH7, expressed	2469	protein metabolic process	hydrolase activity	
			LOC_Os02g43340.2	hydrolase, alpha/beta fold family domain containing protein, expressed	1341	metabolic process	hydrolase activity	

			LOC_Os02g43360.1	cytochrome b5-like Heme/Steroid binding domain containing protein, expressed	417		binding	
	4_BLOCK_19576610_19769633	4	LOC_Os04g32670.1	exostosin family domain containing protein, expressed	1263	metabolic process	catalytic activity	
			LOC_Os04g32620.1	ethylene-responsive transcription factor ERF114, putative, expressed	807	response to stress	sequence-specific DNA binding transcription factor activity	(Wamaitha et al., 2012)
			LOC_Os04g32540.1	OsSCP24 - Putative Serine Carboxypeptidase homologue, expressed	1413	protein metabolic process	hydrolase activity	
			LOC_Os04g32550.1	defender against cell death 1, putative, expressed	345	cell death	molecular_function	
			LOC_Os04g32560.1	ATP-dependent Clp protease ATP-binding subunit clpA homolog CD4B,chloroplast precursor, putative, expressed	2757	cellular component organization	hydrolase activity	(Singh et al., 2010)
			LOC_Os04g32620.1	ethylene-responsive transcription factor ERF114, putative, expressed	807	response to stress	sequence-specific DNA binding transcription factor activity	(Wamaitha et al., 2012)
			LOC_Os04g32590.1	transcription factor, putative, expressed	990	biosynthetic process	sequence-specific DNA binding transcription factor activity	
			LOC_Os04g32740.1	hydrolase, NUDIX family, domain containing protein, expressed	669	metabolic process	hydrolase activity	
			LOC_Os04g32650.1	tRNA synthetase class II core domain containing protein, expressed	2022	nucleobase, nucleoside, nucleotide and nucleic acid metabolic	catalytic activity	
			LOC_Os04g32660.1	cytochrome b-c1 complex subunit Rieske, mitochondrial precursor, putative, expressed	837	metabolic process	binding	

	7_BLOCK_10991803_11175112	7	LOC_Os07g18720.1	tetratricopeptide repeat containing protein, putative, expressed	1452	biological_process	binding	
	9_BLOCK_11128244_11230987	9	LOC_Os09g18260.1	senescence-induced receptor-like serine/threonine-protein kinase precursor, putative, expressed	2559	response to stress	kinase activity	
			LOC_Os09g18159.1	light repressible receptor protein kinase, putative, expressed	2028	metabolic process	kinase activity	
	11_BLOCK_18121821_18288410	11	LOC_Os11g31190.1	nodulin MtN3 family protein, putative, expressed	912	transport	transporter activity	(Antony et al., 2010)
	11_BLOCK_22899060_23033776	11	LOC_Os11g38650.1	UDP-glucoronosyl/UDP-glucosyl transferase, putative, expressed	1473	transferase activity		
			LOC_Os11g38670.1	DEAD-box ATP-dependent RNA helicase, putative, expressed	1872			
	12_BLOCK_18717643_18913133	12	LOC_Os12g31350.1	SSXT protein, putative, expressed	558	biological_process	protein binding	
			LOC_Os12g31160.1	MLA10, putative, expressed	2808	response to stress	nucleotide binding	
			LOC_Os12g31380.1	initiation factor 2 subunit family domain containing protein, expressed	1143	metabolic process	nucleotide binding	
			LOC_Os12g31370.1	DNA repair protein Rad51, putative, expressed	1026	response to abiotic stimulus	hydrolase activity	(Morozumi et al., 2013)
	12_BLOCK_19106376_19183058	12	LOC_Os12g31840.1	ZOS12-05 - C2H2 zinc finger protein, expressed	1824	protein metabolic process	nucleic acid binding	
			LOC_Os12g31780.2	nitrilase-associated protein, putative, expressed	306	cell growth	molecular_function	
			LOC_Os12g31810.1	cyclin, putative, expressed	1473	metabolic process	metabolic process	(La et al., 2006)
			LOC_Os12g31800.1	glycine-rich RNA-binding protein 7, putative, expressed	777			
			LOC_Os12g31820.1	phosphoserine phosphatase, chloroplast precursor, putative, expressed	888	biosynthetic process	binding	
			LOC_Os12g31830.1	nitrilase, putative, expressed	972	metabolic process	hydrolase activity	
			LOC_Os12g31850.1	ureide permease, putative, expressed	1338	transport	transporter activity	
PL	2_BLOCK_35591725_35790248	2	LOC_Os02g58490.1	PINHEAD, putative, expressed	3036	response to endogenous stimulus	nuclease activity	(Wu et al., 2009)

			LOC_Os02g58440.1	zinc finger C-x8-C-x5-C-x3-H type family protein, expressed	1944	biological_process	nucleic acid binding	
			LOC_Os02g58210.1	jmjC domain containing protein, expressed	2991	biosynthetic process	sequence-specific DNA binding transcription factor activity	
			LOC_Os02g58220.1	RPA2A - Putative single-stranded DNA binding complex subunit 2, expressed	840	DNA metabolic process	protein binding	(Marwedel et al., 2003)
			LOC_Os02g58230.1	C2 domain containing protein, putative, expressed	1977			
			LOC_Os02g58260.1	metallo-beta-lactamase family protein, putative, expressed	798	metabolic process	catalytic activity	
			LOC_Os02g58270.1	metallo-beta-lactamase family protein, putative, expressed	975		hydrolase activity	
			LOC_Os02g58340.1	oligopeptidase, putative, expressed	2313	protein metabolic process	hydrolase activity	
			LOC_Os02g58350.1	OsRR3 type-A response regulator, expressed	396	nucleobase, nucleoside, nucleotide and nucleic acid metabolic process	protein binding	(Jain et al., 2006)
			LOC_Os02g58390.1	inactive receptor kinase At2g26730 precursor, putative, expressed	2073	protein modification process	nucleotide binding	
			LOC_Os02g58460.1	beta-catenin-like protein 1, putative, expressed	1551	nucleobase, nucleoside, nucleotide and nucleic acid metabolic process	binding	
			LOC_Os02g58480.1	sucrose synthase, putative, expressed	2541	biosynthetic process	transferase activity	(Hirose et al., 2008)
	2_BLOCK_35817740_35817740	2	LOC_Os02g58590.1	N-acetylglucosaminyltransferase I, putative, expressed	1347	biosynthetic process	transferase activity	(Fanata et al., 2013)

	4_BLOCK_31842220_32019553	4	LOC_Os04g53496.1	NBS-LRR disease resistance protein, putative, expressed	5424	response to stress	protein binding	
			LOC_Os04g53510.1	OsFBL20 - F-box domain and LRR containing protein, expressed	846			
			LOC_Os04g53540.3	homeobox and START domains containing protein, putative, expressed	2355	multicellular organismal development	sequence-specific DNA binding transcription factor activity	
			LOC_Os04g53550.1	ABC transporter, ATP-binding protein, putative, expressed	942	transport	transporter activity	
			LOC_Os04g53580.1	P21-Rho-binding domain containing protein, putative, expressed	498	cell growth	molecular_function	
			LOC_Os04g53612.1	APO, putative, expressed	984	biological_process	mitochondrion	
			LOC_Os04g53620.1	ubiquitin family protein, putative, expressed	1179	biological_process	protein modification process	
			LOC_Os04g53630.1	pentatricopeptide, putative, expressed	1680	biological_process	binding	
			LOC_Os04g53640.1	peroxidase precursor, putative, expressed	996	response to stress	catalytic activity	
	4_BLOCK_32144497_32252127	4	LOC_Os04g54120.1	serine/threonine-protein kinase receptor precursor, putative, expressed	2469	metabolic process	signal transducer activity	
			LOC_Os04g54080.1	serine/threonine-protein kinase receptor precursor, putative, expressed	2499	pollen-pistil interaction	nucleotide binding	
			LOC_Os04g53994.1	kinase, putative, expressed	2442	pollen-pistil interaction	nucleotide binding	
			LOC_Os04g53998.1	kinase, putative, expressed	2442	pollen-pistil interaction	nucleotide binding	
			LOC_Os04g54002.1	serine/threonine-protein kinase receptor precursor, putative, expressed	2463	metabolic process	carbohydrate binding	
			LOC_Os04g54010.1	serine/threonine-protein kinase receptor precursor, putative, expressed	2157	pollen-pistil interaction	kinase activity	
			LOC_Os04g54020.1	receptor-like kinase, putative, expressed	3108	metabolic process	nucleotide binding	
			LOC_Os04g54070.1	receptor-like kinase, putative, expressed	2664	signal transduction	receptor activity	

			LOC_Os04g54110.1	ARK3, putative, expressed	2064	pollen-pistil interaction	carbohydrate binding	
	4_BLOCK_33128044_33326841	4	LOC_Os04g55680.1	indole-3-acetate beta-glucosyltransferase, putative, expressed	1410	metabolic process	transferase activity	
			LOC_Os04g55920.1	zinc-finger protein, putative, expressed	654	biological_process	protein binding	(Cai et al., 2014; Ye et al., 2009)
			LOC_Os04g55690.1	CCB4, putative, expressed	882	cellular component organization	molecular_function	
			LOC_Os04g55700.1	exonuclease, putative, expressed	1392	metabolic process	nuclease activity	
			LOC_Os04g55720.1	D-3-phosphoglycerate dehydrogenase, chloroplast precursor, putative, expressed	1842	multicellular organismal development	nucleotide binding	
			LOC_Os04g55730.1	alpha-N-acetylglucosaminidase, putative, expressed	2391	reproduction	hydrolase activity	
			LOC_Os04g55740.1	peroxidase precursor, putative, expressed	960	response to stress	catalytic activity	
			LOC_Os04g55760.1	OsWAK55 - OsWAK receptor-like protein kinase, expressed	2352	metabolic process	protein modification process	
			LOC_Os04g55800.1	sulfate transporter, putative, expressed	1986	transport	transporter activity	(Zhao et al., 2016)
			LOC_Os04g55850.1	nuclease PA3, putative, expressed	873	catabolic process	nucleic acid binding	
			LOC_Os04g55860.1	peptidyl-tRNA hydrolase, putative, expressed	735	translation	hydrolase activity	
	S7_10979899	7						
	8_BLOCK_16164808_16356444	8	LOC_Os08g26850.1	plant protein of unknown function domain containing protein, expressed	1497			
			LOC_Os08g26710.1	plant protein of unknown function domain containing protein, expressed	1524			
			LOC_Os08g26820.1	plant protein of unknown function domain containing protein, expressed	1509			

			LOC_Os08g26840.1	plant protein of unknown function domain containing protein, expressed	1641			
	9_BLOCK_14599124_14797034	9	LOC_Os09g24710.2	alpha/beta hydrolase fold, putative, expressed	1119	biological_process		
			LOC_Os09g24690.1	60S ribosomal protein L34, putative, expressed	360	cellular process	structural molecule activity	
			LOC_Os09g24560.1	No apical meristem protein, putative, expressed	903	multicellular organismal development	sequence-specific DNA binding transcription factor activity	
			LOC_Os09g24570.1	growth regulator related protein, putative, expressed	1539	biological_process	molecular_function	
			LOC_Os09g24640.1	pentatricopeptide, putative, expressed	1512			
	10_BLOCK_10329302_10485970	10	LOC_Os09g24650.2	zinc finger, C3HC4 type, putative, expressed	834		binding	
			LOC_Os09g24660.1	AGAP000554-PA, putative, expressed	1404	biosynthetic process	binding	
			LOC_Os09g24670.1	CAAX amino terminal protease family protein, putative, expressed	1062	protein metabolic process		
			LOC_Os09g24680.1	nucleobase, nucleoside, nucleotide and nucleic acid metabolic	2433			(Gothandam et al., 2005)
			LOC_Os10g20650.1	glucan endo-1,3-beta-glucosidase-related, putative, expressed	1035	metabolic process	catalytic activity	
			LOC_Os10g20550.1	DEFL70 - Defensin and Defensin-like DEFL family, expressed	228			
			LOC_Os10g20560.1	DEFL81 - Defensin and Defensin-like DEFL family	270			
			LOC_Os10g20600.1	zinc finger, C3HC4 type domain containing protein, expressed	1083	response to endogenous stimulus	binding	
			LOC_Os10g20610.1	laccase-15 precursor, putative, expressed	1800	catabolic process		
			LOC_Os10g20630.1	vacuolar-sorting receptor precursor, putative, expressed	1896	transport	binding	

	12_BLOCK_7503930_7561908	12	LOC_Os12g13460.1	SET domain-containing protein, putative, expressed	1530			
			LOC_Os12g13440.1	phosphatidylinositol-4-phosphate 5-Kinase, putative, expressed	5034	cellular component organization	binding	
	12_BLOCK_14219148_14220620	12						
	12_BLOCK_14665324_14675306	12						
SW	7_BLOCK_10991803_11175112	7	LOC_Os07g18720.1	tetratricopeptide repeat containing protein, putative, expressed	1452	biological_process	binding	
			LOC_Os07g18710.1	OsFBLD8 - F-box, LRR and FBD domain containing protein, expressed	1347			
	9_BLOCK_19806236_19979493	9	LOC_Os09g33800.1	arabinogalactan protein, putative, expressed	621	biological_process	molecular_function	
			LOC_Os09g33680.1	Os9bglu31 - beta-glucosidase, dhurrinase, similar to G. max hydroxyisourate hydrolase, expressed	1572	carbohydrate metabolic process	catalytic activity	(Komvongsa et al., 2015)
			LOC_Os09g33600.1	ARF GTPase-activating domain-containing protein, putative, expressed	2511	multicellular organismal development	lipid binding	
			LOC_Os09g33620.1	Leucine Rich Repeat domain containing protein, expressed	1527			
			LOC_Os09g33630.2	protein kinase domain containing protein, expressed	864	response to biotic stimulus	receptor activity	(Shimizu et al., 2010)
			LOC_Os09g33690.1	Os9bglu32 - beta-glucosidase homologue, similar to G. max hydroxyisourate hydrolase, expressed	1602	metabolic process	catalytic activity	
			LOC_Os09g33710.1	Os9bglu33 - beta-glucosidase homologue, similar to G. max hydroxyisourate hydrolase, expressed	1515	metabolic process	catalytic activity	
			LOC_Os09g33720.1	protein transport protein Sec61, putative, expressed	1401	transport	transporter activity	
			LOC_Os09g33740.1	Zinc finger, ZZ type domain containing protein, expressed	1731	protein modification process	catalytic activity	

	10_BLOCK_22070293_22270204	10	LOC_Os10g41340.1	palmitoyl-protein thioesterase 1 precursor, putative, expressed	945	protein modification process	hydrolase activity	
			LOC_Os10g41220.1	protein kinase family protein, putative, expressed	2349	metabolic process	nucleotide binding	
			LOC_Os10g41130.1	AP2 domain containing protein, expressed	870	DNA binding	DNA binding	
			LOC_Os10g41170.1	dehydrogenase, putative, expressed	996	response to abiotic stimulus	binding	
			LOC_Os10g41190.1	transporter family protein, putative, expressed	1557	transport	transporter activity	
			LOC_Os10g41360.1	ARABIDILLO-1, putative, expressed	1713	post-embryonic development	catalytic activity	
			LOC_Os10g41230.1	homeobox associated leucine zipper, putative, expressed	936	biosynthetic process	sequence-specific DNA binding transcription factor activity	(Meijer et al., 1997; Scarpella et al., 2000)
			LOC_Os10g41240.1	dual specificity protein phosphatase, putative, expressed	1089	protein modification process	hydrolase activity	
			LOC_Os10g41250.1	glycoprotein, putative, expressed	1476			
			LOC_Os10g41260.1	MYB family transcription factor, putative, expressed	798	response to endogenous stimulus	sequence-specific DNA binding transcription factor activity	(Yang et al., 2016)
			LOC_Os10g41310.1	DUF630/DUF632 domains containing protein, putative, expressed	2304	biological_process	DNA binding	
ST	1_BLOCK_10275866_10473381	1	LOC_Os01g18440.1	OsMADS89 - MADS-box family gene with M-gamma type-box, expressed	921	nucleobase, nucleoside, nucleotide and nucleic acid metabolic process	sequence-specific DNA binding transcription factor activity	
			LOC_Os01g18320.1	protoporphyrinogen oxidase, chloroplast precursor, putative, expressed	1611	metabolic process	catalytic activity	

			LOC_Os01g18360.1	OsIAA4 - Auxin-responsive Aux/IAA gene family member, expressed	612	response to abiotic stimulus	sequence-specific DNA binding transcription factor activity	
			LOC_Os01g18390.1	OsRCI2-1 - Putative low temperature and salt responsive protein, expressed	873			
	2_BLOCK_24571861_24661819	2	LOC_Os02g40664.1	zinc finger family protein, putative, expressed	710	biosynthetic process	binding	
			LOC_Os02g40550.1	Lung seven transmembrane receptor domain containing protein, putative, expressed	1395	biological_process	molecular_function	
			LOC_Os02g40530.1	MYB family transcription factor, putative, expressed	870	biosynthetic process	sequence-specific DNA binding transcription factor activity	(Schmidt et al., 2013)
	4_BLOCK_31842220_32019553	4	LOC_Os04g53680.1	cyclin, putative, expressed	795	protein modification process	protein binding	(La et al., 2006)
			LOC_Os04g53496.1	NBS-LRR disease resistance protein, putative, expressed	5424	response to stress	protein binding	
			LOC_Os04g53510.1	OsFBL20 - F-box domain and LRR containing protein, expressed	846			
			LOC_Os04g53540.3	homeobox and START domains containing protein, putative, expressed	2355	cell differentiation	sequence-specific DNA binding transcription factor activity	
			LOC_Os04g53550.1	ABC transporter, ATP-binding protein, putative, expressed	942	transport	transporter activity	
			LOC_Os04g53580.1	P21-Rho-binding domain containing protein, putative, expressed	498	cell growth	molecular_function	
			LOC_Os04g53612.1	APO, putative, expressed	984	biological_process	mitochondrion	
			LOC_Os04g53620.1	ubiquitin family protein, putative, expressed	1179	biological_process	protein modification process	

			LOC_Os04g53630.1	pentatricopeptide, putative, expressed	1680	biological_process		
			LOC_Os04g53640.1	peroxidase precursor, putative, expressed	996	response to stress	catalytic activity	
	S6_27632618	6						
	7_BLOCK_5179524_5348724	7	LOC_Os07g09970.1	LTPL84 - Protease inhibitor/seed storage/LTP family protein precursor, expressed	624	transport	lipid binding	
			LOC_Os07g09740.1	no apical meristem protein, expressed	1995	multicellular organismal development	sequence-specific DNA binding transcription factor activity	
			LOC_Os07g09814.2	OsFBX221 - F-box domain containing protein, expressed	1215			
			LOC_Os07g09830.1	no apical meristem protein, expressed	2121	biosynthetic process	sequence-specific DNA binding transcription factor activity	
			LOC_Os07g09860.1	no apical meristem protein, expressed	2037	biological_process	sequence-specific DNA binding transcription factor activity	
			LOC_Os07g09870.1	OsFBA2 - F-box and FBA domain containing protein, expressed	1149			
			LOC_Os07g09890.1	hexokinase, putative, expressed	1530	carbohydrate metabolic process	response to abiotic stimulus	(Cho et al., 2006)
			LOC_Os07g09900.1	disease resistance protein RPM1, putative, expressed	2082	response to stress	nucleotide binding	
	7_BLOCK_24385360_24576268	7	LOC_Os07g41014.1	glycosyl hydrolases family 17 protein, expressed	1998	metabolic process	hydrolase activity	
			LOC_Os07g41060.1	dihydroflavonol-4-reductase, putative, expressed	675	biosynthetic process	binding	
			LOC_Os07g40710.2	circadian clock coupling factor-related, putative, expressed	747	biological_process	molecular_function	
			LOC_Os07g40730.1	SNF2 domain-containing protein, putative, expressed	3501			(Hu et al., 2013)

			LOC_Os07g40740.1	heparanase-like protein precursor, putative, expressed	1590	metabolic process	hydrolase activity	
			LOC_Os07g40750.1	PPR repeat domain containing protein, putative, expressed	1521	biological_process	molecular_function	
			LOC_Os07g40770.1	myosin heavy chain-related, putative, expressed	1365	biological_process	molecular_function	
			LOC_Os07g40790.1	COBW domain containing protein, putative, expressed	1095	biological_process	molecular_function	
			LOC_Os07g40800.1	PPR repeat domain containing protein, putative, expressed	1872	biological_process	molecular_function	
			LOC_Os07g40810.1	NBS-LRR type disease resistance protein, putative, expressed	2946	response to stress	nucleotide binding	
			LOC_Os07g40986.1	NAD dependent epimerase/dehydratase family protein, putative, expressed	1008	metabolic process	catalytic activity	
			LOC_Os07g40940.1	X8 domain containing protein, expressed	585	biological_process	carbohydrate binding	
	S11_19707591	11						
	11_BLOCK_20480700_20564349	11	LOC_Os11g35040.1	aminotransferase, classes I and II, domain containing protein, expressed	1329	cellular process	transferase activity	(Inoue et al., 2008)
			LOC_Os11g34970.1	NB-ARC domain containing protein, expressed	1527	response to abiotic stimulus	nucleotide binding	
			LOC_Os11g34990.1	DEFL79 - Defensin and Defensin-like DEFL family	261			
			LOC_Os11g35030.1	growth regulating factor protein, putative, expressed	1230	multicellular organismal development	protein binding	(Choi et al., 2004)
			LOC_Os11g35040.1	aminotransferase, classes I and II, domain containing protein, expressed	1329	cellular process	transferase activity	
			LOC_Os11g35020.2	yippee zinc-binding protein, putative, expressed	321	biological_process	molecular_function	
SL	2_BLOCK_12695457_12894785	2	LOC_Os02g21650.1	prefoldin, putative, expressed	681	transport	molecular_function	
			LOC_Os02g21550.1	flavonol synthase/flavanone 3-hydroxylase, putative, expressed	660	metabolic process	catalytic activity	

			LOC_Os02g21660.1	L1P family of ribosomal proteins domain containing protein, expressed	651	nucleobase, nucleoside, nucleotide and nucleic acid metabolic	structural molecule activity	
			LOC_Os02g21430.1	AML1, putative, expressed	2436	biological_process	nucleic acid binding	
			LOC_Os02g21460.1	uncharacterized protein yqjG, putative, expressed	1095	biological_process	molecular_function	
			LOC_Os02g21490.1	WD domain, G-beta repeat domain containing protein, expressed	1356	biological_process	molecular_function	
			LOC_Os02g21500.1	PE-PGRS family protein, putative, expressed	1101	metabolic process	catalytic activity	
			LOC_Os02g21510.1	NOL1/NOP2/sun family protein, putative, expressed	1185			
			LOC_Os02g21520.1	chalcone isomerase 3, putative, expressed	843	biosynthetic process	catalytic activity	
			LOC_Os02g21560.1	retrotransposon protein, putative, unclassified, expressed	3843			
			LOC_Os02g21660.1	L1P family of ribosomal proteins domain containing protein, expressed	651	nucleobase, nucleoside, nucleotide and nucleic acid metabolic	RNA binding	
			LOC_Os02g21580.1	PPR repeat containing protein, expressed	1578	biological_process	molecular_function	
			LOC_Os02g21590.1	retrotransposon, putative, centromere-specific, expressed	3999			
			LOC_Os02g21630.1	SEC14 cytosolic factor family protein, putative, expressed	786	transport	transporter activity	
	3_BLOCK_10477564_10651116	3	LOC_Os03g18970.1	protein phosphotase protein, putative, expressed	1299	metabolic process	catalytic activity	
			LOC_Os03g18890.1	glycosyl transferase 8 domain containing protein, putative, expressed	1107	carbohydrate metabolic process	transferase activity	
			LOC_Os03g18779.1	expressed protein	714			

			LOC_Os03g18740.1	oxidoreductase, short chain dehydrogenase/reductase family, putative, expressed	1002	metabolic process	catalytic activity	
			LOC_Os03g18790.1	SAG20, putative, expressed	1833	biological_process	molecular_function	
			LOC_Os03g18770.1	wound-induced protein WI12, putative, expressed	375	response to stress	protein binding	
			LOC_Os03g18810.1	aminotransferase, classes I and II, domain containing protein, expressed	1395	metabolic process	binding	
			LOC_Os03g18820.1	glycosyltransferase, putative, expressed	1347	biosynthetic process	transferase activity	(Wang et al., 2014a)
			LOC_Os03g18830.1	programmed cell death protein 2, putative, expressed	1260		binding	
			LOC_Os03g18840.1	NUC189 domain containing protein, expressed	1914	biological_process	nucleotide binding	
			LOC_Os03g18970.1	protein phosphotase protein, putative, expressed	1299	metabolic process	hydrolase activity	
			LOC_Os03g18870.1	heat shock protein DnaJ, putative, expressed	501	cellular process	protein binding	
			LOC_Os03g18910.1	COBRA-like protein 7 precursor, putative, expressed	2016	biological_process	molecular_function	
			LOC_Os03g18940.1	exonuclease, putative, expressed	942	metabolic process	nuclease activity	
			LOC_Os03g18950.1	zinc finger C-x8-C-x5-C-x3-H type family protein, expressed	1374	biosynthetic process	nucleic acid binding	
			LOC_Os03g18960.1	calmodulin binding protein, putative, expressed	1527	biological_process	protein binding	
	4_BLOCK_29858777_29965891	4	LOC_Os04g50200.1	OsFBX151 - F-box domain containing protein, expressed	1533			
			LOC_Os04g50150.1	retrotransposon protein, putative, unclassified, expressed	2685			
			LOC_Os04g50090.1	helix-loop-helix DNA-binding protein, putative, expressed	1089	nucleobase, nucleoside, nucleotide and nucleic acid metabolic process	DNA binding	

			LOC_Os04g50080.1	expressed protein	654			
			LOC_Os04g50120.1	zinc-binding protein, putative, expressed	750		binding	
			LOC_Os04g50100.1	RING-H2 finger protein ATL5G, putative, expressed	1152	response to abiotic stimulus		
			LOC_Os04g50110.1	RNA recognition motif containing protein, putative, expressed	876		RNA binding	
			LOC_Os04g50140.1	retrotransposon protein, putative, unclassified	1479			
			LOC_Os04g50120.1	RNA recognition motif containing protein, putative, expressed	750			
			LOC_Os04g50160.1	retrotransposon protein, putative, unclassified, expressed	6084			
			LOC_Os04g50200.1	zinc-binding protein, putative, expressed	750		binding	
			LOC_Os04g50172.1	retrotransposon protein, putative, unclassified, expressed	2355			
	5_BLOCK_6791185_6982283	5	LOC_Os05g12130.1	EMB2261, putative, expressed	2469	post-embryonic development		
			LOC_Os05g12030.1	expressed protein	846			
			LOC_Os05g11950.1	GDSL-like lipase/acylhydrolase, putative, expressed	1113	metabolic process	hydrolase activity	
			LOC_Os05g11970.1	GDSL-like lipase/acylhydrolase, putative, expressed	1143	metabolic process	hydrolase activity	
			LOC_Os05g11980.1	timeless protein, expressed	3624	biological_process		
			LOC_Os05g11990.1	TTL1, putative, expressed	1446	response to endogenous stimu	binding	
			LOC_Os05g12060.1	retrotransposon protein, putative, unclassified, expressed	3882			
			LOC_Os05g12170.1	plant-specific domain TIGR01589 family protein, putative, expressed	1053	biological_process	molecular_function	
			LOC_Os05g12140.1	Leucine Rich Repeat family protein, expressed	1821			
	11_BLOCK_23861754_23986595	11	LOC_Os11g40170.1	conserved hypothetical protein	312	translation	translation factor activity, nucleic acid binding	

			LOC_Os11g40160.1	expressed protein	402			
			LOC_Os11g40060.1	retrotransposon protein, putative, unclassified, expressed	5736			
			LOC_Os11g40070.1	expressed protein	1200			
			LOC_Os11g40090.2	A49-like RNA polymerase I associated factor family protein, expressed	1488	biosynthetic process	DNA binding	
			LOC_Os11g40080.1	lipin, N-terminal conserved region family protein, expressed	4164	response to extracellular stimulus	hydrolase activity	(Li et al., 2015)
			LOC_Os11g40100.3	GRF-interacting factor 2, putative, expressed	618	biological_process	protein binding	(Liu et al., 2014)
			LOC_Os11g40150.1	DNA repair protein Rad51, putative, expressed	1020	metabolic process	nucleotide binding	(Morozumi et al., 2013)
			LOC_Os11g40140.1	peptidase, T1 family, putative, expressed	714	metabolic process	hydrolase activity	
			LOC_Os11g40180.1	dirigent, putative, expressed	891	response to stress	molecular_function	
	S12_7434458	12						
	12_BLOCK_12967864_13140942	12	LOC_Os12g23200.1	photosystem I reaction center subunit XI, chloroplast precursor, putative, expressed	627	generation of precursor metabolites and energy	molecular_function	
			LOC_Os12g23120.1	retrotransposon protein, putative, Ty3-gypsy subclass, expressed	4272			
			LOC_Os12g22980.1	retrotransposon protein, putative, Ty3-gypsy subclass, expressed	2166			
			LOC_Os12g22990.1	retrotransposon protein, putative, Ty3-gypsy subclass, expressed	5262			
			LOC_Os12g23090.1	no apical meristem protein, putative, expressed	1215	multicellular organismal development	sequence-specific DNA binding transcription factor activity	
			LOC_Os12g23040.1	transposon protein, putative, Mutator sub-class, expressed	1803			
			LOC_Os12g23030.1	retrotransposon protein, putative, unclassified, expressed	3018			

			LOC_Os12g23100.1	retrotransposon protein, putative, unclassified, expressed	1941			
			LOC_Os12g23110.1	retrotransposon protein, putative, unclassified, expressed	2484			
			LOC_Os12g23130.1	retrotransposon protein, putative, Ty3-gypsy subclass, expressed	3732			
			LOC_Os12g23170.1	Os12bglu38 - beta-glucosidase/beta-mannosidase/exoglucanase homologue, expressed	1479	carbohydrate metabolic process	hydrolase activity	
			LOC_Os12g23180.1	3-beta hydroxysteroid dehydrogenase/isomerase family protein, putative, expressed	1131	response to biotic stimulus	catalytic activity	
			LOC_Os12g23190.1	tyrosine phosphatase family protein, putative, expressed	615	metabolic process	hydrolase activity	
	12_BLOCK_13663061_13861944	12	LOC_Os12g24140.1	cyclin-dependent kinase G-2, putative, expressed	960	metabolic process	kinase activity	
			LOC_Os12g24250.1	retrotransposon protein, putative, unclassified, expressed	3252			
			LOC_Os12g24080.1	HECT-domain domain containing protein, expressed	3003	cellular process	catalytic activity	
			LOC_Os12g24100.1	retrotransposon protein, putative, unclassified	2550			
			LOC_Os12g24170.1	beta-galactosidase precursor, putative, expressed	2784	metabolic process	binding	
			LOC_Os12g24120.1	retrotransposon protein, putative, unclassified	2763			
			LOC_Os12g24110.1	retrotransposon protein, putative, Ty3-gypsy subclass, expressed	4971			
			LOC_Os12g24140.1	cyclin-dependent kinase G-2, putative, expressed	960	metabolic process	nucleotide binding	
			LOC_Os12g24260.1	transposon protein, putative, CACTA, En/Spm sub-class, expressed	2973			
			LOC_Os12g24240.1	expressed protein	393	biological_process	molecular_function	
			LOC_Os12g24230.1	expressed protein	447			

			LOC_Os12g24210.1	retrotransposon protein, putative, unclassified, expressed	2109			
			LOC_Os12g24220.1	retrotransposon protein, putative, unclassified, expressed	4794			
TGW	1_BLOCK_28709309_28893652	1	LOC_Os01g50120.2	expressed protein	915	biological_process	molecular_function	
			LOC_Os01g50030.1	CPuORF25 - conserved peptide uORF-containing transcript, expressed	1500	pollination	transferase activity	(Yu et al., 2014)
			LOC_Os01g49980.1	retrotransposon protein, putative, unclassified, expressed	4518			
			LOC_Os01g50010.1	clathrin assembly protein, putative, expressed	1707	cellular component organization	binding	
			LOC_Os01g50020.1	expressed protein	1872	translation	nucleic acid binding	
			LOC_Os01g50040.1	DNA binding protein, putative, expressed	1179			
			LOC_Os01g50050.1	polyprenyl synthetase, putative, expressed	1071	biosynthetic process	transferase activity	
			LOC_Os01g50060.1	1-aminocyclopropane-1-carboxylate deaminase, putative, expressed	909	metabolic process	binding	
			LOC_Os01g50080.1	MDR-like ABC transporter, putative, expressed	3465	cell growth	transporter activity	
			LOC_Os01g50090.1	retrotransposon protein, putative, unclassified, expressed	2817			
			LOC_Os01g50100.1	ABC transporter, ATP-binding protein, putative, expressed	3546	response to endogenous stimulus	transporter activity	
	S1_28920012	1						
	1_BLOCK_31318752_31502481	1	LOC_Os01g54470.1	GDSL-like lipase/acylhydrolase, putative, expressed	1236	metabolic process	hydrolase activity	
			LOC_Os01g54510.1	MAC/Perforin domain containing protein, putative, expressed	1662	biological_process		
			LOC_Os01g54480.1	serine/threonine protein kinase, putative, expressed	1407	metabolic process	kinase activity	

			LOC_Os01g54490.1	osFTL9 FT-Like9 homologous to Flowering Locus T gene; contains Pfam profile PF01161: Phosphatidylethanolamine-binding protein	528	post-embryonic development	lipid binding	
			LOC_Os01g54515.1	peptide transporter PTR2, putative, expressed	1587	transport	transporter activity	
			LOC_Os01g54520.2	DUF1264 domain containing protein, putative, expressed	759	biological_process	molecular_function	
			LOC_Os01g54530.1	hydrolase, acting on carbon-nitrogen, putative, expressed	1410			
			LOC_Os01g54540.1	ribosomal protein L13, putative, expressed	702	translation	structural molecule activity	(Song et al., 2014)
			LOC_Os01g54550.1	HSF-type DNA-binding domain containing protein, expressed	1323	biological_process	sequence-specific DNA binding transcription factor activity	(Shim et al., 2009; Wang et al., 2009)
			LOC_Os01g54560.1	trehalose synthase, putative, expressed	2742	biosynthetic process	hydrolase activity	
			LOC_Os01g54580.1	transporter family protein, putative, expressed	1416			
			LOC_Os01g54590.1	ras-related protein, putative, expressed	666	transport	nucleotide binding	
			LOC_Os01g54600.1	WRKY13, expressed	951	biosynthetic process	sequence-specific DNA binding transcription factor activity	(Xie et al., 2005)
			LOC_Os01g54670.1	coiled-coil domain-containing protein 25, putative, expressed	648	biological_process	protein binding	
	S3_6948281	3						
	3_BLOCK_9599101_9781950	3	LOC_Os03g17520.1	CDP-alcohol phosphatidyltransferase, putative, expressed	990	biosynthetic process	transferase activity	
			LOC_Os03g17370.1	white-brown complex homolog protein, putative, expressed	2298	transport	hydrolase activity	

			LOC_Os03g17300.1	TKL_IRAK_CrRLK1L-1.7 - The CrRLK1L-1 subfamily has homology to the CrRLK1L homolog, expressed	2532	cellular component organization	kinase activity	
			LOC_Os03g17310.1	calcium-transporting ATPase, endoplasmic reticulum-type, putative, expressed	3189	transport	transporter activity	
			LOC_Os03g17340.1	expressed protein	1425	biological_process	binding	
			LOC_Os03g17350.1	white-brown complex homolog protein, putative, expressed	2364	transport	transporter activity	(Yasuno et al., 2009)
			LOC_Os03g17410.1	expressed protein	3090			
			LOC_Os03g17432.1	pre-mRNA-splicing factor ATP-dependent RNA helicase DHX16, putative, expressed	1215	metabolic process	nucleic acid binding	
			LOC_Os03g17450.1	nucleotide binding protein, putative, expressed	717	nucleotide binding	biological_process	
			LOC_Os03g17460.1	IN2-1 protein, putative, expressed	885	protein modification process		
			LOC_Os03g17470.1	IN2-1 protein, putative, expressed	738	protein modification process	plastid	
			LOC_Os03g17480.1	IN2-1 protein, putative, expressed	732	biological_process		
			LOC_Os03g17510.1	pentatricopeptide, putative, expressed	1797	biological_process		
	3_BLOCK_10991463_11168887	3	LOC_Os03g19760.1	HAD-superfamily hydrolase, subfamily IA, variant 3 containing protein, expressed	3195	cellular homeostasis	hydrolase activity	
			LOC_Os03g19590.1	phytochrome B, putative, expressed	3516	signal transduction	protein binding	(Takano et al., 2005)
			LOC_Os03g19610.1	pectinesterase, putative, expressed	1038	metabolic process	hydrolase activity	
			LOC_Os03g19650.3	PPR2, putative, expressed	1506	pollination		
			LOC_Os03g19670.1	GDSL-like lipase/acylhydrolase, putative, expressed	1104	metabolic process	hydrolase activity	
			LOC_Os03g19680.1	enoyl-CoA hydratase/isomerase family protein, putative, expressed	939	metabolic process	catalytic activity	
			LOC_Os03g19690.1	expressed protein	2454	biological_process	molecular_function	
	3_BLOCK_23155569_23343573	3	LOC_Os03g41920.1	expressed protein	999			

			LOC_Os03g41740.1	retrotransposon protein, putative, unclassified, expressed	1008			
	3_BLOCK_32969173_33157926	3	LOC_Os03g58120.1	PPR repeat domain containing protein, putative, expressed	2235	post-embryonic developme		
			LOC_Os03g57950.1	type I inositol-1,4,5-trisphosphate 5-phosphatase, putative, expressed	1698	cellular process	hydrolase activity	
			LOC_Os03g57940.2	CK1_CaseinKinase_1a.4 - CK1 includes the casein kinase 1 kinases, expressed	2124	metabolic process	nucleotide binding	(Dai and Xue, 2010; Hori et al., 2013)
			LOC_Os03g57980.1	LTPL99 - Protease inhibitor/seed storage/LTP family protein precursor, expressed	600	transport	lipid binding	
			LOC_Os03g58040.1	glutamate dehydrogenase protein, putative, expressed	1236	metabolic process	catalytic activity	
			LOC_Os03g58060.1	XRN 5'-3' exonuclease N-terminus domain containing protein, expressed	2967	cell growth	nucleic acid binding	
	5_BLOCK_6791185_6982283	5	LOC_Os03g58170.1	stem-specific protein TSJT1, putative, expressed	750	response to endogenous stimulus	molecular_function	
			LOC_Os03g58150.1	transmembrane BAX inhibitor motif-containing protein, putative, expressed	726	cellular component organization	binding	
			LOC_Os03g58130.1	thioredoxin, putative, expressed	576			
			LOC_Os05g12170.1	plant-specific domain TIGR01589 family protein, putative, expressed	1053	biological_process	molecular_function	
			LOC_Os05g11950.1	GDSL-like lipase/acylhydrolase, putative, expressed	1113	metabolic process	lipid metabolic process	
			LOC_Os05g11970.1	GDSL-like lipase/acylhydrolase, putative, expressed	1143	metabolic process	hydrolase activity	
			LOC_Os05g11980.1	timeless protein, expressed	3624	biological_process		
			LOC_Os05g11990.1	TTL1, putative, expressed	1446	response to endogenous stimulus	binding	

			LOC_Os05g12040.1	cytochrome P450 51, putative, expressed	1503	post-embryonic development	oxygen binding	(Xia et al., 2015)
			LOC_Os05g12130.1	EMB2261, putative, expressed	2469	reproduction		
			LOC_Os05g12140.1	Leucine Rich Repeat family protein, expressed	1821			
			LOC_Os05g12150.1	endoglucanase precursor, putative, expressed	1890	carbohydrate metabolic process	metabolic process	
	5_BLOCK_29004623_29203515	5	LOC_Os05g50710.1	late embryogenesis abundant protein, putative, expressed	456	reproduction	molecular function	(He et al., 2012)
			LOC_Os05g50660.1	PX domain containing protein, putative, expressed	3411	signal transduction	lipid binding	
			LOC_Os05g50690.1	pentatricopeptide, putative, expressed	1404	biological process		
			LOC_Os05g50750.1	AAA family ATPase, putative, expressed	2568	metabolic process	hydrolase activity	
			LOC_Os05g50840.1	mitochondrial carrier protein, putative, expressed	1068	transport	transporter activity	
			LOC_Os05g50810.1	CAMK_CAMK_like.5 - CAMK includes calcium/calmodulin depedent protein kinases, expressed	1698	response to endogenous stimulus	kinase activity	
			LOC_Os05g50740.1	transposon protein, putative, unclassified, expressed	4722			
			LOC_Os05g50880.1	transposon protein, putative, CACTA, En/Spm sub-class, expressed	2796			
	9_BLOCK_15762553_15959818	9	LOC_Os09g26260.1	AAA-type ATPase family protein, putative, expressed	1572	metabolic process	hydrolase activity	
			LOC_Os09g26180.1	transcription initiation factor TFIID subunit 10, putative, expressed	498	biosynthetic process	sequence-specific DNA binding transcription factor activity	
			LOC_Os09g26240.1	transposon protein, putative, CACTA, En/Spm sub-class, expressed	3474			
			LOC_Os09g26380.1	aminotransferase, classes I and II, domain containing protein, expressed	1461		transferase activity	
			LOC_Os09g26360.1	pectinesterase, putative, expressed	1854	metabolic process	hydrolase activity	

			LOC_Os09g26310.1	hypro1, putative, expressed	942	protein modification process	transferase activity	
			LOC_Os09g26290.1	amino acid transporter family protein, putative, expressed	927	transport	transporter activity	
	9_BLOCK_20925868_21081274	9	LOC_Os09g36470.1	retrotransposon protein, putative, unclassified, expressed	2586			
			LOC_Os09g36340.1	HVA22, putative, expressed	567	biological_process	molecular_function	
			LOC_Os09g36290.1	Ser/Thr protein phosphatase family protein, putative, expressed	1197	hydrolase activity	hydrolase activity	
			LOC_Os09g36320.1	tyrosine protein kinase domain containing protein, putative, expressed	1221	metabolic process	nucleotide binding	(Dubouzet et al., 2011)
			LOC_Os09g36350.1	endoglucanase, putative, expressed	1587	metabolic process	hydrolase activity	
			LOC_Os09g36370.1	ER lumen protein retaining receptor containing protein, expressed	1599	signal transduction	receptor activity	
			LOC_Os09g36400.1	expressed protein	1791			(Zhu et al., 2002)
			LOC_Os09g36420.1	hsp90 protein, expressed	3141	response to stress	protein binding	
			LOC_Os09g36440.1	transcription initiation factor IIB, putative, expressed	939	translation	translation factor activity, nucleic acid binding	
			LOC_Os09g36450.1	triosephosphate isomerase, chloroplast precursor, putative, expressed	915	reproduction	protein binding	
			LOC_Os09g36460.1	zinc RING finger protein, putative, expressed	978			
	11_BLOCK_20176616_20356516	11	LOC_Os11g34700.1	ZOS11-04 - C2H2 zinc finger protein, expressed	2532	protein metabolic process	hydrolase activity	
			LOC_Os11g34500.1	retrotransposon protein, putative, unclassified, expressed	5403			
			LOC_Os11g34624.1	TKL_IRAK_DUF26-lc.29 - DUF26 kinases have homology to DUF26 containing loci, expressed	1791	metabolic process	carbohydrate binding	
			LOC_Os11g34680.1	RNA recognition motif containing protein, putative, expressed	873	metabolic process	nucleotide binding	

	11_BLOCK_23861754_23986595	11	LOC_Os11g40130.1	expressed protein	585			
			LOC_Os11g40050.1	retrotransposon protein, putative, unclassified, expressed	3303			
			LOC_Os11g40090.2	A49-like RNA polymerase I associated factor family protein, expressed	1488	biosynthetic process	DNA binding	(Li et al., 2015)
			LOC_Os11g40080.1	lipin, N-terminal conserved region family protein, expressed	4164	cell communication	hydrolase activity	
			LOC_Os11g40140.1	peptidase, T1 family, putative, expressed	714	metabolic process	hydrolase activity	
			LOC_Os11g40180.1	dirigent, putative, expressed	891	response to stress	molecular_function	
			LOC_Os11g40150.1	DNA repair protein Rad51, putative, expressed	1020	metabolic process	nucleotide binding	(Morozumi et al., 2013)
	11_BLOCK_26916905_27078112	11	LOC_Os11g44690.1	cysteine-rich receptor-like protein kinase 19 precursor, putative, expressed	1440	biological_process	nucleotide binding	
			LOC_Os11g44560.1	protein kinase domain containing protein, expressed	2982	cellular process	protein binding	
			LOC_Os11g44600.1	calmodulin binding protein, putative, expressed	1770			
			LOC_Os11g44580.1	go35 NBS-LRR, putative, expressed	2445	response to stress	protein binding	
			LOC_Os11g44550.1	protein kinase family protein, putative, expressed	918	metabolic process	kinase activity	
			LOC_Os11g44630.1	calmodulin binding protein, putative, expressed	1131			
			LOC_Os11g44680.1	calmodulin binding protein, putative, expressed	1857	biological_process	protein binding	
			LOC_Os11g44660.1	protein kinase, putative, expressed	1371	metabolic process	kinase activity	
			LOC_Os11g44700.1	calmodulin binding protein, putative, expressed	1158	biological_process	protein binding	
GY	3_BLOCK_6520556_6531124	3	LOC_Os03g12260.1	cytochrome P450 protein, putative, expressed	1623	metabolic process	oxygen binding	
			LOC_Os03g12360.1	trehalose-6-phosphate synthase, putative, expressed	2583	carbohydrate metabolic process	transferase activity	
			LOC_Os03g12270.1	dehydrogenase, putative, expressed	1089	cell death	binding	
			LOC_Os03g12300.1	HEAT repeat family protein, putative, expressed	2136	biological_process	binding	

			LOC_Os03g12320.1	UDP-3-O- N-acetylglucosamine deacetylase, putative, expressed	966	carbohydrate metabolic process	hydrolase activity	
			LOC_Os03g12350.1	two-component response regulator, putative, expressed	2076	response to endogenous stimulus	sequence-specific DNA binding transcription factor activity	
			LOC_Os03g12370.1	HSF-type DNA-binding domain containing protein, expressed	1233	response to abiotic stimulus	sequence-specific DNA binding transcription factor activity	(Ito and Kurata, 2006)
			LOC_Os03g12390.1	STE_MEK_ste7_MAP2K.6 - STE kinases include homologs to sterile 7, sterile 11 and sterile 20 from yeast, expressed	1020	biosynthetic process	enzyme regulator activity	(Mittal et al., 2009)
			LOC_Os03g12414.2	cyclin, putative, expressed	1410	protein modification process	kinase activity	(Wu et al., 2015)
			LOC_Os03g12430.1	PPR repeat containing protein, putative, expressed	1413			
			LOC_Os03g12440.1	zinc-binding protein, putative, expressed	897			
			LOC_Os03g12450.1	nucleoporin, putative, expressed	3873			
	3_BLOCK_6520556_6531124	3	LOC_Os03g25790.1	glycosyl hydrolases family 17 protein, expressed	1437	metabolic process	binding	
			LOC_Os03g25600.2	protein phosphatase 2C, putative, expressed	2934	protein modification process	hydrolase activity	
			LOC_Os03g25620.1	LTV1, putative, expressed	1503	biological_process	molecular_process	
			LOC_Os03g25640.1	OsFBX91 - F-box domain containing protein, expressed	1281			
			LOC_Os03g25650.1	OsFBX92 - F-box domain containing protein, expressed	1254			
			LOC_Os03g25720.1	GA20734-PA, putative, expressed	834			
			LOC_Os03g25750.1	SNARE associated Golgi protein, putative, expressed	1041	biological_process	molecular_function	

			LOC_Os03g25760.1	calmodulin-binding protein, putative, expressed	1734	biological_process		
			LOC_Os03g25770.1	RNA recognition motif containing protein, putative, expressed	657	nucleobase, nucleoside, nucleotide and nucleic acid metabolic	RNA binding	
	5_BLOCK_6791185_6982283	5	LOC_Os05g11950.1	GDSL-like lipase/acylhydrolase, putative, expressed	1113	metabolic process	hydrolase activity	
			LOC_Os05g11970.1	GDSL-like lipase/acylhydrolase, putative, expressed	1143	metabolic process	hydrolase activity	
			LOC_Os05g11980.1	timeless protein, expressed	3624	biological_process		
			LOC_Os05g11990.1	TTL1, putative, expressed	1446	response to endogenous stimulus	binding	
			LOC_Os05g12040.1	cytochrome P450 51, putative, expressed	1503	biosynthetic process	catalytic activity	(Xia et al., 2015)
			LOC_Os05g12130.1	EMB2261, putative, expressed	2469	post-embryonic development		
			LOC_Os05g12140.1	Leucine Rich Repeat family protein, expressed	1821			
			LOC_Os05g12150.1	endoglucanase precursor, putative, expressed	1890	metabolic process	catalytic activity	
			LOC_Os05g12170.1	plant-specific domain TIGR01589 family protein, putative, expressed	1053	biological_process	molecular_function	
	10_BLOCK_426788_526921	10	LOC_Os10g01680.1	transferase family protein, putative, expressed	1491	metabolic process	transferase activity	
			LOC_Os10g01660.1	transferase family protein, putative, expressed	1395	metabolic process	transferase activity	
			LOC_Os10g01720.1	transferase family protein, putative, expressed	1527	biological_process	metabolic process	
			LOC_Os10g01690.1	transferase family protein, putative, expressed	1305	biological_process	transferase activity	

			LOC_Os10g01770.1	retrotransposon protein, putative, unclassified, expressed	3255			
			LOC_Os10g01750.1	retrotransposon protein, putative, unclassified, expressed	4053			
			LOC_Os10g01690.1	transferase family protein, putative, expressed	1305	biological_process	transferase activity	
	11_BLOCK_23861754_23986595	11	LOC_Os11g40080.1	lipin, N-terminal conserved region family protein, expressed	4164	metabolic process	hydrolase activity	
			LOC_Os11g40090.2	A49-like RNA polymerase I associated factor family protein, expressed	1488	biosynthetic process	transferase activity	(Li et al., 2015)
			LOC_Os11g40100.3	GRF-interacting factor 2, putative, expressed	618	multicellular organismal development	molecular_function	(Liu et al., 2014)
			LOC_Os11g40110.1	RWP-RK, putative, expressed	750	biosynthetic process	sequence-specific DNA binding transcription factor activity	
			LOC_Os11g40140.1	peptidase, T1 family, putative, expressed	714	protein metabolic process	hydrolase activity	
			LOC_Os11g40180.1	dirigent, putative, expressed	891			
			LOC_Os11g40060.1	retrotransposon protein, putative, unclassified, expressed	5736			
			LOC_Os11g40050.1	retrotransposon protein, putative, unclassified, expressed	3303			
	12_BLOCK_13997092_14180506	12	LOC_Os12g24540.1	PHD-finger family protein, expressed	3807	biological_process	binding	
			LOC_Os12g24580.1	Lg106, putative, expressed	288	biological_process	molecular functions	
			LOC_Os12g24650.1	leucine aminopeptidase, chloroplast precursor, putative, expressed	1629	metabolic process	binding	
			LOC_Os12g24580.1	Lg106, putative, expressed	288	biological_process	molecular function	
			LOC_Os12g24550.1	expressed protein	4041	anatomical structure morphogenes		

	12_BLOCK_14219148_14220620	12	LOC_Os12g24790.1	retrotransposon protein, putative, LINE subclass, expressed	3342			
			LOC_Os12g24790.1	retrotransposon protein, putative, LINE subclass, expressed	3342			

Supplementary figure 1 (a) Biological and molecular functions of genes related to SNPLDBs of GCA of days to heading

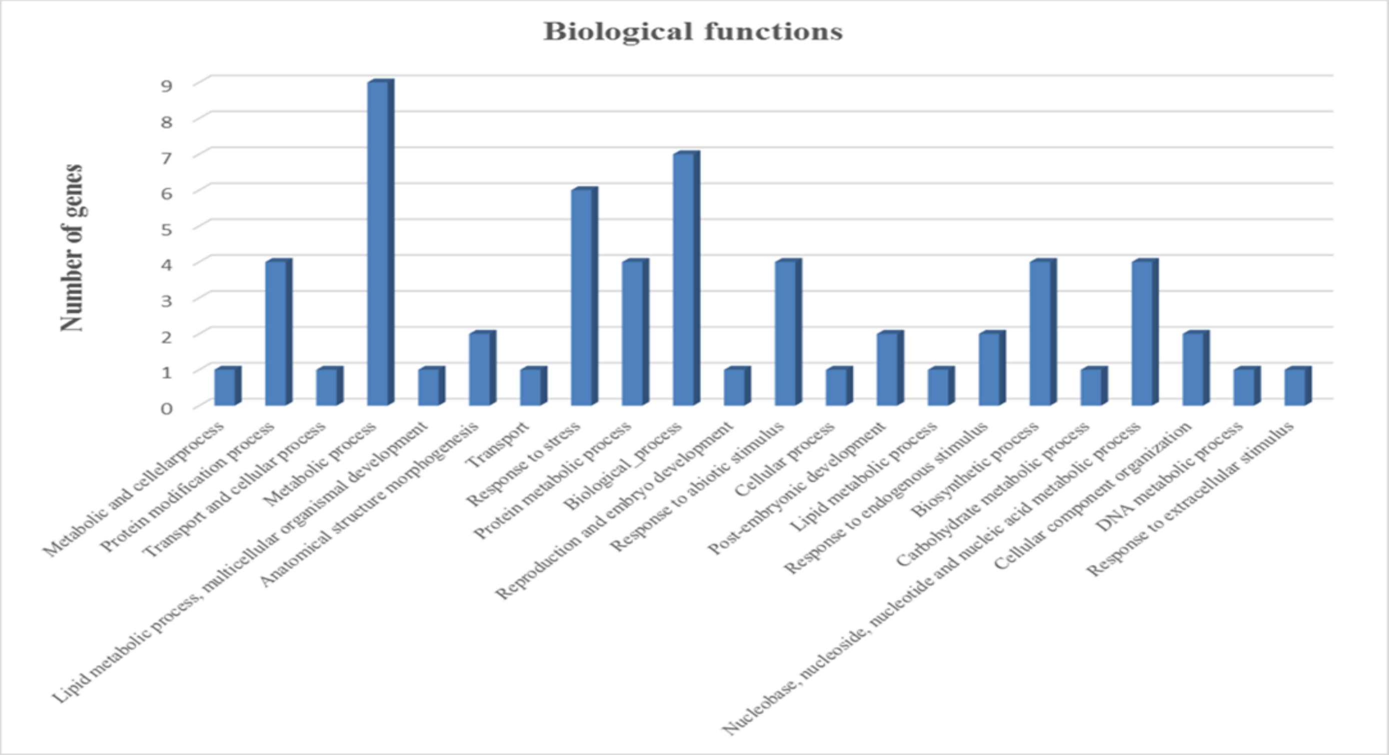

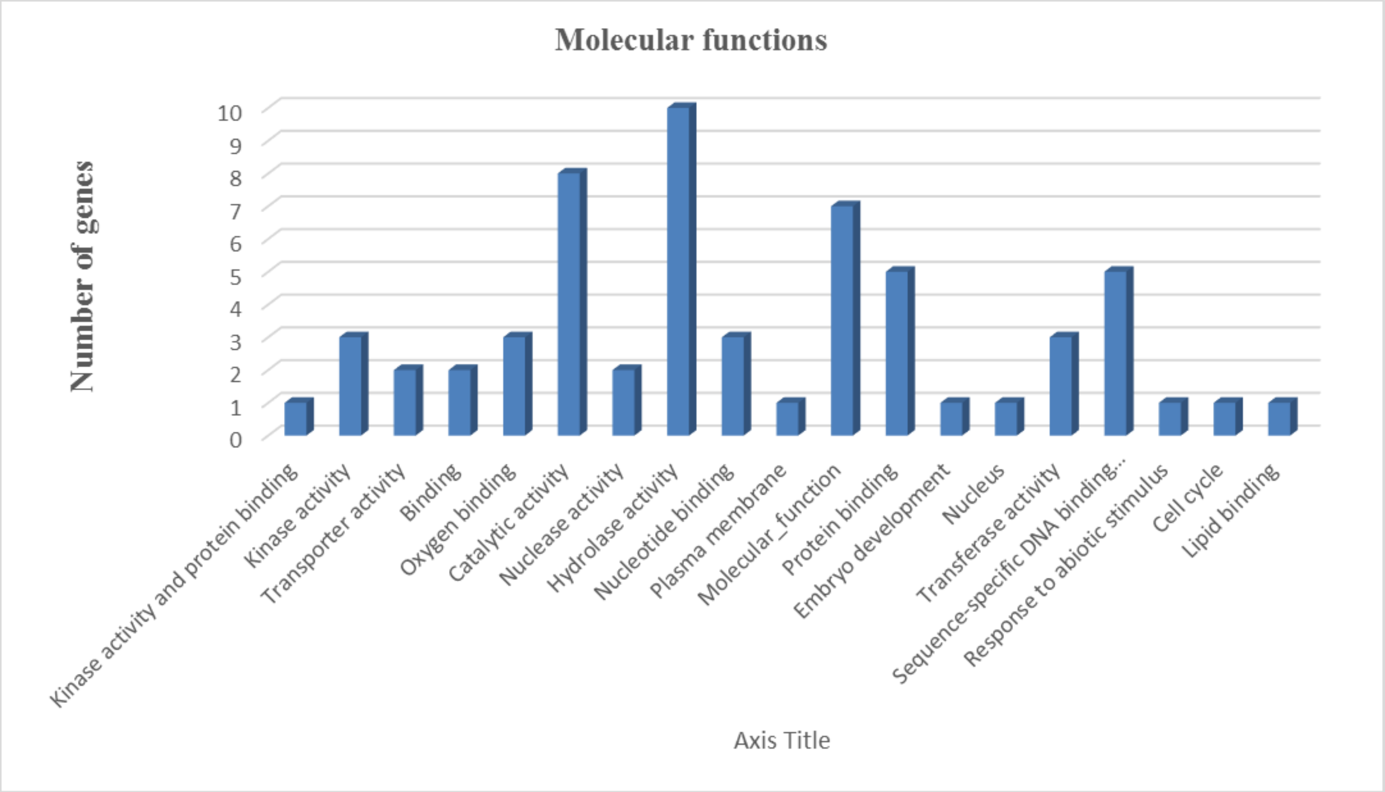

Supplementary figure 1 (b) Biological and molecular functions of genes related to SNPLDBs of GCA of plant height

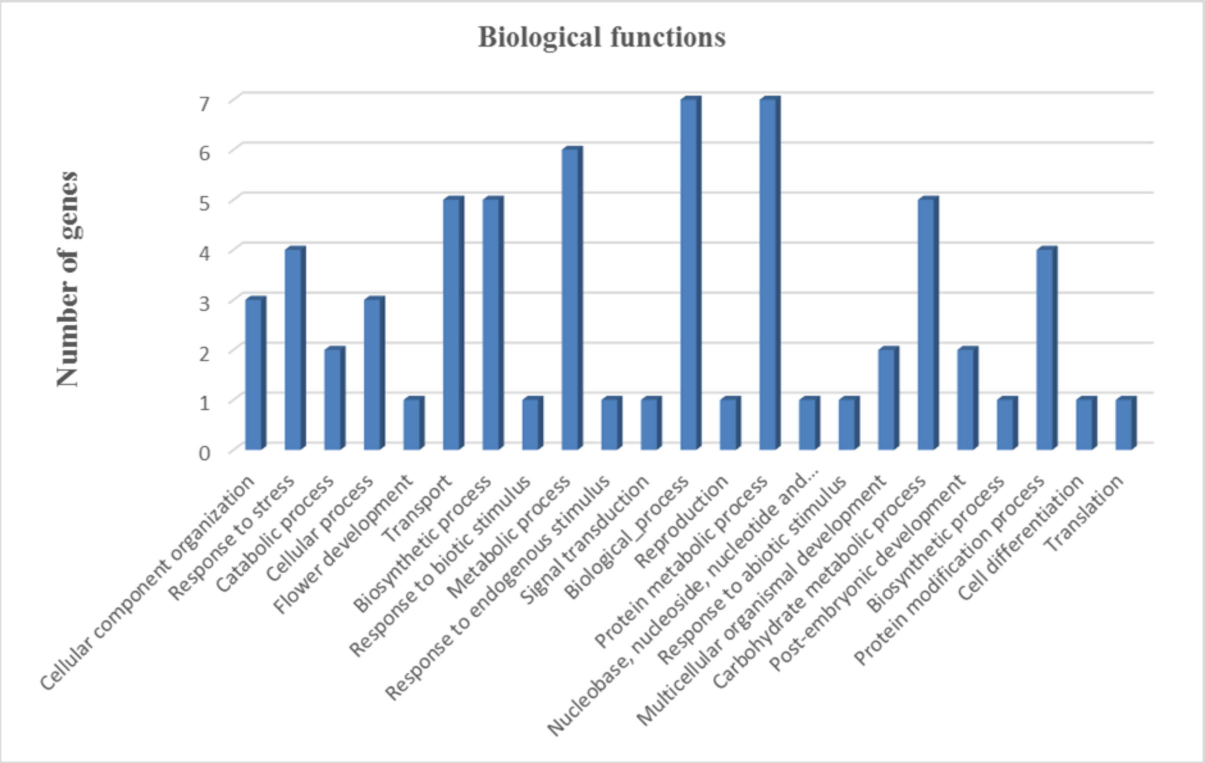

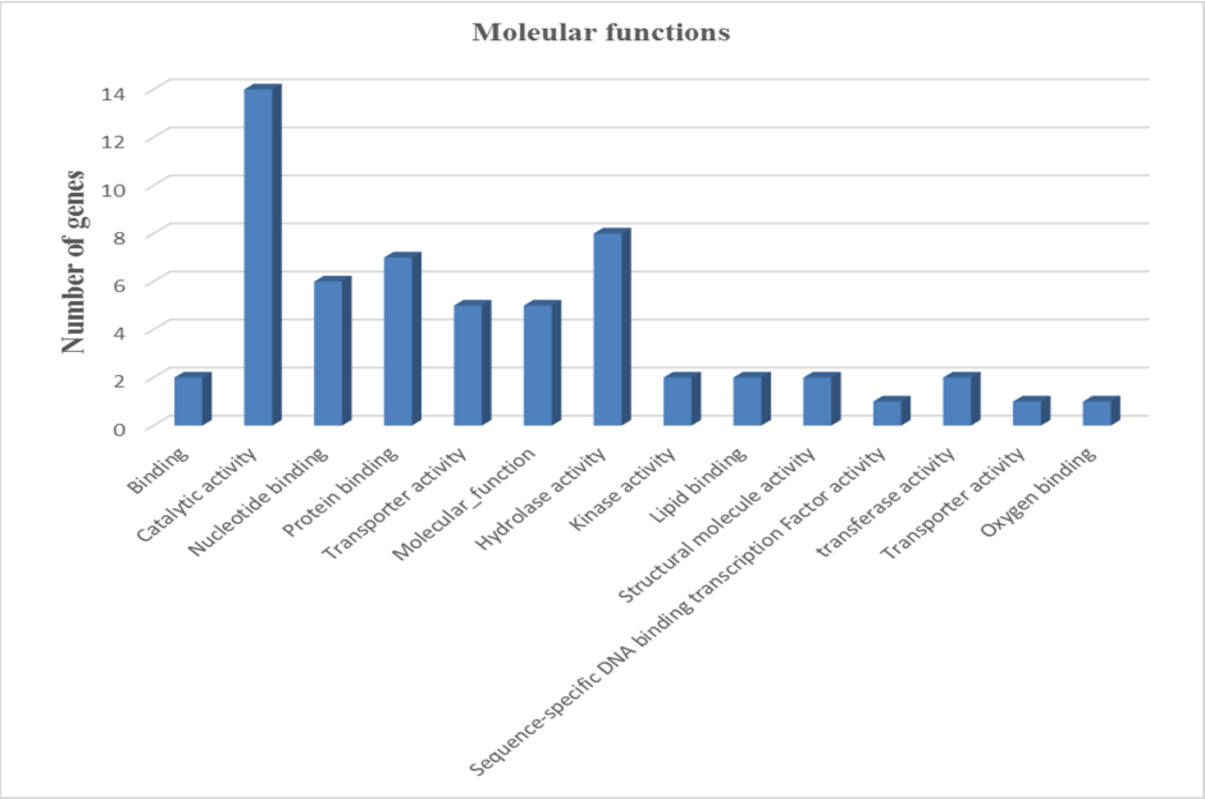

Supplementary figure 1 (c) Biological and molecular functions of genes related to SNPLDBs of GCA of number of panicles per plant

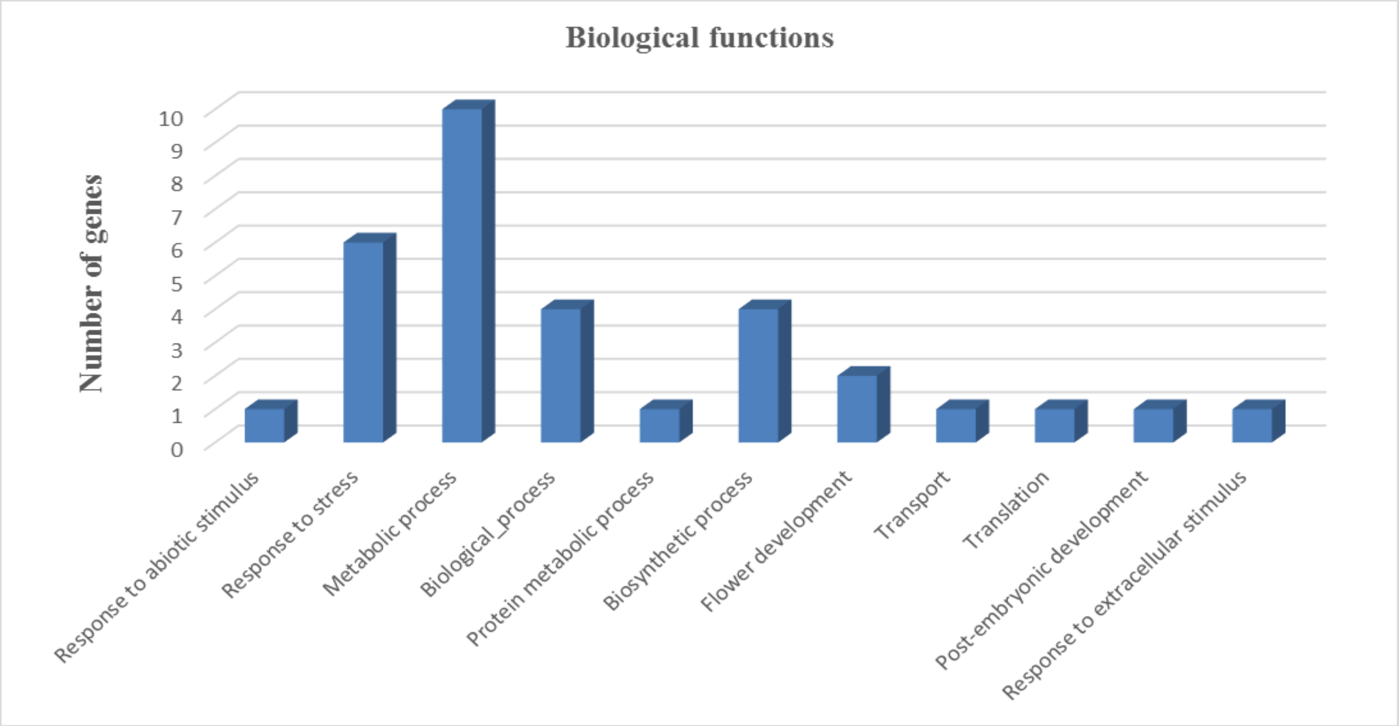

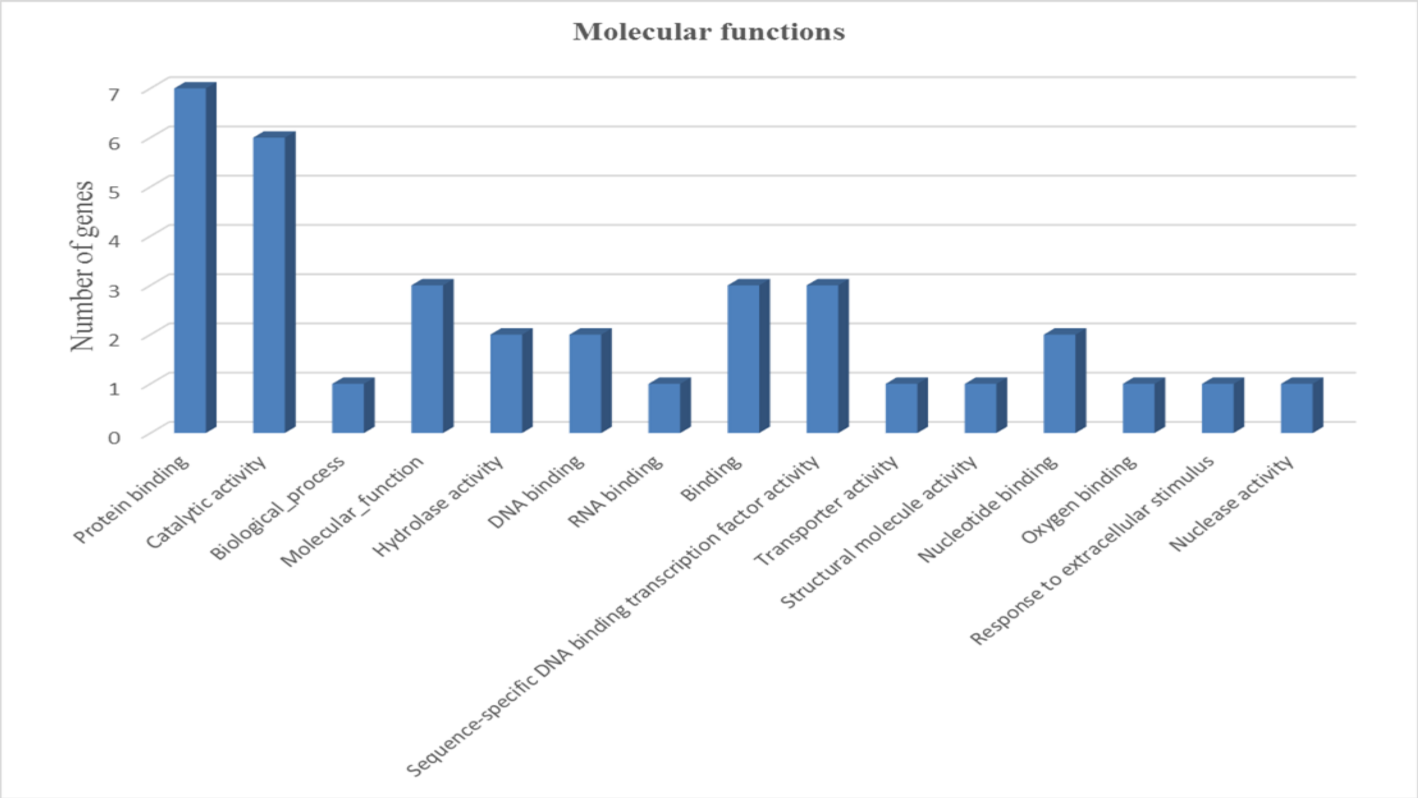

Supplementary figure 1 (d) Biological and molecular functions of genes related to SNPLDBs of GCA of number of filled grain per panicles

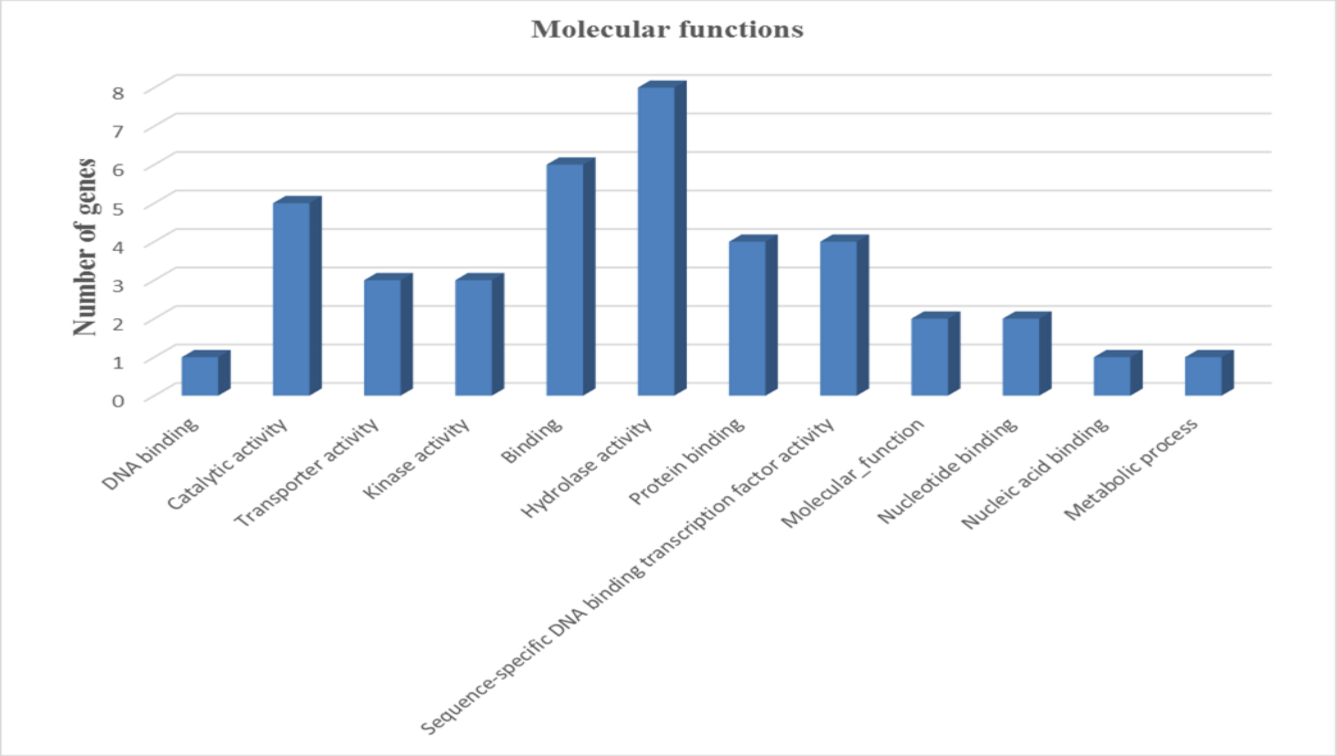

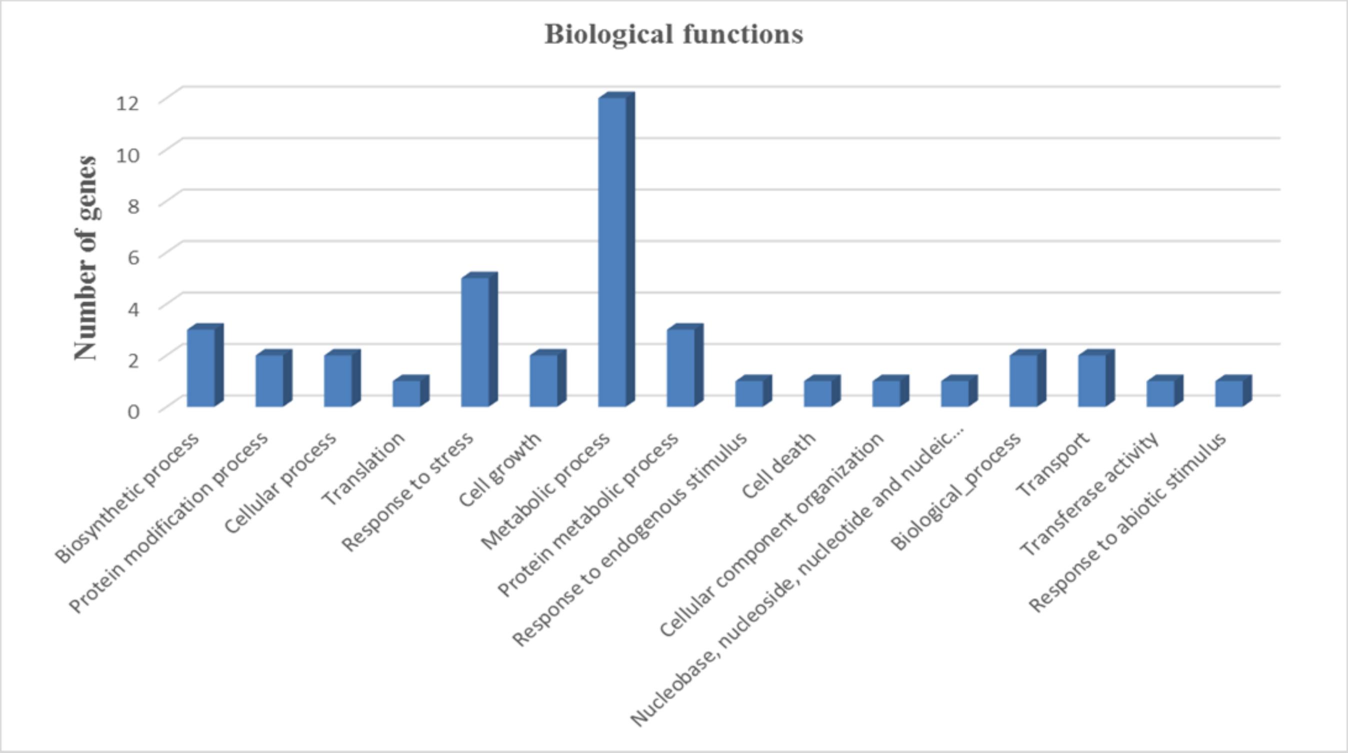

Supplementary figure 1 (e) Biological and molecular functions of genes related to SNPLDBs of GCA of panicle length

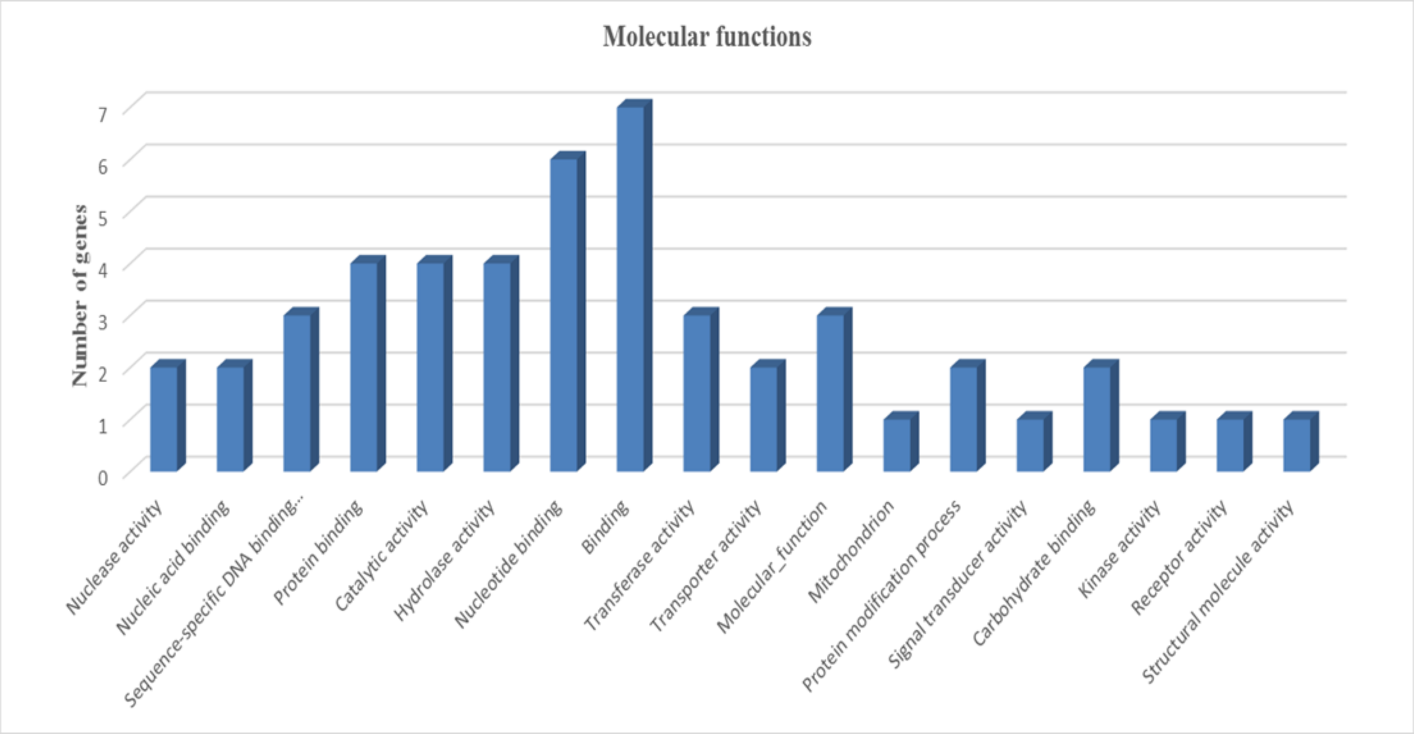

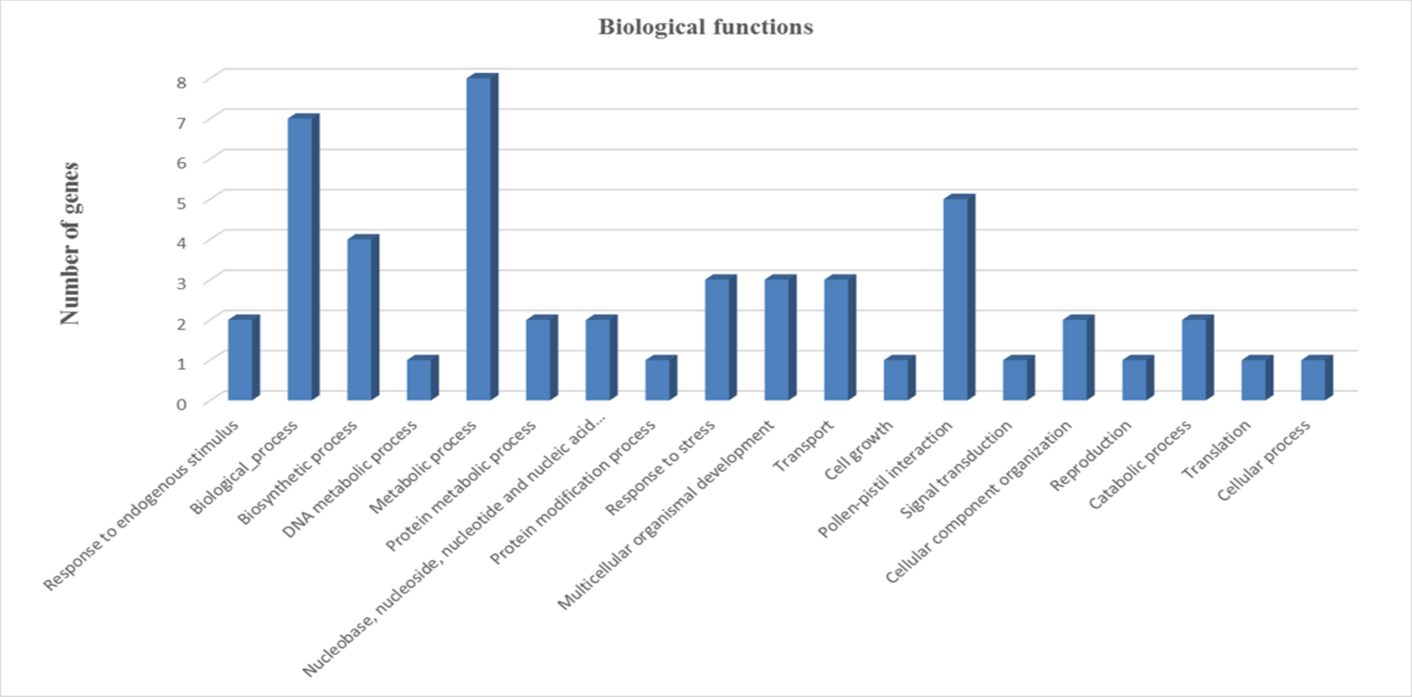

Supplementary figure 1 (f) Biological and molecular functions of genes related to SNPLDBs of GCA of number of seed width

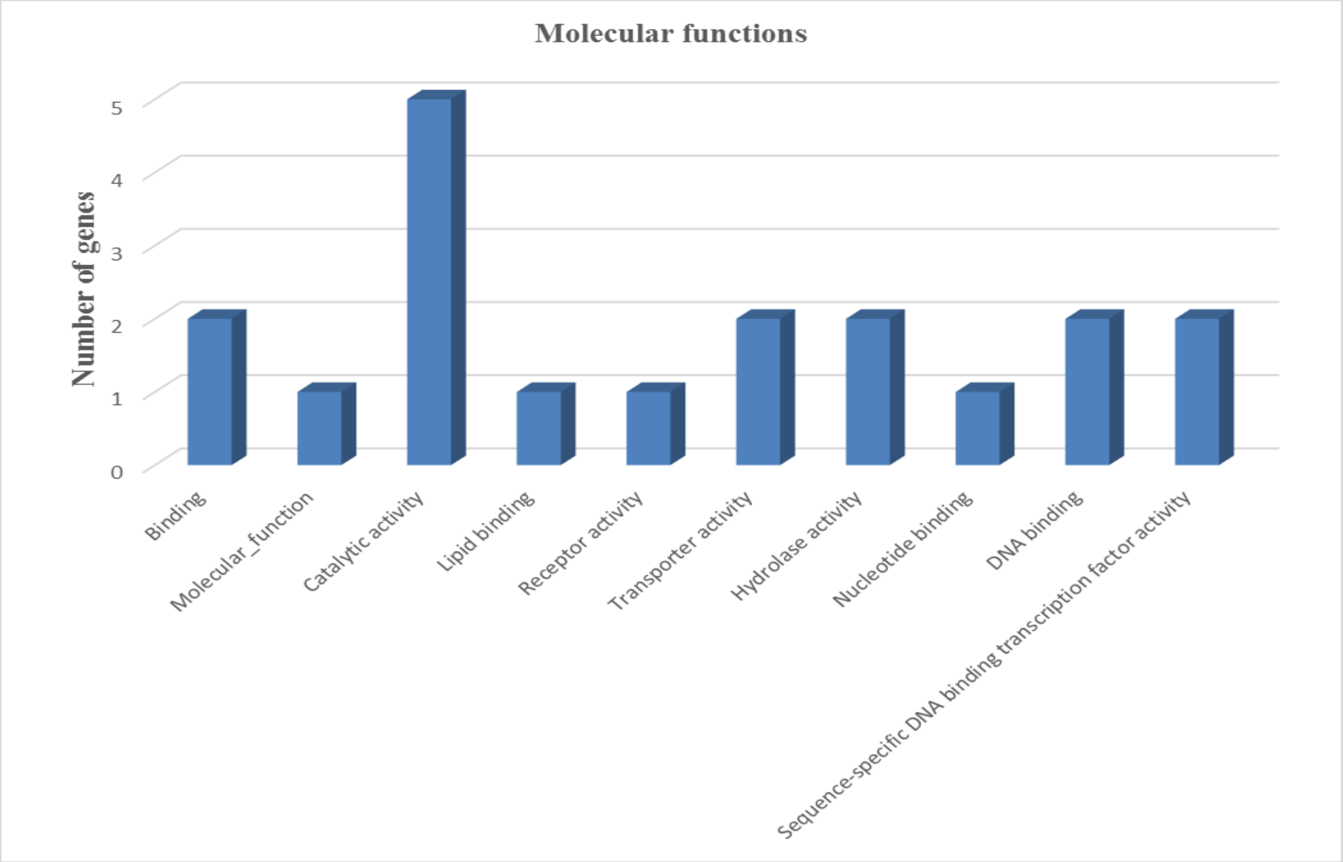

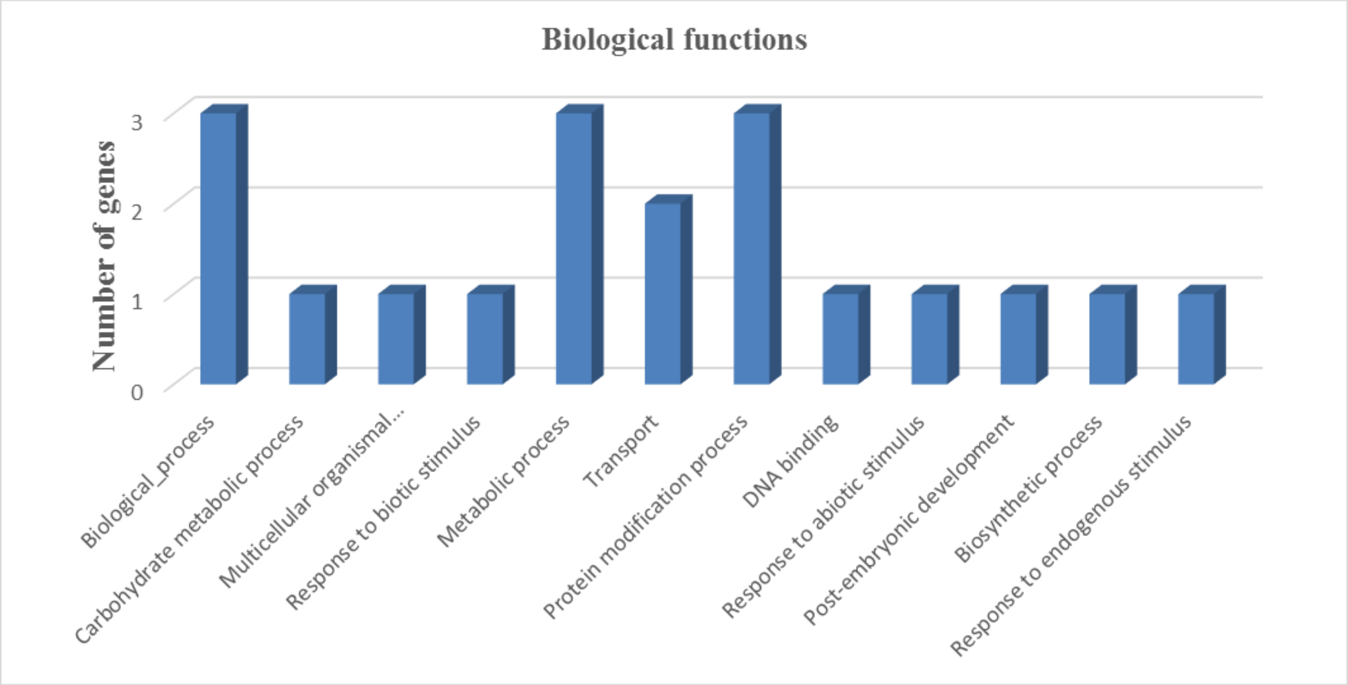

Supplementary figure 1 (g) Biological and molecular functions of genes related to SNPLDBs of GCA of number of seed thickness

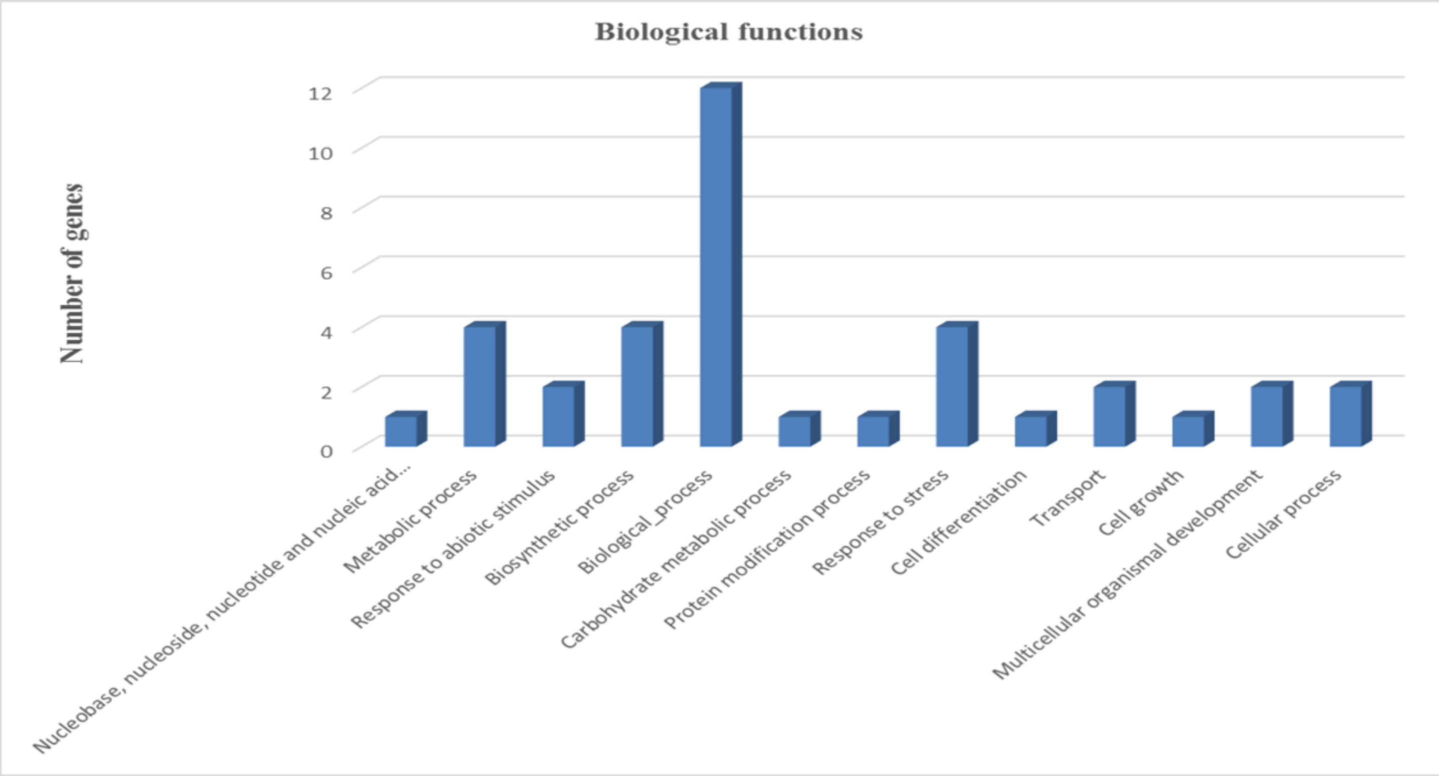

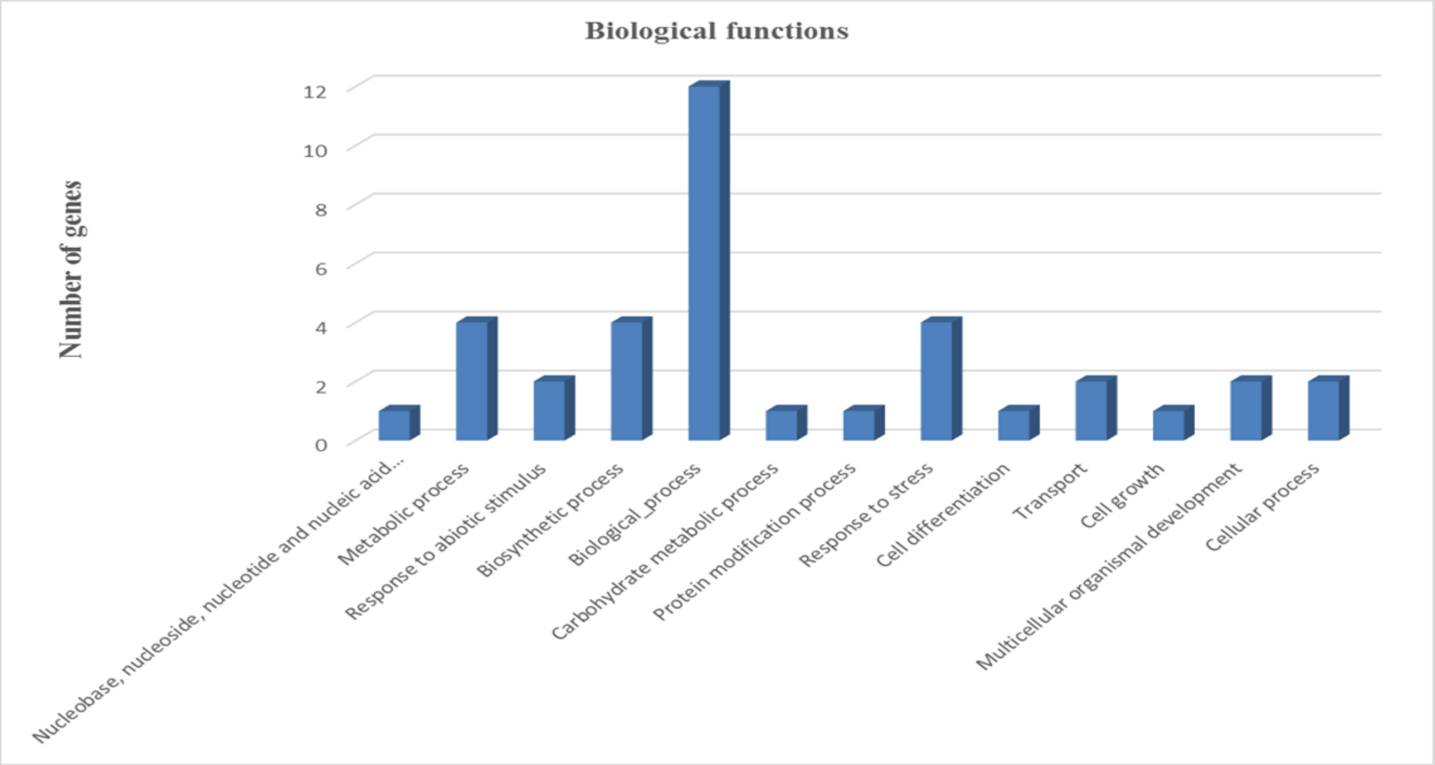

Supplementary figure 1 (h) Biological and molecular functions of genes related to SNPLDBs of GCA of number of seed length

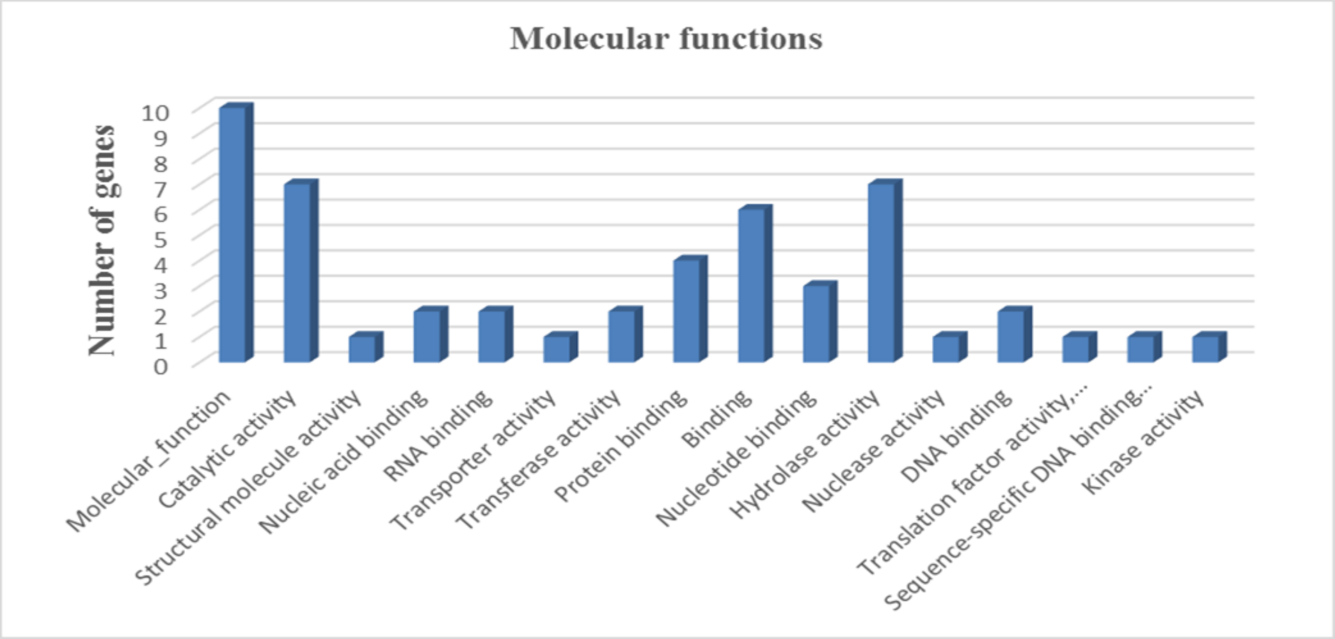

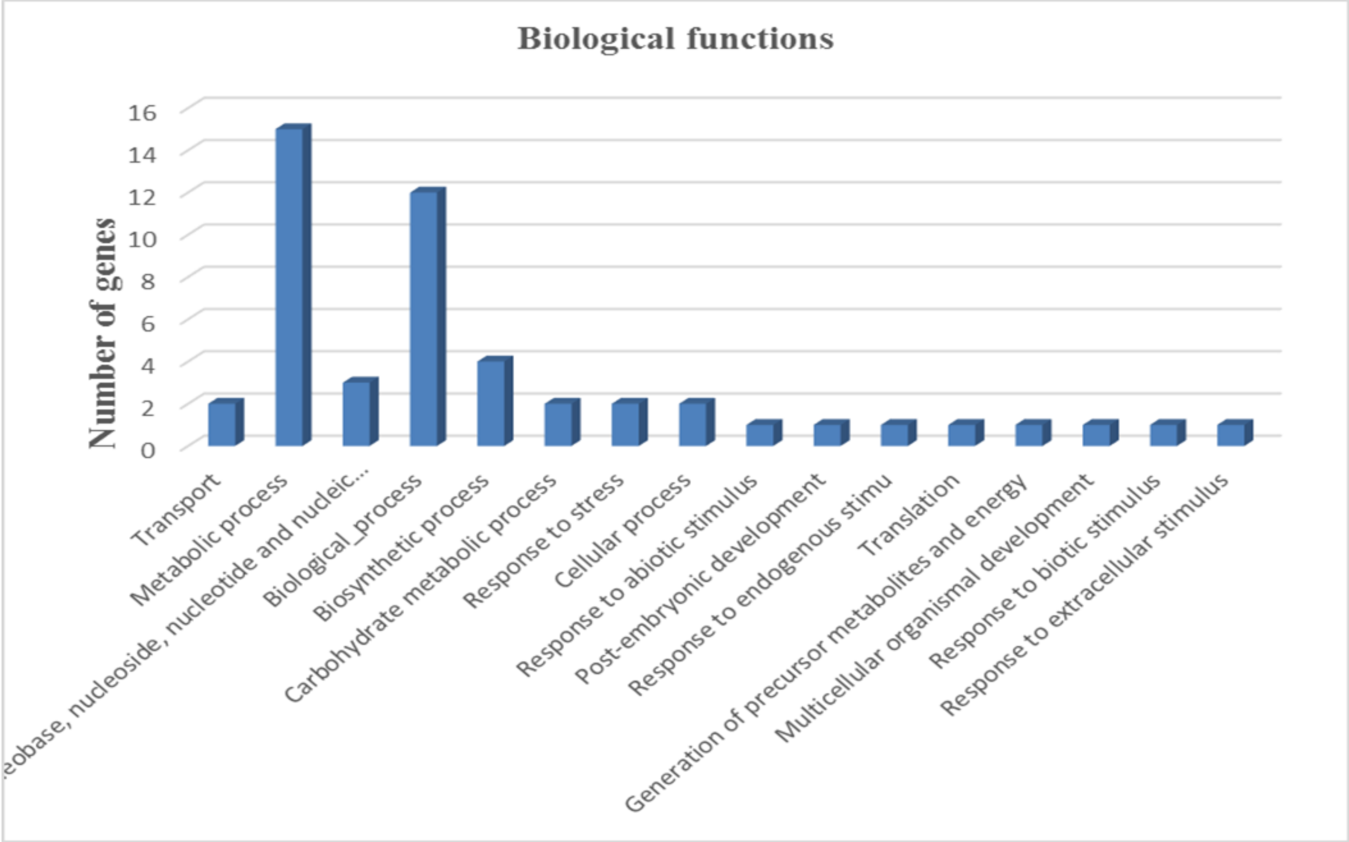

Supplementary figure 1 (i) Biological and molecular functions of genes related to SNPLDBs of GCA of number of thousand seed weight

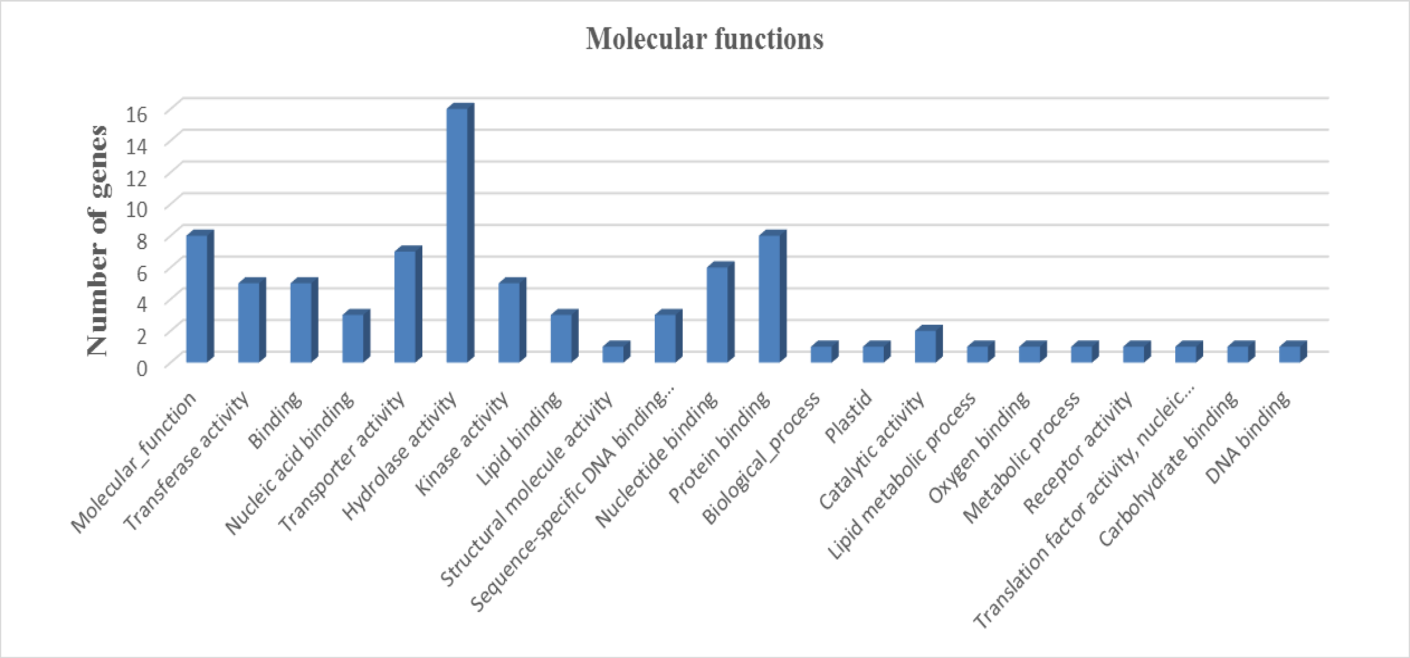

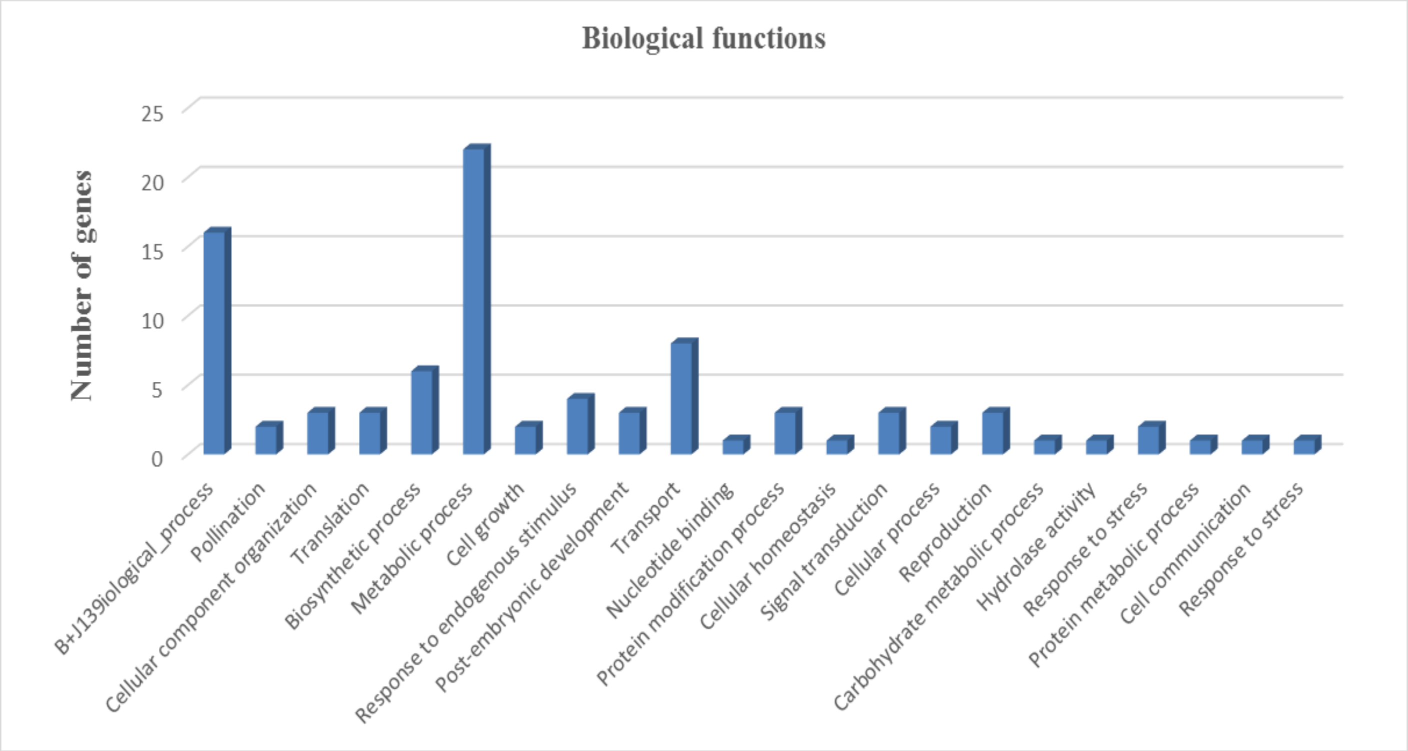

Supplementary figure 1 (j) Biological and molecular functions of genes related to SNPLDBs of GCA of number of grain yield per plot

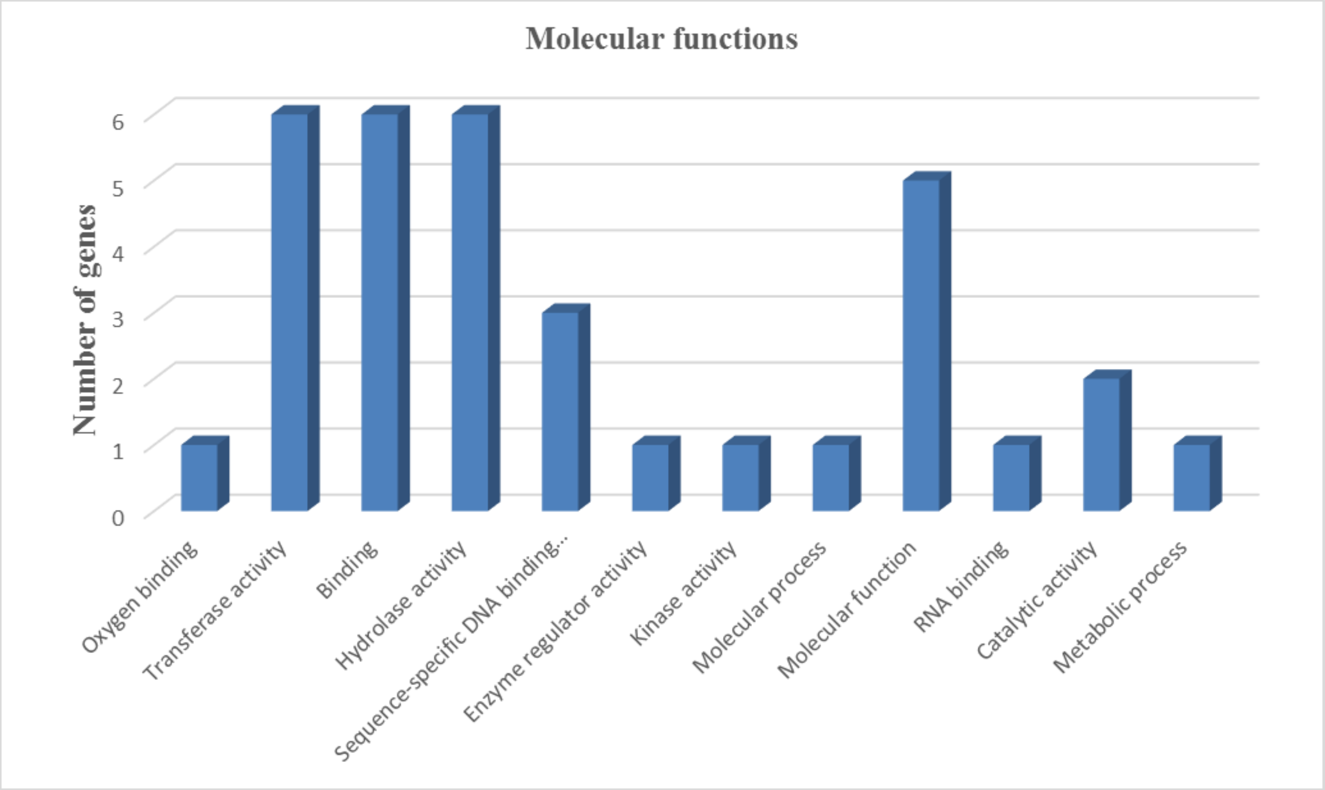

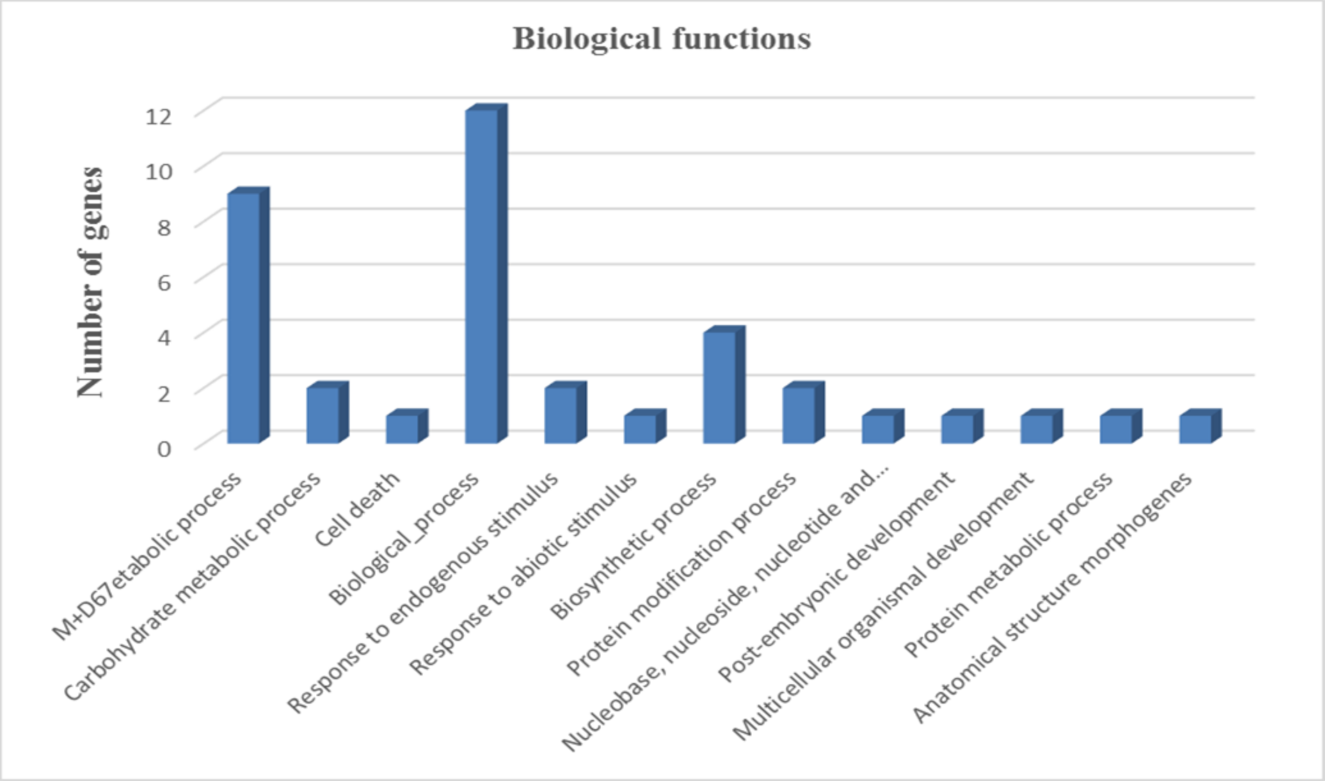

Supplement: Supplimentary Material [file ply077_suppl_supplementary_material.pdf]
